# Transient and intermediate carbocations in ruthenium tetroxide oxidation of saturated rings

**DOI:** 10.3762/bjoc.15.158

**Published:** 2019-07-11

**Authors:** Manuel Pedrón, Laura Legnani, Maria-Assunta Chiacchio, Pierluigi Caramella, Tomás Tejero, Pedro Merino

**Affiliations:** 1 Instituto de Biocomputación y Física de Sistemas Complejos (BIFI), Campus San Francisco, Universidad de Zaragoza, 50009 Zaragoza, Spain; 2 Dipartimento di Scienze del Farmaco, University of Catania, V.le A. Doria 6, 95125 Catania, Italy; 3 Dipartimento di Chimica, Università di Pavia, Via Taramelli, 12, 27100, Pavia, Italy; 4 Instituto de Síntesis Química y Catálisis Homogénea (ISQCH), Campus San Francisco, Universidad de Zaragoza-CSIC, 50009 Zaragoza, Spain

**Keywords:** alkanes, carbocations, DFT, oxidations, ruthenium tetroxide

## Abstract

The ruthenium tetroxide-mediated oxidation of cyclopentane, tetrahydrofuran, tetrahydrothiophene and *N*-substituted pyrrolidines has been studied computationally by DFT and topological (analysis of the electron localization function, ELF) methods. In agreement with experimental observations and previous DFT calculations, the rate-limiting step of the reaction takes place through a highly asynchronous (3 + 2) concerted cycloaddition through a single transition structure (one kinetic step). The ELF analysis identifies the reaction as a typical one-step-two-stages process and corroborates the existence of a transient carbocation. In the case of pyrrolidines, the carbocation is completely stabilized as an energy minimum in the form of an iminium ion and the reaction takes place in two steps.

## Introduction

**Scheme 1:**
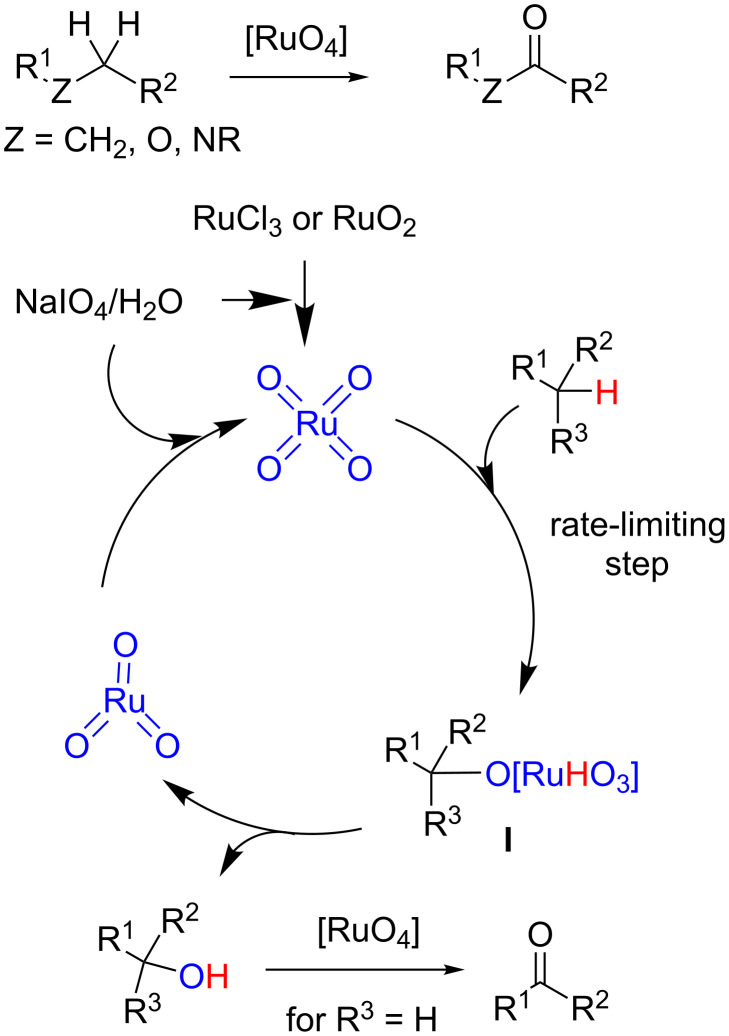
Oxidation of alkanes with RuO_4_.

Ruthenium-catalyzed oxidations [1–2] and, in particular, those involving ruthenium tetroxide [3–4] occupy a privileged position among the modern oxidation methods due to their versatility regarding functional groups that can be oxidized and formed [5]. Alkane functionalization continues to be a current challenge in organic synthesis [6] and oxidation with ruthenium tetroxide allows to introduce an oxygenated functionality (alcohol or carbonyl) into a saturated carbon skeleton [7]. Moreover, if oxygen or nitrogen atoms are present, the reaction leads to the formation of esters [8–9] or amides [10–11], respectively (Scheme 1). The reaction is typically performed by preparing ruthenium tetroxide in situ from ruthenium species in lower oxidation states (RuCl_3_ or RuO_2_) and an oxidant such as NaIO_4_ [8]. Under these conditions RuO_4_ reacts with the alkane to form intermediate species **I** that evolves to the alcohol and RuO_3_, which is re-oxidized to re-start the catalytic cycle (Scheme 1) [12]. Depending on the substrates and reaction conditions (re-oxidant, solvent, temperature) the alcohol can be oxidized to the corresponding carbonyl derivative [13–14].

**Scheme 2:**
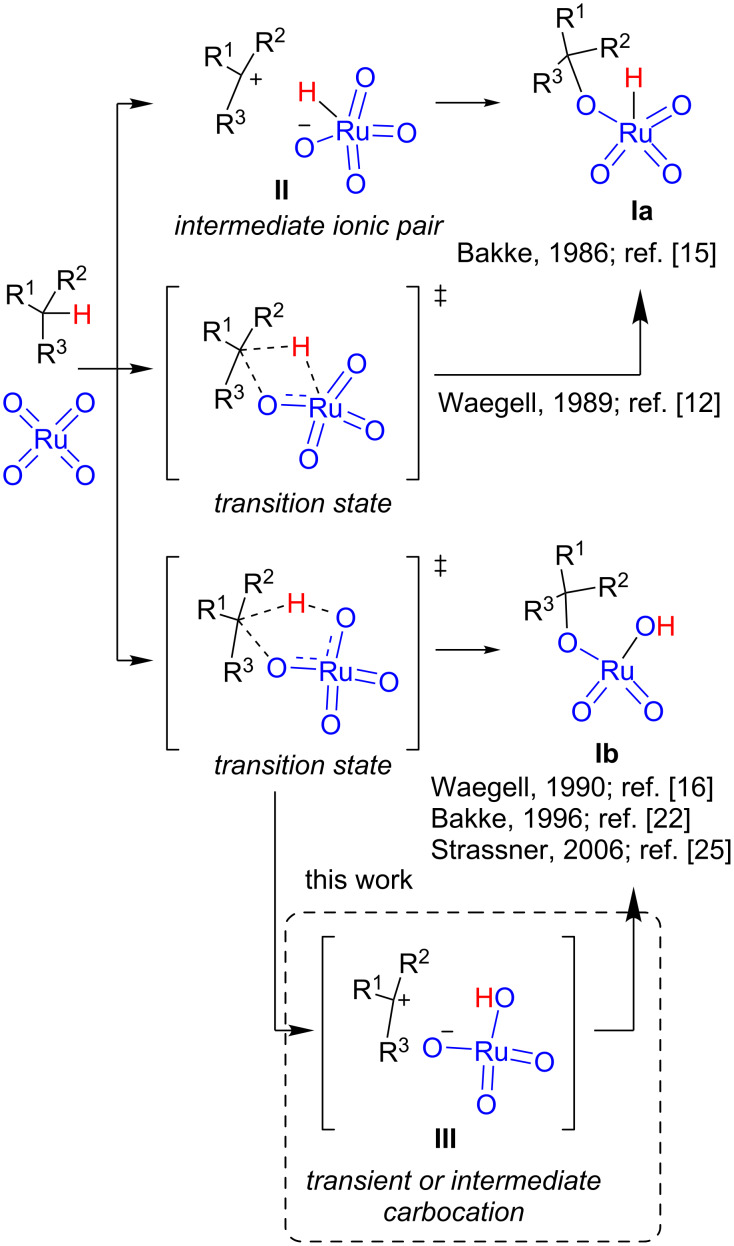
Mechanisms for RuO_4_ oxidation of alkanes.

The rate-limiting step of the catalytic cycle illustrated in Scheme 1 is the initial reaction between RuO_4_ and the alkane, and it has been studied both experimentally and computationally having some initial controversy. The first studies were reported by Bakke et al*.* in 1986 who suggested the formation of intermediate ionic species on the basis of kinetic isotopic effects and solvent and substituents effects (Scheme 2) [15]. Three years later, Waegell et al. proposed a (2 + 2) concerted mechanism [12], although the intimate nature of the organometallic intermediates was not completely elucidated [16]. After some discussion in which Bakke et al. confirmed their initial proposal [17–18] and Waegell et al. proposed a new (3 + 2) asynchronous concerted mechanism [19–20], both groups converged to the latter proposed mechanism when Bakke et al. changed the interpretation of their kinetic isotopic experiments [21–24].

The (3 + 2) concerted mechanism was further confirmed by DFT calculations [25] which were also in agreement with the earlier experiments of Bakke et al. [15]. The computational study also confirmed the hydroxide adduct **Ib** as the active intermediate formed in the reaction. However, Petride et al. have demonstrated that iminium cations are intermediates in the RuO_4_-mediated oxidation of tertiary amines [26] by trapping them with cyanide anion [27–28]. These results point out the formation of transient carbocations **III** that can be stabilized by the presence of heteroatoms in the alpha position. The formation of transient carbocations do not contradict, necessarily, the proposed asynchronous concerted mechanism. A deeper analysis of the full path of the reaction using MD calculations [29] would be needed in order to assess *the synchronicity* and life time of transient species [30]. The recent use of MD simulations has demonstrated that a single transition state can lead to different products in a ratio that depends on reaction dynamics [31–33]. The study of molecular dynamics trajectories has allowed characterization of ambimodal transition states in reactions involving carbocations [34–35].

We have demonstrated computationally the presence of transient carbocations in reactions taking place in one kinetic step including asynchronous concerted cycloadditions [36] and SN_2_-type reactions [37]. Moreover, the real existence of transient carbocations – which are not energy minima – predicted computationally has also been recently proven experimentally in a reaction with an only transition state in which a planar transient species is developed during the reaction [38]. The formation of transient carbocations developed along the reaction course cannot be detected by the calculation of stationary points alone. The use of topological methods, in particular the analysis of the electron localization function (ELF) [39–40] is an excellent approach to evaluate the synchronicity of organic reactions [41–42] and consequently, to predict the formation of transient carbocations [43].

**Scheme 3:**
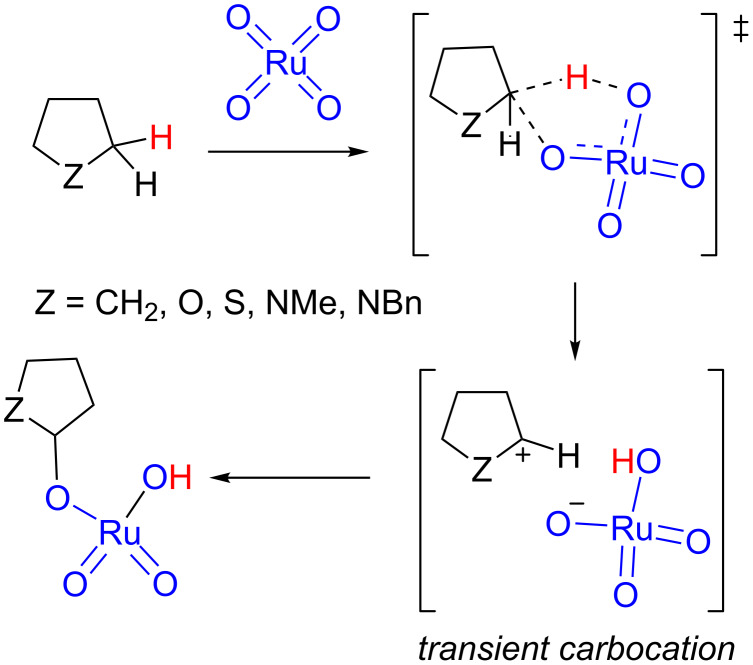
Oxidation of saturated five-membered (hetero)cyclic compounds.

In this work, we report a computational study of the RuO_4_-mediated oxidation of cyclopentane, tetrahydrofuran, tetrahydrothiophene, and *N*-methyl- and *N-*benzylpyrrolidine to evaluate the extension in which transient carbocations can be formed (and whether they can become energy minima) during the rate-limiting step (Scheme 3). The RuO_4_ oxidation of cyclopentane [44] and tetrahydrofuran [45] have been experimentally reported as well as the oxidation of *N*-acylpyrrolidines to the corresponding lactams [46]. Admittedly, the oxidation of tetrahydrothiophene has been approached only computationally since in that case the sulfur atom would be more easily oxidized. Since the general mechanism consisting of a (3 + 2) transition state has been confirmed as the preferred one [25], we restricted the study to this approach.

## Computational Methods

The procedures are analogous to those previously reported [43]. All of the calculations were performed using the Gaussian 09 program [47]. Computations were done using the B3LYP functional [48–49] in conjunction with Grimme’s dispersion correction [50–51] (henceforth referred to as B3LYP-d3bj). The standard basis set Def2SVP was employed [52–53]. For the purpose of comparison optimizations at gas phase and considering solvent effects (both acetonitrile and water, CPCM [54–55]) were carried out. The optimizations were carried out using the Berny analytical gradient optimization method [56]. Minimum energy pathways for the reactions studied were found by the corresponding IRC analysis [57], using the Hratchian–Schlegel algorithm [58]. The individual reactions involved in the study are bimolecular processes. In order to avoid errors due to entropic effects when comparing all stationery points in an only energy diagram, a correction to free energy was made by substracting Strans contribution and considering a 1 M concentration [59]. Single point calculations at the 3ξ level of theory, using the Def2TZVP basis set and considering solvent effects, were carried out over optimized geometries to obtain more accurate energy values. The electronic structures of stationary points were analyzed by the topological analysis of the gradient field of electron localization function (ELF) [39–40,60–66]. The ELF study was performed with the TopMod program [67] using the corresponding monodeterminantal wavefunctions of all the structures of the IRC. Structural representations were generated using CYLView [68]. The models used for calculations are those indicated in Scheme 3.

## Results and Discussion

**Scheme 4:**
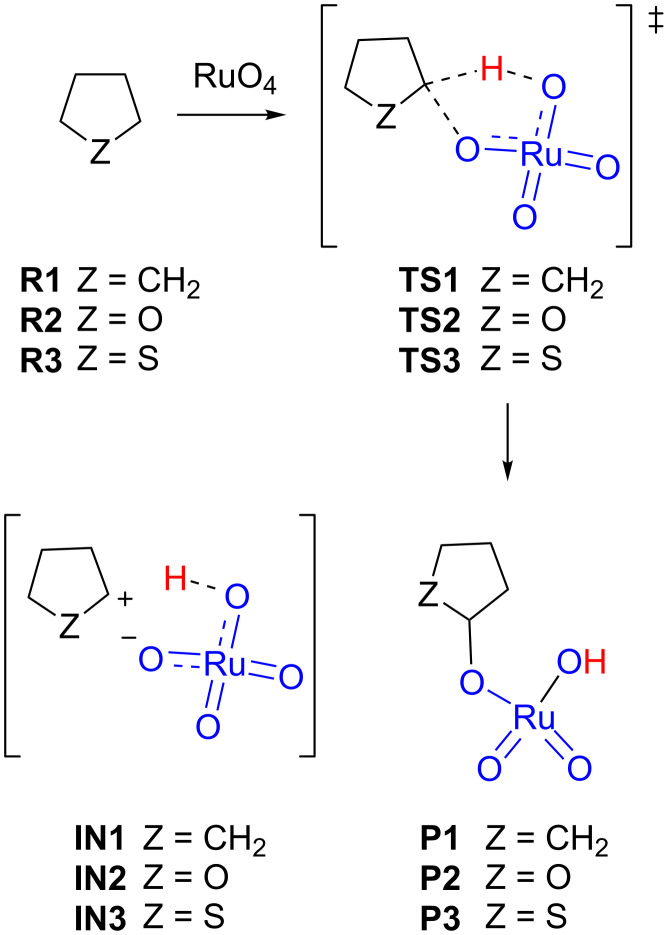
Rate-limiting step for the oxidation of cyclopentane (**R1**), tetrahydrofuran (**R2**) and tetrahydrothiophene (**R3**).

We first studied the oxidations of cyclopentane (**R1**), tetrahydrofuran (**R2**) and tetrahydrothiophene (**R3**, Scheme 4). The geometries of all stationary points were optimized at the B3LYP-d3bj/Def2SVP level of theory in the gas phase and considering solvent effects for acetonitrile and water and their corresponding energy values were calculated at the same level. Since the experimental conditions for the oxidation reactions usually involve a polar medium containing water, all discussions were based on data obtained considering solvent effects for water (for the results using other levels of theory see Supporting Information File 1). We located the corresponding transition structures **TS1**, **TS2** and **TS3**. Any attempt to locate (and optimize) ionic pairs **IN1**, **IN2** and **IN3** failed and, in all cases, the optimization ends at the corresponding products **P1**–**3**, clearly indicating that those ionic pairs are not stable as energy minima even in highly polar conditions (modelled using continuum water solvent).

The obtained energy barriers were 14.6, 6.0 and 7.5 kcal/mol for **TS1**, **TS2** and **TS3**, respectively, predicting an easier oxidation for the heterocyclic compounds. Similar differences between the barriers were obtained in acetonitrile (barriers of 16.6, 8.0 and 8.8 kcal/mol for **TS1**, **TS2** and **TS3**, respectively); the highest observed barriers with respect to water are in agreement with a highly polar reaction.

**Figure 1:**
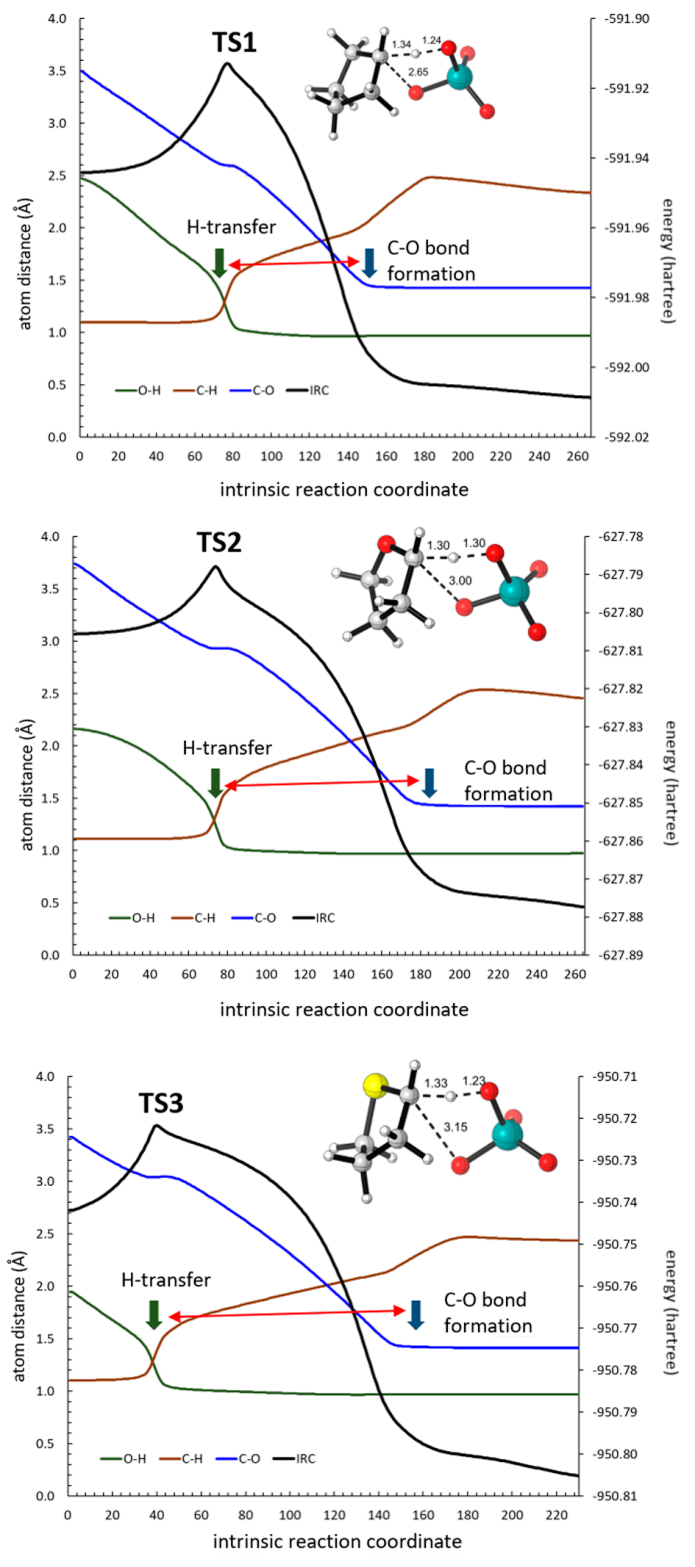
Optimized (B3LYP-d3bj/Def2SVP/cpcm=MeCN) geometries of transition structures corresponding to the oxidation of cyclopentane (**TS1**), tetrahydrofuran (**TS2**) and tetrahydrothiophene (**TS3**). The IRC (black trace) and O–H (green trace), C–H (brown trace), and C–O (blue trace) distances are also given. The double red arrow indicates the delay between H transfer and C–O bond formation.

The corresponding transition structure for cyclopentane **TS1** showed a typical geometry for an asynchronous concerted reaction (Figure 1) in agreement with that observed in the previous study carried out at the B3LYP/6-31G(d) level of theory with implicit MeCN solvent [25]. In that study, the forming/breaking bond distances (estimated for decalines in acetonitrile and acetone) were in the following ranges: the C–H bonds were 1.37–1.41 Å, the O–H bonds were 1.19–1.22 Å, and the C–O bonds were 2.57–2.84 Å. The observed values for **TS1** in water (C–H: 1.34 Å; O–H: 1.24 Å and C–O: 2.65 Å) and acetonitrile (C–H: 1.34 Å; O–H: 1.24 Å and C–O: 2.64 Å) were similar, but placing the hydrogen atom slightly closer to the carbon atom. Similar distances for the C–H–O system were found for **TS2** (C–H: 1.30 Å and O–H: 1.30 Å) and **TS3** (C–H: 1.33 Å and O–H: 1.23 Å), corresponding to tetrahydrofuran and tetrahydrothiophene, respectively. On the other hand, the C–O distance increased to 3.00 Å in **TS2** and to 3.15 Å in **TS3** (similar data were found in acetonitrile, see Supporting Information File 1) clearly indicating a delay in the formation of the C–O bond. This situation is compatible with the stabilization of a developing positive charge at the carbon atom by a mesomeric effect of the α-heteroatom. Nevertheless, the corresponding IRCs for the three transition structures confirmed a concerted reaction connecting the corresponding encounter pairs **EP1**, **EP2** and **EP3** (see Supporting Information File 1), formed from reagents **R1**–**3** and ruthenium(IV) tetroxide, with **P1**, **P2** and **P3**, respectively. A close inspection of the IRCs revealed a shoulder characteristic of a transient carbocation [23] which is more pronounced following the sequence **R1** < **R2** < **R3**. The preliminary analysis of the evolution of bonds along those IRCs further confirmed a high asynchronicity, showing a substantial delay in the formation of the C–O bond with respect to the H transfer from the C atom to the O atom, and following the sequence **TS1** < **TS2** < **TS3** (Figure 1, red arrows).

Even though the above data clearly point out to a typical one-step-two-stage process [69–71] in which the bonds are broken and formed in two separate events, only a topological analysis of the ELF will provide the exact moment in which those events take place and provide evidences of the formation of a transient carbocation. The ELF analysis [39–40,72–73] allows calculation of the so-called basins of attractors [74], that are the areas in which the probability of finding an electron pair is maximal. Monosynaptic and disynaptic basins correspond to separate atoms and bonds, respectively. When a bond is formed, two monosynaptic basins merge into a new disynaptic basin.

The complete ELF analyses of the IRCs corresponding to **TS1**, **TS2** and **TS3** have allowed identifying changes in the electron distribution of atoms and bonds during the reaction coordinate and the precise moment in which bonds are broken and formed.

**Figure 2:**
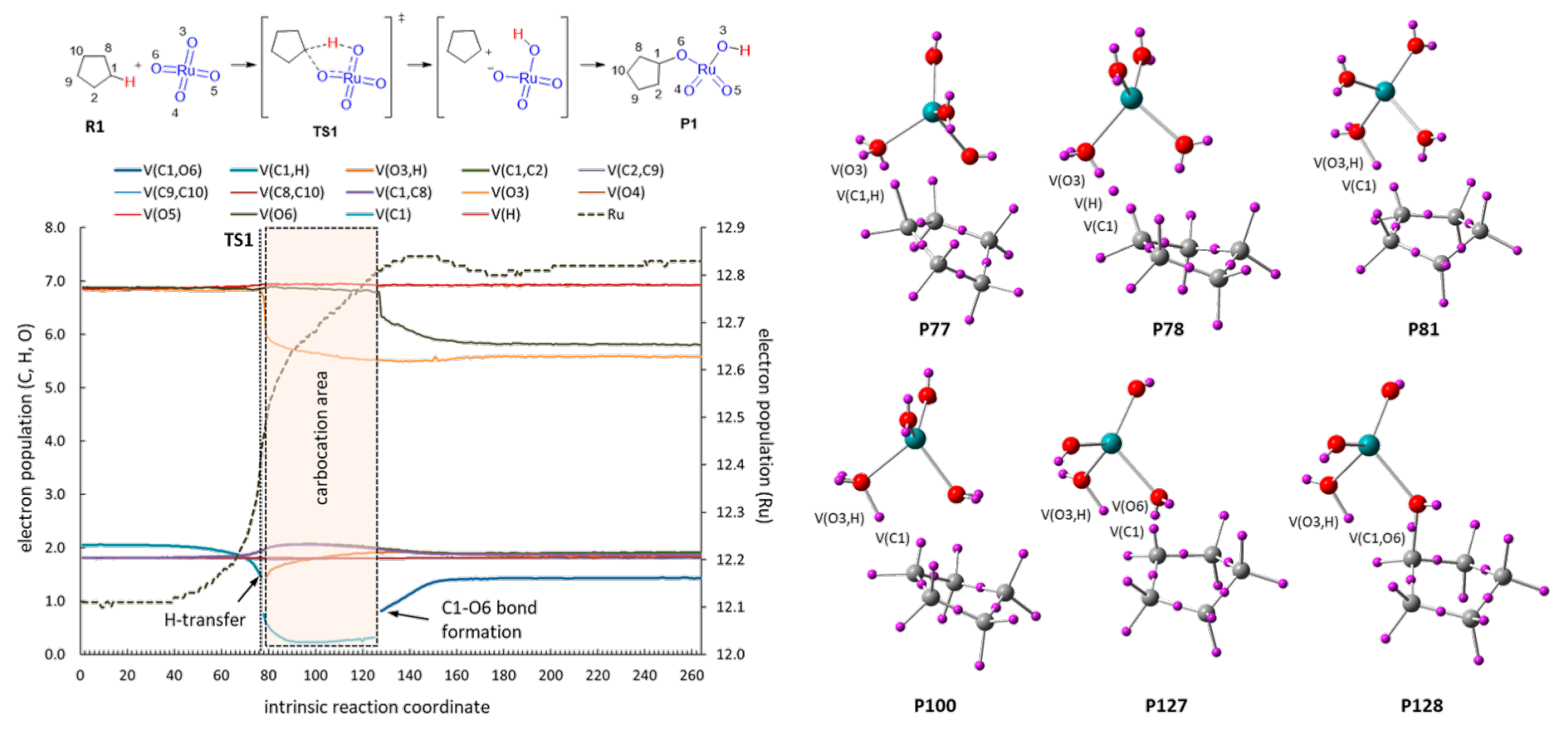
ELF analysis for the oxidation of cyclopentane (**R1**). Left: evolution of the electron population along the IRC. Right: Descriptors of basins at selected points of the IRC.

The ELF analysis of the oxidation of cyclopentane (Figure 2) showed an asynchronous concerted process with the transition state at point 77 (29% of IRC). Breaking of the C1–H bond is immediately followed by H transfer (point 78) and O3–H bond formation (point 81). The formation of the second C–O bond takes place at point 128 (48% of IRC). The gap between H transfer and C1–O6 bond formation (from point 81 to point 127, corresponding to 17% of IRC) is compatible with the existence of a transient carbocation at C1. Nevertheless, the reaction might also be considered just an asynchronous concerted process with a clear partial charge development during the formation of O3–H and C1–O6 bonds that takes place in two separate events.

**Figure 3:**
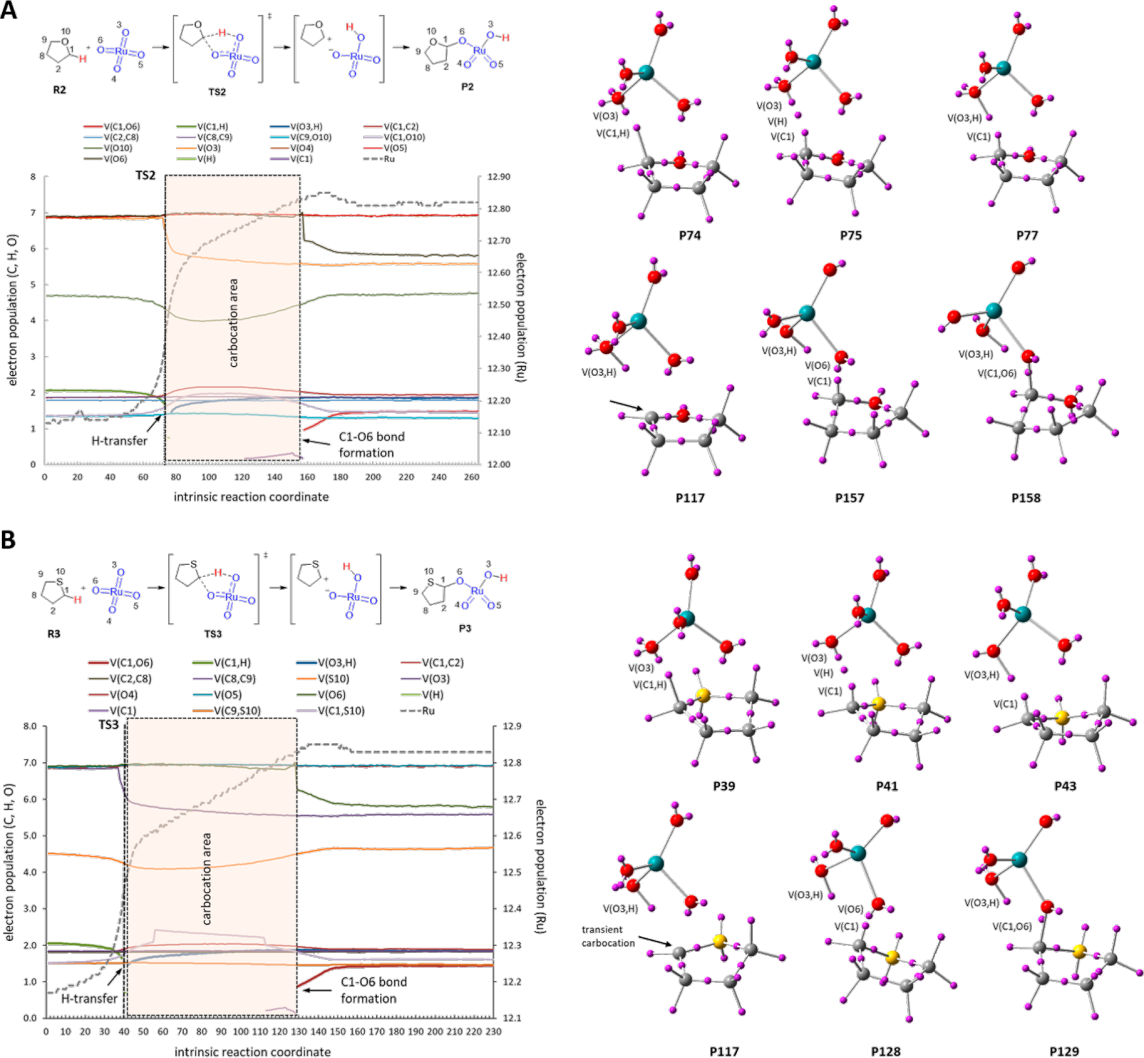
ELF analysis for the oxidation of tetrahydrofuran (**R2**, A) and tetrahydrothiophene (**R3**, B). Left: evolution of the electron population along the IRC. Right: Descriptors of basins at selected points of the IRC.

The stabilization of the above-mentioned transient carbocation can be achieved by introducing heteroatoms. The ELF analysis corresponding to the oxidation of tetrahydrofuran (Figure 3A) again showed a typical one-step-two-stage situation. In this case, the gap between the H transfer and the formation of C1–O6 bond (from point 77 to point 157, corresponding to a 30% of the IRC) is larger than that of cyclopentane (corresponding to a 17% of IRC) as a consequence of the stabilizing effect of the incipient positive charge exerted by the oxygen atom. The effective existence of a transient carbocation is supported by the disappearance of V(C1) and the trigonal planar geometry observed for C1 in the above indicated gap. The evolution of the electron population is in clear agreement with the development of a partial positive charge at C1 (+0.25 at point 100). The oxidation of tetrahydrothiophene reflects the same situation, but to a greater degree (Figure 3B).

**Figure 4:**
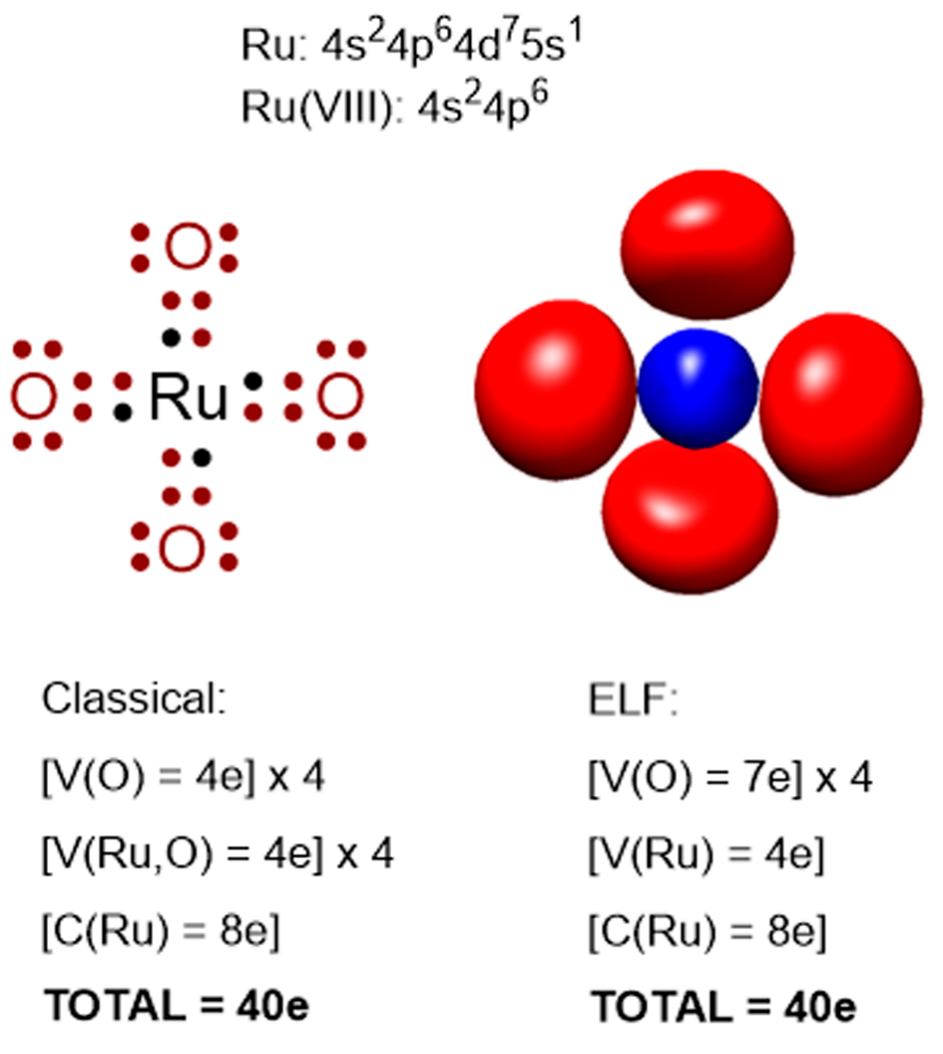
ELF assignment of electrons to the Ru environment. C(Ru) corresponds to a monosynaptic core basin assigned to ruthenium. V(Ru) and V(O) correspond to monosynaptic valence basins assigned to ruthenium and oxygen atoms, respectively. V(Ru,O) corresponds to a disynaptic valence basin assigned to a Ru–O bond (not present in ELF analysis).

Interestingly, the ELF analyses evidence the high polarity of Ru–O bonds by assigning about 7e to the oxygen atoms. Because of this, during the reaction coordinate an increase of only 1e is assigned to Ru for which about 12e (coming from 4e of valence directly assigned plus 8e from the last layer 4s^2^4p^6^) have been initially assigned (Figure 4). Although this assignment does not correlate with the classical valence concept of 8e for Ru(VIII) it actually reflects a more real situation.

As stated above, attempts of locating the corresponding ion pairs failed, ending at the final **P1**–**3** products and confirming that they are not stationary points. However, this does not mean that they cannot exist in the form of transient species as we have recently demonstrated [38].

**Scheme 5:**
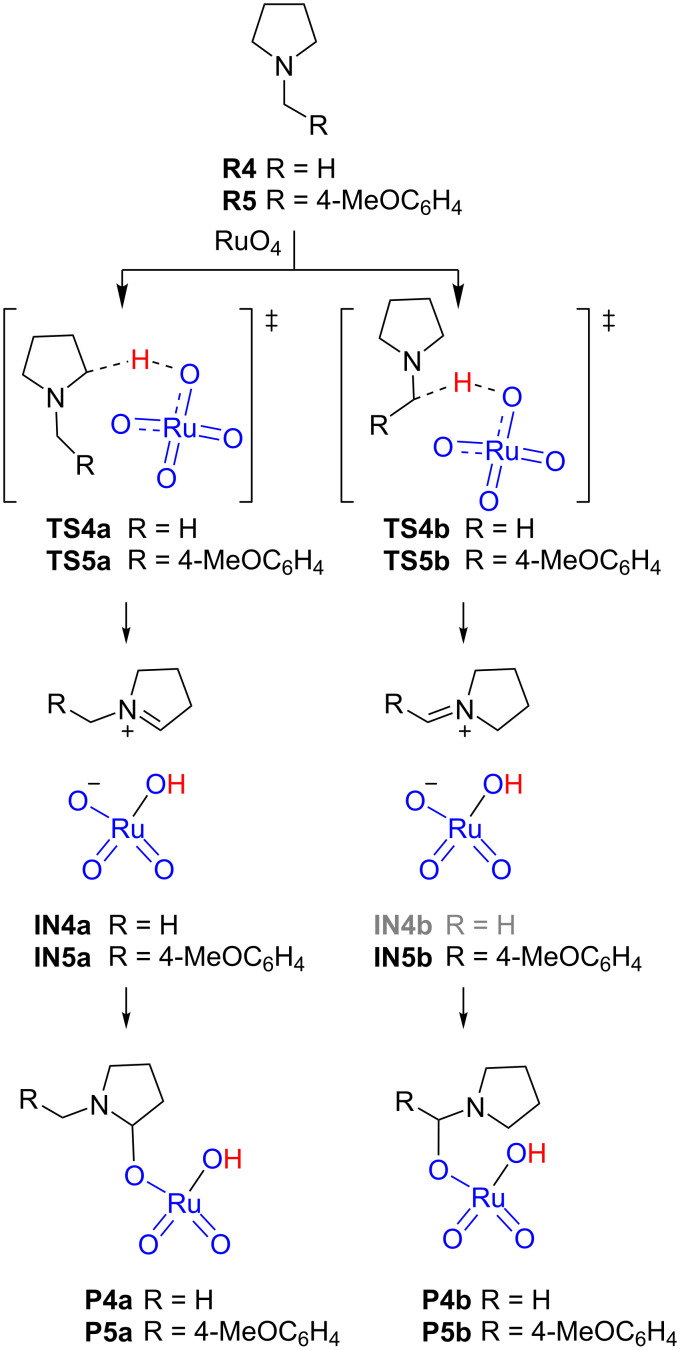
Rate-limiting step for the oxidation of *N*-methyl- and *N*-benzylpyrrolidines **R4** and **R5**, respectively.

A completely different situation was found with the oxidation of *N*-methylpyrrolidine (**R4**) and *N*-benzylpyrrolidine **R5** (Scheme 5). In the case of *N*-alkylpyrrolidines two regiosiomeric oxidations can take place at *endo* (cycle) and *exo* (*N-*chain) positions. We located the four transition structures **TS4a** and **TS5a**, corresponding to the *endo* series, and **TS4b** and **TS5b**, corresponding to the *exo* series.

**Figure 5:**
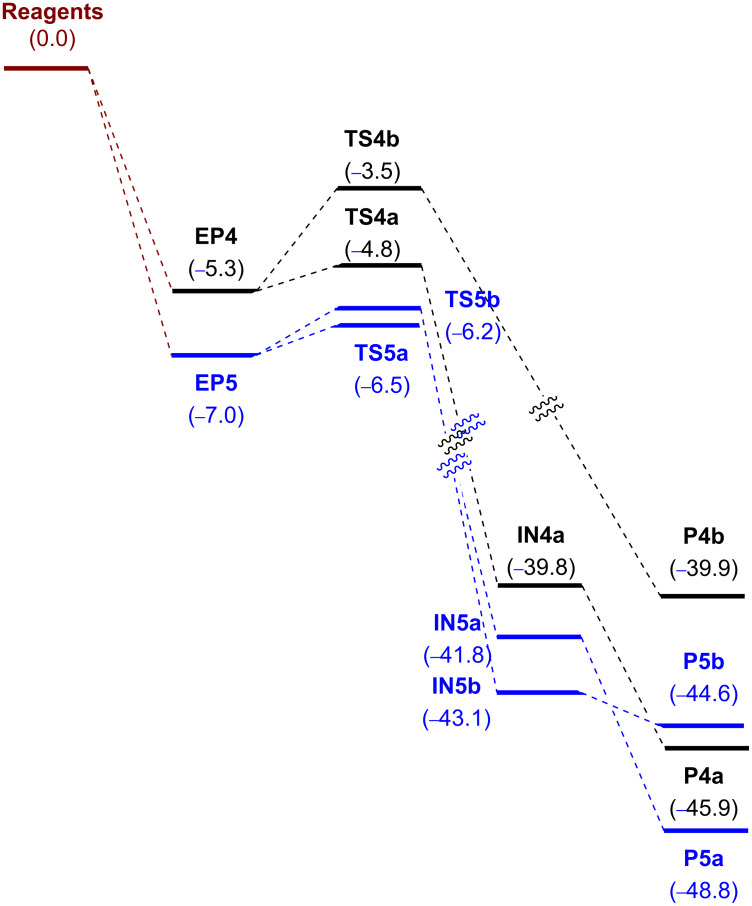
Energy profile for the oxidation of **R4** and **R5**. Relative energies, calculated at the B3LYP-d3bj/Def2TZVP/cpcm=water level of theory, are given in kcal/mol.

In all cases, the observed barriers were below the reagents illustrating a favorable reaction (see Figure 5). For *N*-methylpyrrolidine (**R4**), the *endo* oxidation was preferred over the *exo* oxidation by 1.3 kcal/mol whereas for *N*-benzylpyrrolidine **R5** the difference in favor of the *endo* oxidation was only 0.3 kcal/mol suggesting a directing effect of the *p*-methoxyphenyl group. Notably, the IRC analyses of the transition structures revealed as end points of the reactions the ion pairs **IN4**,**5**. Indeed, optimization of those points led to **IN4a**, **IN5a** and **IN5b** as energy minima; only **IN4b** could not be located, the optimization of which led to **P4b**. Transformation of ion pairs into the corresponding products **P4a** and **P5a**,**b** was found to be essentially barrierless. As expected, the ion pairs identified as minima adopt the form of an iminium ion, the most stable being **IN5b**, corresponding to that conjugated with the *p*-methoxyphenyl group, which stabilizes the positive charge. These results are in agreement with the experimental findings of Petride and co-workers, who demonstrated the existence of iminium ions as intermediates in this sort of oxidation [26].

**Figure 6:**
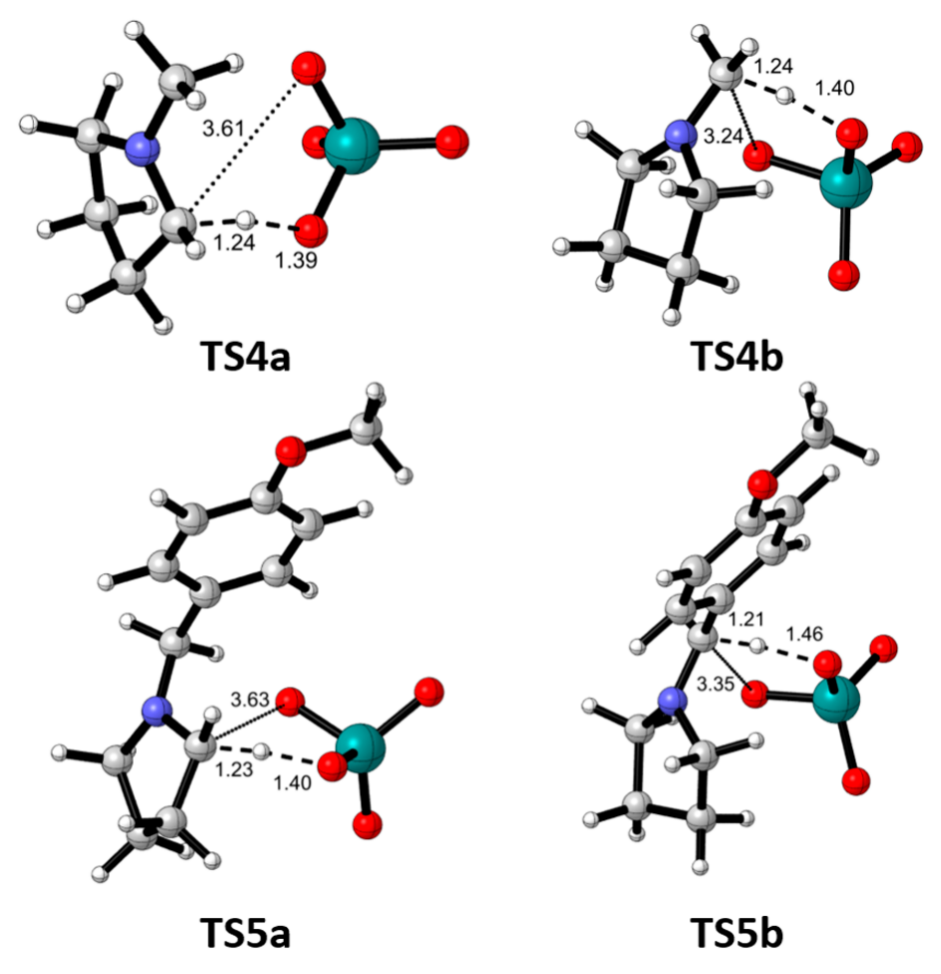
Optimized (B3LYP-d3bj/Def2SVP/cpcm=water) transition structures for the oxidation of **R4** and **R5**.

The geometries of the transition structures showed large distances between the carbon to be oxidized and the ruthenium oxygen indicating that, in fact, they do not correspond to forming bonds (Figure 6). The largest distances correspond to the formation of *endo* iminium ions (3.61 Å and 3.63 Å for **TS4a** and **TS5a**, respectively). The shortest distance (3.24 Å) was observed for **TS4b** in agreement with the direct formation of **P4b** as mentioned above.

We performed the ELF analysis for the *endo* oxidation reaction of **R4** (see Supporting Information File 1) and, as expected, we only observed the H transfer corresponding to the concomitant breaking of the C–H bond and formation of the O–H bond but the resulting iminium ion was stable enough to be considered a real intermediate according to IUPAC definition of 1994, which is: “*A molecular entity with a lifetime appreciably longer than a molecular vibration – ****corresponding to a local potential energy minimum**** of depth greater than R T – that is formed (directly or indirectly) from the reactants and reacts further to give (either directly or indirectly) the products of a chemical reaction*” [75].

Although, according to the IUPAC definition a transient carbocation cannot be considered an intermediate (since it is required to be a local energy minimum), this transient carbocation does in fact exist, as we have demonstrated experimentally in a reaction in which the chiral information is lost as a consequence of the presence of a transient carbocation [38]. The stability of the transient carbocation can be enhanced by the presence of heteroatoms that stabilize the developing positive charge by resonance, as in the case of tetrahydrofuran and tetrahydrothiophene. Moreover, the presence of a nitrogen atom provides enough stabilization to be located as an energy minimum and to be captured experimentally [26]. Table 1 summarizes the differences observed in the studied cases.

**Table 1:** Summary of results.

	barrier^a^	H transfer^b^	C–O formation^b^	% carbocation^c^

**R1**	14.6	30	49	17
**R2**	6.0	29	60	30
**R3**	7.5	17	56	40
**R4**	−3.5^d^	20	–^e^	80

^a^Given in kcal/mol relative to separate reagents. ^b^Given in % with respect to the total number of points of the IRC. ^c^Calculated on the number of points between the H transfer and C–O bond formation with respect to the total number of points of the IRC. ^d^The corresponding encounter pair is 8.5 kcal/mol below the reagents. ^e^The product of the reaction is the iminium cation therefore the C–O bond is not formed.

The presence of a heteroatom contributes to lower the energy barrier of the oxidation reaction, and in the case of the pyrrolidine, it is below the reactant, demonstrating the stabilizing effect of the heteroatom in the transition structure. The asynchronicity of the reaction can be measured on the basis of the lapse between breaking of the C–H bond and formation of the C–O bond. Whereas H transfer takes places at similar moments (30% and 29% of the IRC, for the representative cases of cyclopentane (**R1**) and tetrahydrofuran (**R2**), respectively) after starting the reaction, the formation of the C–O bond takes more time for **R2** (60% of the IRC) than for **R1** (49% of the IRC), giving more chance to the transient carbocation for the former (30% of the IRC vs 17% of the IRC for the latter).

## Conclusion

The oxidation of cyclopentane with ruthenium tetroxide is a highly polar asynchronous concerted process that during a brief lapse of time develops a transient carbocation. This result does not contradict previous calculations [25], but does point out the necessity of analyzing the full reaction coordinate to detect species that might explain some chemical behavior. Indeed, further theoretical studies on MD simulations would be needed to elucidate the lifetime of the transient carbocation [34–35]. These results demonstrate the one-step-two-stage character [42] of the ruthenium oxidations of alkanes in which H transfer and O–C bond formation take place in two separate events within the same reaction coordinate. We suggest a more adequate use of the IUPAC definition of intermediate given in 1996 [76] (*any reaction species that is neither an initial reactant nor a final product is referred to as an intermediate*) rather than that of 1994 [75], since it is in this case not strictly necessary for the transient carbocations described above to be local energy minima.

## Supporting Information

File 1 Energy data, optimized geometries, full data of ELF analysis and Cartesian coordinates.
