# Supplementary material for: Transient and intermediate carbocations in ruthenium tetroxide oxidation of saturated rings
Source: Beilstein J Org Chem. 2019 Jul 11;15:1552–62. doi: 10.3762/bjoc.15.158 (PMC6633598; doi:10.3762/bjoc.15.158)
Supplement: File 1 — Energy data, optimized geometries, full data of ELF analysis and Cartesian coordinates. [file Beilstein_J_Org_Chem-15-1552-s001.pdf]

BEILSTEIN JOURNAL OF ORGANIC CHEMISTRY

Supporting Information

for

Transient and intermediate carbocations in ruthenium tetroxide oxidation of saturated rings

Manuel Pedrón, Laura Legnani, Maria-Assunta Chiacchio, Pierluigi Caramella, Tomás Tejero and Pedro Merino

Beilstein J. Org. Chem. **2019,** *15,* 1552–1562. doi:10.3762/bjoc.15.158

Energy data, optimized geometries, full data of ELF analysis and Cartesian coordinates

License and Terms: This is a supporting information file under the terms of the Creative Commons Attribution License (http://creativecommons.org/licenses/by/4.0). Please note that the reuse, redistribution and reproduction in particular requires that the authors and source are credited.
The license is subject to the *Beilstein Journal of Organic Chemistry* terms and conditions: (https://www.beilstein-journals.org/bjoc)

Table of contents

Absolute and relative energies........ S2

Optimized geometries of transition structures........ S5

ELF analyses........ S7

Cartesian coordinates........ S40

Absolute and relative energies

Table S1. Calculated (b3lyp-gd3bj/def2tzvp(cpcm=water//b3lyp-gd3bj//def2svp/cpcm=water) absolute (hartree) and relative (kcal/mol) free energies for the reactants, transition structures and products corresponding to the oxidation of **R1.**

	E(0)	ΔE(0)	G	ΔG	im. freq
**RuO4**	-395.903443		-395.930584		
**R1**	-196.510159		-196.537248		
**Reagents**	-592.413602	0.0	-592.467832	0.0	
**EP1**	-592.417526	-2.5	-592.458056	-2.6	
**TS1**	-592.390308	14.6	-592.427942	14.6	-1281.0
**P1**	-592.478365	-40.6	-592.516452	-40.6	

Table S2. Calculated (b3lyp-gd3bj/def2tzvp(cpcm=water//b3lyp-gd3bj//def2svp/cpcm=water) absolute (hartree) and relative (kcal/mol) free energies for the reactants, transition structures and products corresponding to the oxidation of **R2.**

	E(0)	ΔE(0)	G	ΔG	im. freq
**RuO4**	-395.903443		-395.930584		
**R2**	-232.445780		-232.473781		
**Reagents**	-628.349224	0.0	-628.404366	0.0	
**EP2**	-628.352740	-2.2	-628.391449	-2.4	
**TS2**	-628.338725	6.6	-628.379775	6.0	-1232.9
**P2**	-628.427989	-49.4	-628.464953	-49.7	

Table S3. Calculated (b3lyp-gd3bj/def2tzvp(cpcm=water//b3lyp-gd3bj//def2svp/cpcm=water) absolute (hartree) and relative (kcal/mol) free energies for the reactants, transition structures and products corresponding to the oxidation of

	E(0)	ΔE(0)	G	ΔG	im. freq
**RuO4**	-395.903443		-395.930584		
**R3**	-555.436335		-555.464960		
**Reagents**	-951.339779	0.0	-951.395545	0.0	
**EP3**	-951.353117	-8.4	-951.391626	-8.5	
**TS3**	-951.327509	7.7	-951.366306	7.5	-1281.7
**P3**	-951.403375	-39.9	-951.440993	-40.1	

Table S4. Calculated (b3lyp-gd3bj/def2tzvp(cpcm=water//b3lyp-gd3bj//def2svp/cpcm=water) absolute (hartree) and relative (kcal/mol) free energies for the reactants, transition structures and products corresponding to the oxidation of **R4.**

	E(0)	ΔE(0)	G	ΔG	im. freq
**RuO4**	-395.903443		-395.930584		
**R4**	-251.858668		-251.888674		
**Reagents**	-647.762111	0.0	-647.819258	0.0	
**EP4**	-647.770427	-5.2	-647.810129	-5.3	
**TS4a**	-647.769335	-4.5	-647.809217	-4.8	-690.3
**TS4b**	-647.767200	-3.2	-647.806114	-3.5	-681.8
**IN4a**	-647.825706	-39.9	-647.866491	-39.8	
**P4a**	-647.835231	-45.9	-647.874327	-45.9	
**P4b**	-647.825280	-39.6	-647.864819	-39.9	

Table S5. Calculated (b3lyp-gd3bj/def2tzvp(cpcm=water//b3lyp-gd3bj//def2svp/cpcm=water) absolute (hartree) and relative (kcal/mol) free energies for the reactants, transition structures and products corresponding to the oxidation of **R5.**

	E(0)	ΔE(0)	G	ΔG	im. freq
**RuO4**	-395.903443		-395.930584		
**R5**	-597.481249		-597.522439		
**Reagents**	-993.384693	0.0	-993.453024	0.0	
**EP5**	-993.395516	-6.8	-993.447428	-7.0	
**TS5a**	-993.394954	-6.4	-993.444091	-6.5	-589.9
**TS5b**	-993.394416	-6.1	-993.443282	-6.2	-412.1
**IN5a**	-993.451744	-42.1	-993.501946	-41.8	
**IN5b**	-993.453456	-43.1	-993.504582	-43.1	
**P5a**	-993.462561	-48.9	-993.512085	-48.8	
**P5b**	-993.455851	-44.7	-993.504403	-44.6	

Table S6. Calculated (b3lyp-gd3bj/def2tzvp(pcm=MeCN//b3lyp-gd3bj//def2svp/pcm=MeCN) absolute (hartree) and relative (kcal/mol) free energies for the reactants, transition structures and products corresponding to the oxidation of **R1.**

	E(0)	ΔE(0)	G	ΔG	im. freq
**RuO4**	-395.9028375		-395.9299785		
**R1**	-196.5101356		-196.5372236		
**Reagents**	-592.4129732	0.0	-592.4672022	0.0	
**EP1**	-592.4169686	-2.5	-592.4574856	-2.6	
**TS1**	-592.3896939	14.6	-592.4273289	14.6	-1280.9
**P1**	-592.4775578	-40.5	-592.5156378	-40.5	

Table S7. Calculated (b3lyp-gd3bj/def2tzvp(pcm=MeCN//b3lyp-gd3bj//def2svp/pcm=MeCN) absolute (hartree) and relative (kcal/mol) free energies for the reactants, transition structures and products corresponding to the oxidation of **R2.**

	E(0)	ΔE(0)	G	ΔG	im. freq
**RuO4**	-395.9028375		-395.9299785		
**R2**	-232.4456539		-232.4736479		
**Reagents**	-628.3484914	0.0	-628.4036264	0.0	
**EP2**	-628.3519002	-2.1	-628.3904222	-2.3	
**TS2**	-628.3372328	7.1	-628.3747938	6.8	-965.6
**P2**	-628.4271490	-49.4	-628.4640210	-49.6	

Table S8. Calculated (b3lyp-gd3bj/def2tzvp(pcm=MeCN//b3lyp-gd3bj//def2svp/pcm=MeCN) absolute (hartree) and relative (kcal/mol) free energies for the reactants, transition structures and products corresponding to the oxidation of **R3.**

	E(0)	ΔE(0)	G	ΔG	im. freq
**RuO4**	-395.9028375		-395.9299785		
**R3**	-555.4362091		-555.4648351		
**Reagents**	-951.3390466	0.0	-951.3948136	0.0	
**EP3**	-951.3523720	-8.4	-951.3908030	-8.4	
**TS3**	-951.3268517	7.7	-951.3655747	7.5	-1278.6
**P3**	-951.4025749	-39.9	-951.4401499	-40.1	

Table S9. Calculated (b3lyp-gd3bj/def2tzvp(pcm=MeCN//b3lyp-gd3bj//def2svp/pcm=MeCN) absolute (hartree) and relative (kcal/mol) free energies for the reactants, transition structures and products corresponding to the oxidation of **R4.**

	E(0)	ΔE(0)	G	ΔG	im. freq
**RuO4**	-395.9028375		-395.9299785		
**R4**	-251.8586002		-251.8886272		
**Reagents**	-647.7614377	0.0	-647.8186057	0.0	
**EP4**	-647.7697292	-5.2	-647.8093252	-5.2	
**TS4a**	-647.7686556	-4.5	-647.8085136	-4.8	-699.3
**TS4b**	-647.7664943	-3.2	-647.8053883	-3.5	-690.2
**IN4a**	-647.8244123	-39.5	-647.8651843	-39.4	
**P4a**	-647.8344311	-45.8	-647.8735811	-45.8	
**P4b**	-647.8245028	-39.6	-647.8639838	-39.8	

Table S10. Calculated (b3lyp-gd3bj/def2tzvp(pcm=MeCN//b3lyp-gd3bj//def2svp/pcm=MeCN) absolute (hartree) and relative (kcal/mol) free energies for the reactants, transition structures and products corresponding to the oxidation of **R5.**

	E(0)	ΔE(0)	G	ΔG	im. freq
**RuO4**	-395.9028375		-395.9299785		
**R5**	-597.4810172		-597.5222102		
**Reagents**	-993.3838547	0.0	-993.4521887	0.0	
**EP5**	-993.3947838	-6.9	-993.4457558	-7.0	
**TS5a**	-993.3941935	-6.5	-993.4433775	-6.6	-599.1
**TS5b**	-993.3937191	-6.2	-993.4425561	-6.3	-422.8
**IN5a**	-993.4504519	-41.8	-993.5006839	-41.6	
**IN5b**	-993.4515969	-42.5	-993.5026259	-42.4	
**P5a**	-993.4616006	-48.8	-993.5111336	-48.8	
**P5b**	-993.4530474	-43.4	-993.5019974	-43.4	

Optimized geometries of transition structures and intermediates

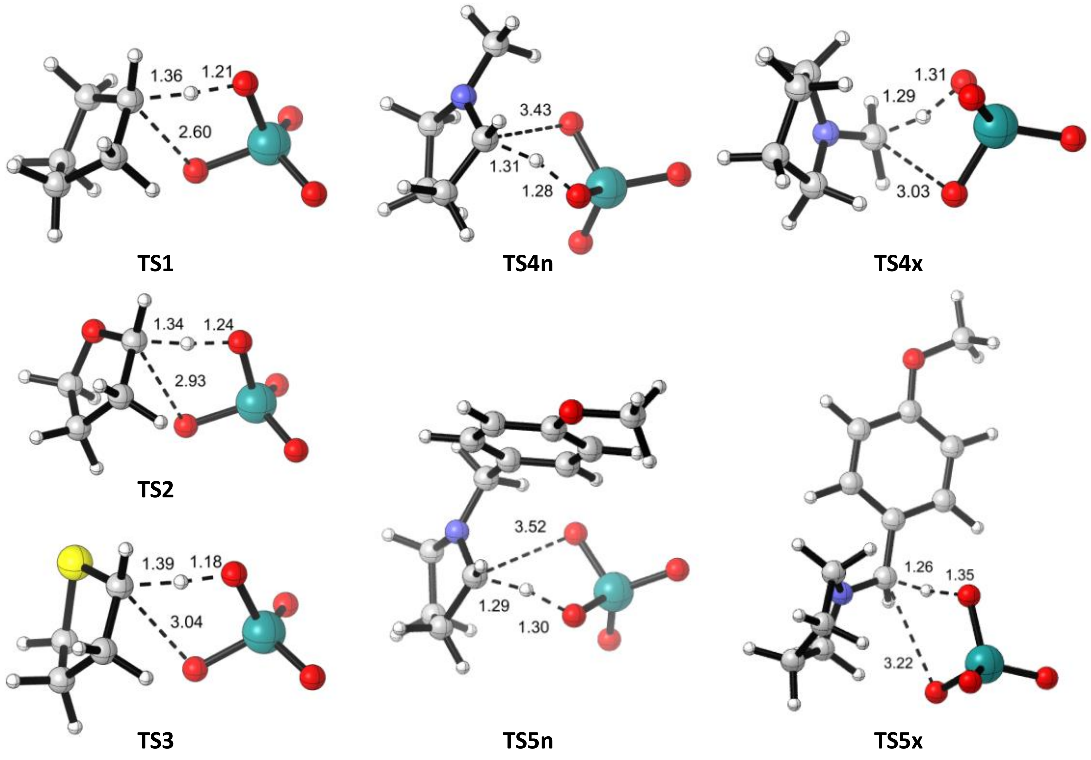

Figure S1. Optimized geometries (b3lyp-gd3bj/def2svp/gas) of transition structures corresponding to the oxidation of **R1-R5**.

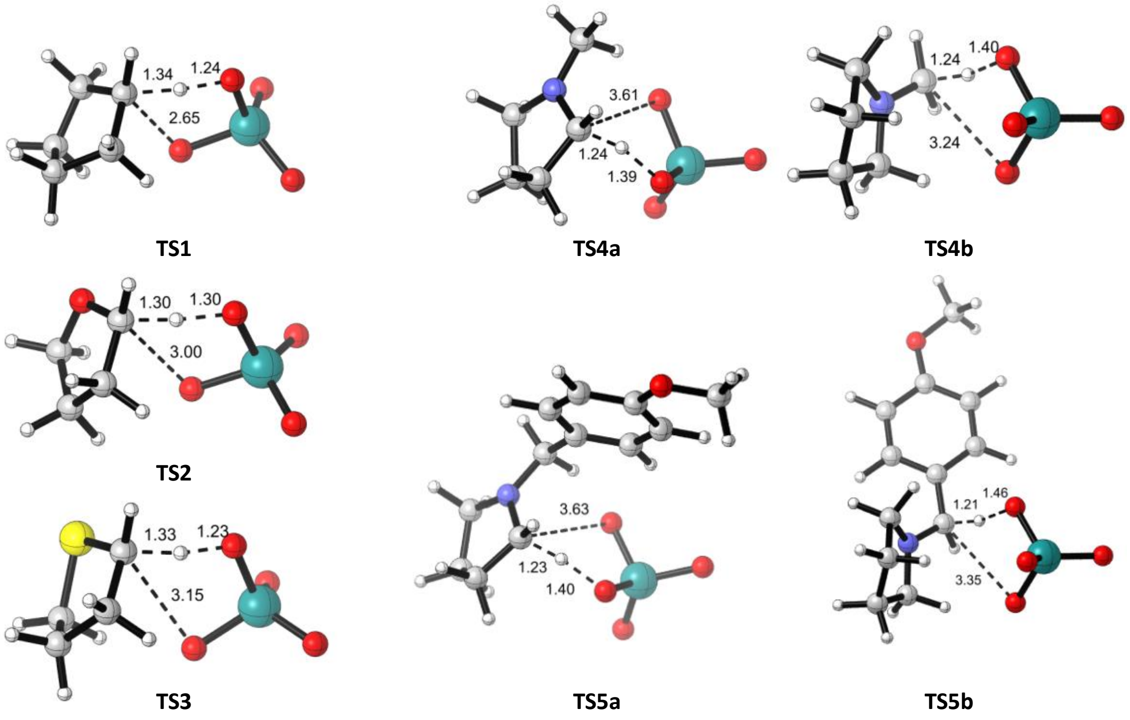

Figure S2. Optimized geometries (b3lyp-gd3bj/def2svp/cpcm=water) of transition structures corresponding to the oxidation of **R1-R5**.

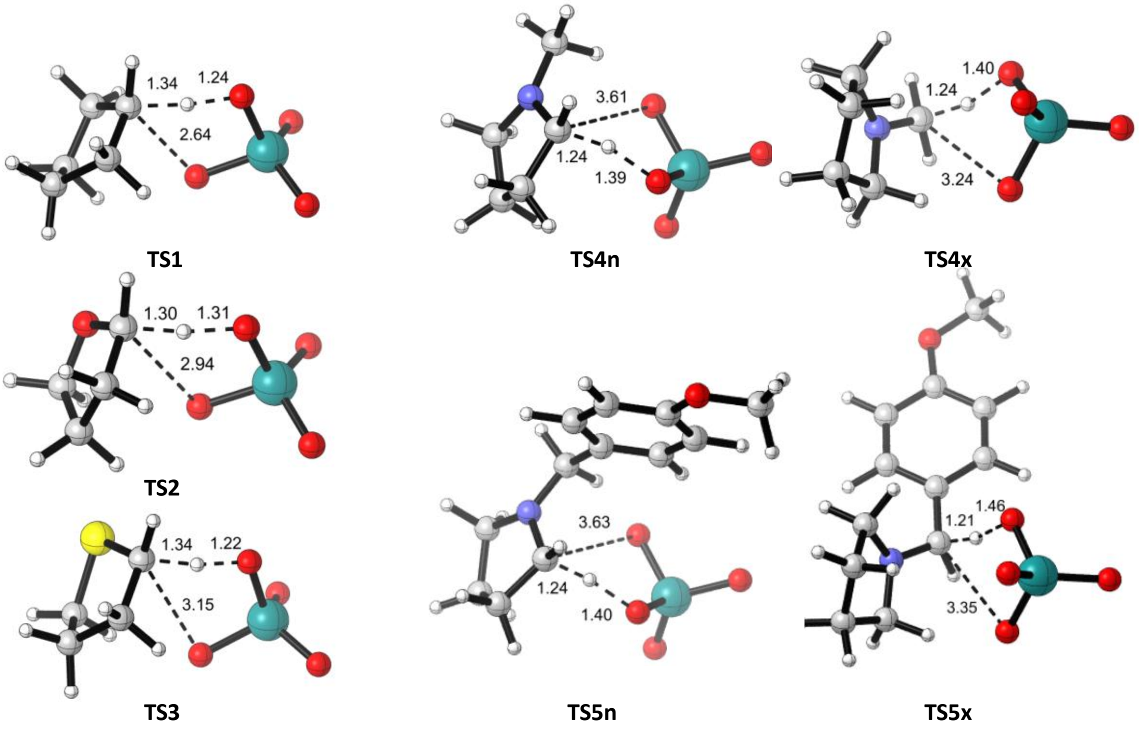

Figure S3. Optimized geometries (b3lyp-gd3bj/def2svp/pcm=MeCN) of transition structures corresponding to the oxidation of **R1-R5**.

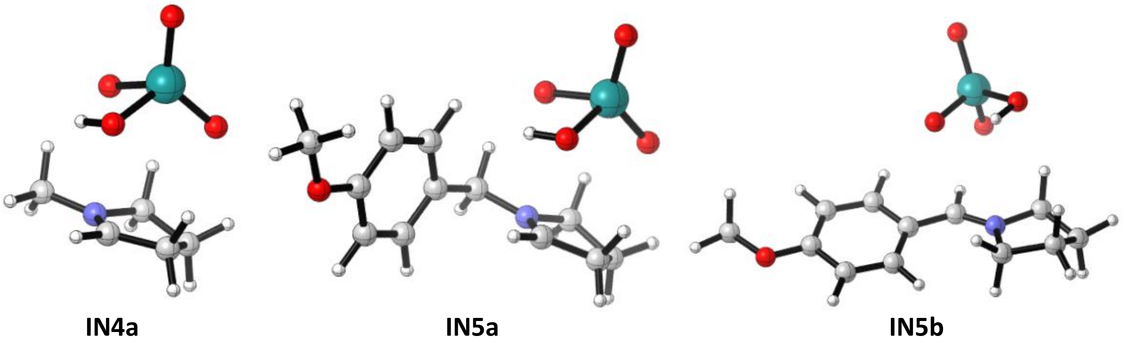

Figure S4. Optimized geometries (b3lyp-gd3bj/def2svp/cpcm=water) of intermediates **IN4a**, **IN5a** and **IN5b**.

ELF analyses

ELF analysis for oxidation of cyclopentane ***R1***

In the oxidation of cyclopentane the H-transfer essentially takes place at the transition state (point 77, 29% of IRC). Breaking of the C1–H bond is concomitant with a decrease of population of O3 (points 77–80), immediately followed by H-transfer (point 78) and O3–H bond formation (point 81) assessed by the appearance of a disynaptic basin V(O3,H). However, some residual density remains at C1 as it keeps a monosynaptic basin V(C1). Indeed, it is only at point 128 (48% of IRC) when V(C1) merges with monosynaptic basin from oxygen V(O6) to disynaptic basin V(C1,O6), indicating the formation of the second C–O bond. The gap between H-transfer and C1–O6 bond formation (from point 81 to point 127) is compatible with the existence of a transient carbocation at C1 as suggested by the slight increase of the electron population of disynaptic basins V(C1,C2) and V(C1,C8). Nevertheless, the continued presence of basin V(C1) evidences the elusive character of such carbocation and the reaction might also be considered just an asynchronous concerted process with a clear charge development during the formation of O3–H and C1–CO6 bonds that takes place in two separated events.

Table S11. ELF basin populations for the oxidation of **R1**

	V1(O3)	V2(O3)	V3(O3)	V1(O4)	V2(O4)	V1(O5)	V2(O5)	V1(O6)	V2(O6)	V3(O6)	V(C1,C2)	V(C1,C8)	V(C8,C10)	V(C9,C10)	V(C2,C9)	V(C1,H)	V(O3,H)	V(C1,O6)	V(C1)	V(H)	Ru
1	6.84	-	-	6.85	-	6.87	-	6.89	-	-	1.81	1.81	1.81	1.81	1.81	2.05	-	-	-	-	12.11
2	6.84	-	-	6.84	-	6.87	-	6.88	-	-	1.81	1.81	1.81	1.81	1.81	2.05	-	-	-	-	12.11
3	6.83	-	-	6.85	-	6.86	-	6.89	-	-	1.81	1.81	1.81	1.81	1.81	2.05	-	-	-	-	12.11
4	6.84	-	-	6.85	-	6.86	-	6.88	-	-	1.81	1.81	1.81	1.81	1.81	2.05	-	-	-	-	12.11
5	6.83	-	-	6.84	-	6.86	-	6.88	-	-	1.81	1.81	1.81	1.81	1.81	2.05	-	-	-	-	12.11
6	6.83	-	-	6.85	-	6.86	-	6.88	-	-	1.81	1.81	1.81	1.81	1.81	2.05	-	-	-	-	12.11
7	6.83	-	-	6.85	-	6.86	-	6.88	-	-	1.80	1.81	1.81	1.81	1.81	2.05	-	-	-	-	12.11
8	6.82	-	-	6.85	-	6.86	-	6.88	-	-	1.81	1.80	1.81	1.81	1.81	2.06	-	-	-	-	12.11

9	6.81	-	-	6.86	-	6.87	-	6.88	-	-	1.81	1.80	1.80	1.81	1.81	2.06	-	-	-	-	12.10
10	6.81	-	-	6.85	-	6.87	-	6.88	-	-	1.81	1.81	1.81	1.81	1.81	2.05	-	-	-	-	12.10
11	6.82	-	-	6.86	-	6.87	-	6.88	-	-	1.81	1.81	1.81	1.81	1.81	2.05	-	-	-	-	12.11
12	6.83	-	-	6.86	-	6.86	-	6.88	-	-	1.81	1.81	1.81	1.81	1.81	2.05	-	-	-	-	12.11
13	6.84	-	-	6.85	-	6.85	-	6.88	-	-	1.81	1.81	1.81	1.81	1.81	2.05	-	-	-	-	12.10
14	6.84	-	-	6.84	-	6.86	-	6.88	-	-	1.81	1.81	1.80	1.81	1.81	2.05	-	-	-	-	12.11
15	6.84	-	-	6.85	-	6.86	-	6.88	-	-	1.81	1.81	1.81	1.81	1.81	2.05	-	-	-	-	12.11
16	6.83	-	-	6.85	-	6.87	-	6.88	-	-	1.81	1.81	1.81	1.81	1.81	2.05	-	-	-	-	12.11
17	6.83	-	-	6.86	-	6.86	-	6.88	-	-	1.81	1.81	1.81	1.81	1.81	2.05	-	-	-	-	12.11
18	6.83	-	-	6.84	-	6.87	-	6.88	-	-	1.81	1.81	1.81	1.81	1.81	2.05	-	-	-	-	12.11
19	6.83	-	-	6.83	-	6.87	-	6.88	-	-	1.81	1.81	1.81	1.81	1.81	2.05	-	-	-	-	12.11
20	6.84	-	-	6.83	-	6.87	-	6.88	-	-	1.81	1.81	1.81	1.81	1.81	2.05	-	-	-	-	12.11
21	6.84	-	-	6.83	-	6.87	-	6.87	-	-	1.81	1.81	1.81	1.81	1.81	2.05	-	-	-	-	12.11
22	6.84	-	-	6.85	-	6.87	-	6.88	-	-	1.81	1.81	1.81	1.81	1.81	2.05	-	-	-	-	12.11
23	6.84	-	-	6.85	-	6.87	-	6.87	-	-	1.81	1.81	1.81	1.81	1.81	2.05	-	-	-	-	12.11
24	6.84	-	-	6.86	-	6.87	-	6.89	-	-	1.81	1.81	1.81	1.81	1.81	2.05	-	-	-	-	12.11
25	6.84	-	-	6.86	-	6.86	-	6.89	-	-	1.81	1.81	1.81	1.81	1.81	2.05	-	-	-	-	12.11
26	6.84	-	-	6.86	-	6.86	-	6.88	-	-	1.81	1.81	1.81	1.81	1.81	2.05	-	-	-	-	12.11
27	6.84	-	-	6.86	-	6.86	-	6.88	-	-	1.81	1.81	1.81	1.81	1.81	2.05	-	-	-	-	12.11
28	6.84	-	-	6.85	-	6.87	-	6.88	-	-	1.81	1.81	1.81	1.81	1.81	2.05	-	-	-	-	12.11
29	6.84	-	-	6.85	-	6.87	-	6.88	-	-	1.81	1.81	1.81	1.81	1.81	2.05	-	-	-	-	12.11
30	6.84	-	-	6.85	-	6.86	-	6.88	-	-	1.81	1.81	1.81	1.81	1.81	2.04	-	-	-	-	12.11
31	6.84	-	-	6.86	-	6.87	-	6.88	-	-	1.81	1.81	1.81	1.81	1.81	2.04	-	-	-	-	12.11
32	6.83	-	-	6.85	-	6.87	-	6.88	-	-	1.81	1.81	1.81	1.81	1.81	2.04	-	-	-	-	12.11
33	6.83	-	-	6.86	-	6.87	-	6.89	-	-	1.81	1.81	1.81	1.81	1.80	2.04	-	-	-	-	12.11
34	6.84	-	-	6.86	-	6.88	-	6.88	-	-	1.81	1.81	1.81	1.81	1.81	2.04	-	-	-	-	12.11
35	6.84	-	-	6.86	-	6.87	-	6.88	-	-	1.82	1.81	1.81	1.81	1.81	2.04	-	-	-	-	12.11
36	6.82	-	-	6.86	-	6.86	-	6.88	-	-	1.81	1.81	1.81	1.81	1.81	2.04	-	-	-	-	12.11
37	6.83	-	-	6.86	-	6.87	-	6.88	-	-	1.82	1.81	1.81	1.81	1.81	2.04	-	-	-	-	12.11
38	6.83	-	-	6.87	-	6.87	-	6.88	-	-	1.81	1.81	1.81	1.81	1.81	2.04	-	-	-	-	12.11
39	6.83	-	-	6.87	-	6.87	-	6.88	-	-	1.81	1.81	1.81	1.81	1.81	2.03	-	-	-	-	12.11
40	6.83	-	-	6.86	-	6.87	-	6.88	-	-	1.81	1.82	1.81	1.81	1.81	2.03	-	-	-	-	12.12
41	6.83	-	-	6.86	-	6.87	-	6.87	-	-	1.81	1.82	1.81	1.81	1.81	2.03	-	-	-	-	12.12
42	6.82	-	-	6.87	-	6.87	-	6.87	-	-	1.82	1.82	1.81	1.81	1.80	2.03	-	-	-	-	12.12
43	6.83	-	-	6.87	-	6.88	-	6.87	-	-	1.81	1.82	1.81	1.81	1.81	2.02	-	-	-	-	12.12

44	6.83	-	-	6.88	-	6.88	-	6.86	-	-	1.81	1.82	1.81	1.81	1.81	2.02	-	-	-	-	12.12
45	6.83	-	-	6.88	-	6.87	-	6.85	-	-	1.82	1.82	1.81	1.81	1.80	2.02	-	-	-	-	12.13
46	6.82	-	-	6.88	-	6.87	-	6.86	-	-	1.81	1.82	1.81	1.81	1.80	2.02	-	-	-	-	12.13
47	6.82	-	-	6.87	-	6.88	-	6.86	-	-	1.82	1.82	1.81	1.81	1.80	2.01	-	-	-	-	12.13
48	6.82	-	-	6.88	-	6.88	-	6.87	-	-	1.82	1.82	1.81	1.81	1.80	2.01	-	-	-	-	12.13
49	6.82	-	-	6.88	-	6.89	-	6.87	-	-	1.82	1.82	1.81	1.81	1.80	2.01	-	-	-	-	12.13
50	6.81	-	-	6.88	-	6.88	-	6.87	-	-	1.82	1.82	1.81	1.81	1.81	2.00	-	-	-	-	12.13
51	6.82	-	-	6.88	-	6.88	-	6.87	-	-	1.82	1.83	1.81	1.81	1.81	2.00	-	-	-	-	12.14
52	6.81	-	-	6.88	-	6.88	-	6.87	-	-	1.82	1.83	1.81	1.81	1.81	1.99	-	-	-	-	12.14
53	6.81	-	-	6.88	-	6.89	-	6.88	-	-	1.82	1.83	1.81	1.81	1.81	1.99	-	-	-	-	12.14
54	6.81	-	-	6.89	-	6.88	-	6.88	-	-	1.82	1.83	1.81	1.81	1.80	1.98	-	-	-	-	12.15
55	6.82	-	-	6.89	-	6.89	-	6.88	-	-	1.83	1.83	1.81	1.81	1.80	1.98	-	-	-	-	12.15
56	3.60	3.21	-	6.88	-	3.03	3.86	6.87	-	-	1.83	1.83	1.81	1.81	1.80	1.97	-	-	-	-	12.15
57	3.55	3.27	-	2.92	3.96	3.19	3.70	6.87	-	-	1.83	1.83	1.81	1.81	1.80	1.96	-	-	-	-	12.16
58	3.52	3.30	-	3.09	3.80	3.30	3.60	6.87	-	-	1.83	1.83	1.81	1.81	1.80	1.95	-	-	-	-	12.16
59	3.48	3.33	-	3.21	3.68	3.36	3.53	6.86	-	-	1.83	1.84	1.81	1.81	1.80	1.94	-	-	-	-	12.17
60	3.48	3.34	-	3.29	3.59	3.36	3.52	6.86	-	-	1.83	1.83	1.81	1.81	1.80	1.94	-	-	-	-	12.17
61	3.45	3.36	-	3.37	3.51	3.46	3.43	6.85	-	-	1.83	1.84	1.81	1.81	1.80	1.93	-	-	-	-	12.18
62	3.46	3.37	-	3.41	3.47	3.50	3.40	6.86	-	-	1.84	1.84	1.81	1.81	1.80	1.92	-	-	-	-	12.18
63	3.45	3.38	-	3.45	3.43	3.52	3.38	6.85	-	-	1.84	1.84	1.81	1.81	1.80	1.91	-	-	-	-	12.18
64	3.45	3.38	-	3.49	3.39	3.55	3.35	6.86	-	-	1.84	1.84	1.81	1.81	1.80	1.90	-	-	-	-	12.19
65	3.45	3.38	-	3.52	3.36	3.57	3.33	6.86	-	-	1.84	1.84	1.81	1.81	1.80	1.89	-	-	-	-	12.19
66	3.44	3.39	-	3.56	3.33	3.59	3.31	6.86	-	-	1.84	1.85	1.81	1.81	1.80	1.88	-	-	-	-	12.20
67	3.44	3.39	-	3.58	3.31	3.60	3.30	6.86	-	-	1.85	1.85	1.81	1.81	1.80	1.87	-	-	-	-	12.21
68	3.44	3.40	-	3.61	3.28	3.63	3.27	6.86	-	-	1.85	1.85	1.81	1.81	1.80	1.85	-	-	-	-	12.22
69	3.43	3.40	-	3.64	3.26	3.66	3.25	6.86	-	-	1.85	1.86	1.81	1.81	1.80	1.83	-	-	-	-	12.23
70	3.43	3.40	-	3.67	3.23	3.69	3.22	6.87	-	-	1.86	1.86	1.81	1.81	1.80	1.81	-	-	-	-	12.24
71	3.43	3.40	-	3.70	3.20	3.72	3.18	6.85	-	-	1.87	1.87	1.82	1.81	1.80	1.79	-	-	-	-	12.25
72	3.43	3.40	-	3.74	3.17	3.76	3.15	6.85	-	-	1.87	1.87	1.81	1.81	1.80	1.76	-	-	-	-	12.27
73	3.43	3.41	-	3.79	3.12	3.79	3.13	6.83	-	-	1.89	1.88	1.81	1.81	1.79	1.72	-	-	-	-	12.29
74	3.43	3.41	-	3.84	3.08	3.83	3.09	6.84	-	-	1.90	1.90	1.81	1.81	1.79	1.67	-	-	-	-	12.31
75	3.33	3.28	0.22	3.07	3.85	3.86	3.06	6.85	-	-	1.91	1.91	1.81	1.81	1.79	1.60	-	-	-	-	12.33
76	3.24	3.19	0.39	3.06	3.86	3.86	3.06	6.85	-	-	1.93	1.93	1.82	1.81	1.79	1.53	-	-	-	-	12.37
77	3.16	3.09	0.53	3.07	3.87	3.86	3.07	6.86	-	-	1.94	1.95	1.81	1.81	1.79	1.45	-	-	-	-	12.43
78	2.99	3.05	0.62	3.85	3.09	3.85	3.09	0.98	5.89	-	1.96	1.96	1.81	1.81	1.79	-	-	-	0.76	0.72	12.46

79	2.94	3.06	-	3.83	3.11	3.83	3.10	1.09	5.78	-	1.98	1.98	1.81	1.81	1.79	-	1.45	-	0.60	-	12.49
80	2.88	3.05	-	3.83	3.12	3.81	3.12	1.14	5.74	-	2.00	2.00	1.81	1.81	1.78	-	1.51	-	0.54	-	12.52
81	2.84	3.03	-	3.82	3.13	3.80	3.14	1.15	5.75	-	2.01	2.02	1.81	1.81	1.78	-	1.58	-	0.47	-	12.54
82	2.84	3.01	-	3.81	3.14	3.80	3.14	1.08	5.81	-	2.02	2.02	1.81	1.81	1.78	-	1.60	-	0.43	-	12.55
83	2.83	2.99	-	3.80	3.15	3.79	3.15	1.03	5.87	-	2.03	2.03	1.81	1.81	1.78	-	1.63	-	0.39	-	12.57
84	2.83	2.98	-	3.79	3.17	3.77	3.16	0.96	5.92	-	2.04	2.04	1.81	1.81	1.78	-	1.64	-	0.36	-	12.58
85	2.82	2.96	-	3.77	3.17	3.78	3.16	5.98	0.90	-	2.04	2.04	1.81	1.81	1.78	-	1.66	-	0.33	-	12.59
86	2.84	2.94	-	3.77	3.17	3.77	3.16	6.05	0.82	-	2.05	2.05	1.81	1.80	1.78	-	1.67	-	0.31	-	12.60
87	2.81	2.94	-	3.77	3.18	3.76	3.17	6.12	0.74	-	2.05	2.05	1.81	1.81	1.78	-	1.70	-	0.28	-	12.61
88	2.81	2.93	-	3.77	3.18	3.76	3.17	6.18	0.69	-	2.06	2.05	1.81	1.80	1.78	-	1.70	-	0.26	-	12.62
89	2.80	2.93	-	3.77	3.17	3.76	3.17	6.87	-	-	2.06	2.06	1.81	1.81	1.79	-	1.71	-	0.25	-	12.63
90	2.80	2.92	-	3.77	3.17	3.76	3.18	6.87	-	-	2.06	2.06	1.81	1.81	1.78	-	1.72	-	0.25	-	12.64
91	2.80	2.91	-	3.77	3.18	3.76	3.18	6.87	-	-	2.06	2.06	1.80	1.81	1.79	-	1.73	-	0.24	-	12.64
92	2.79	2.91	-	3.77	3.18	3.75	3.20	6.87	-	-	2.06	2.06	1.80	1.81	1.79	-	1.74	-	0.23	-	12.65
93	2.79	2.89	-	3.76	3.19	3.74	3.20	6.86	-	-	2.06	2.06	1.80	1.81	1.79	-	1.76	-	0.23	-	12.65
94	2.80	2.89	-	3.75	3.19	3.74	3.20	6.86	-	-	2.06	2.06	1.80	1.81	1.79	-	1.76	-	0.23	-	12.66
95	2.79	2.89	-	3.75	3.19	3.74	3.21	6.86	-	-	2.07	2.06	1.80	1.81	1.79	-	1.77	-	0.23	-	12.66
96	2.78	2.89	-	3.75	3.19	3.74	3.21	6.86	-	-	2.07	2.06	1.80	1.81	1.79	-	1.77	-	0.23	-	12.67
97	2.79	2.88	-	3.75	3.19	3.75	3.20	6.86	-	-	2.07	2.06	1.80	1.81	1.79	-	1.77	-	0.23	-	12.67
98	2.78	2.87	-	3.75	3.19	3.74	3.20	6.85	-	-	2.07	2.06	1.80	1.81	1.79	-	1.79	-	0.23	-	12.68
99	2.79	2.86	-	3.74	3.19	3.75	3.20	6.86	-	-	2.07	2.06	1.81	1.81	1.79	-	1.80	-	0.23	-	12.68
100	2.79	2.86	-	3.74	3.18	3.75	3.20	3.49	3.35	-	2.07	2.06	1.80	1.81	1.79	-	1.80	-	0.23	-	12.68
101	2.79	2.86	-	3.74	3.18	3.75	3.19	3.33	3.53	-	2.06	2.06	1.80	1.81	1.79	-	1.81	-	0.23	-	12.69
102	2.87	2.77	-	3.75	3.18	3.19	3.75	3.47	3.39	-	2.06	2.06	1.80	1.81	1.79	-	1.81	-	0.23	-	12.70
103	2.85	2.77	-	3.75	3.17	3.19	3.76	2.35	3.41	1.09	2.06	2.06	1.80	1.81	1.79	-	1.82	-	0.23	-	12.70
104	2.84	2.78	-	3.76	3.18	3.19	3.75	3.38	3.47	-	2.06	2.05	1.80	1.81	1.79	-	1.83	-	0.23	-	12.71
105	2.84	2.77	-	3.76	3.18	3.19	3.76	3.23	3.48	-	2.06	2.05	1.80	1.81	1.79	-	1.83	-	0.23	-	12.71
106	2.83	2.77	-	3.76	3.17	3.18	3.76	3.36	3.49	-	2.06	2.05	1.80	1.81	1.80	-	1.84	-	0.24	-	12.72
107	2.82	2.78	-	3.76	3.17	3.18	3.76	3.36	3.48	-	2.05	2.05	1.80	1.81	1.80	-	1.85	-	0.24	-	12.72
108	2.81	2.77	-	3.78	3.16	3.17	3.78	3.34	3.49	-	2.05	2.04	1.80	1.81	1.80	-	1.85	-	0.24	-	12.72
109	2.81	2.79	-	3.78	3.16	3.16	3.79	3.33	3.51	-	2.05	2.04	1.80	1.81	1.80	-	1.85	-	0.25	-	12.73
110	2.78	2.79	-	3.80	3.16	3.15	3.80	3.32	3.52	-	2.05	2.04	1.80	1.81	1.80	-	1.86	-	0.25	-	12.73
111	2.77	2.79	-	3.79	3.16	3.16	3.79	3.32	3.52	-	2.05	2.03	1.80	1.81	1.80	-	1.87	-	0.26	-	12.74
112	2.75	2.80	-	3.79	3.15	3.15	3.80	3.30	3.53	-	2.04	2.03	1.80	1.81	1.80	-	1.87	-	0.26	-	12.74
113	2.76	2.80	-	3.80	3.14	3.14	3.81	3.29	3.54	-	2.04	2.03	1.80	1.81	1.80	-	1.87	-	0.27	-	12.75

114	2.78	2.77	-	3.81	3.13	3.12	3.82	3.31	3.51	-	2.04	2.02	1.80	1.81	1.80	-	1.87	-	0.27	-	12.75
115	5.55	-	-	3.13	3.82	3.12	3.83	3.28	3.53	-	2.04	2.02	1.80	1.81	1.80	-	1.88	-	0.27	-	12.76
116	5.55	-	-	3.12	3.82	3.12	3.82	3.23	3.33	0.27	2.03	2.02	1.80	1.81	1.80	-	1.89	-	0.27	-	12.76
117	5.54	-	-	3.11	3.83	3.12	3.82	3.22	3.34	0.27	2.03	2.02	1.80	1.81	1.80	-	1.89	-	0.28	-	12.76
118	5.53	-	-	3.10	3.84	3.10	3.84	3.20	3.33	0.29	2.03	2.01	1.80	1.81	1.80	-	1.90	-	0.28	-	12.77
119	5.53	-	-	3.10	3.83	3.09	3.85	3.18	3.33	0.30	2.02	2.01	1.80	1.81	1.80	-	1.89	-	0.29	-	12.77
120	5.53	-	-	3.09	3.84	3.08	3.86	3.18	3.35	0.33	2.03	2.01	1.80	1.81	1.80	-	1.89	-	0.25	-	12.78
121	5.53	-	-	3.08	3.85	3.05	3.88	3.15	3.34	0.34	2.01	2.00	1.80	1.81	1.80	-	1.90	-	0.30	-	12.78
122	5.52	-	-	3.07	3.86	3.06	3.88	3.13	3.34	0.36	2.01	2.00	1.80	1.81	1.80	-	1.90	-	0.30	-	12.79
123	5.52	-	-	3.07	3.86	3.06	3.87	3.10	3.34	0.38	2.01	1.99	1.80	1.81	1.80	-	1.91	-	0.31	-	12.79
124	5.52	-	-	3.06	3.87	6.93	-	3.04	3.36	0.40	2.00	1.99	1.80	1.80	1.80	-	1.91	-	0.31	-	12.80
125	5.53	-	-	6.93	-	6.92	-	2.98	3.38	0.43	2.00	1.98	1.80	1.80	1.80	-	1.91	-	0.32	-	12.80
126	5.51	-	-	6.93	-	6.92	-	3.01	3.34	0.45	1.99	1.98	1.80	1.81	1.80	-	1.91	-	0.32	-	12.81
127	5.51	-	-	6.92	-	6.91	-	6.43	-	0.53	1.99	1.98	1.80	1.80	1.81	-	1.91	-	0.17	-	12.81
128	5.52	-	-	6.92	-	6.91	-	6.34	-	-	1.98	1.97	1.80	1.81	1.81	-	1.91	0.82	-	-	12.81
129	5.51	-	-	6.93	-	6.91	-	6.32	-	-	1.98	1.97	1.80	1.81	1.80	-	1.91	0.84	-	-	12.82
130	5.51	-	-	6.93	-	6.92	-	6.27	-	-	1.97	1.96	1.80	1.81	1.80	-	1.92	0.87	-	-	12.82
131	5.51	-	-	6.93	-	6.92	-	1.97	-	-	1.97	1.96	1.80	1.81	1.80	-	1.92	0.90	-	-	12.82
132	5.51	-	-	6.93	-	6.92	-	6.21	-	-	1.97	1.95	1.80	1.81	1.80	-	1.92	0.92	-	-	12.82
133	5.50	-	-	6.93	-	6.94	-	6.19	-	-	1.96	1.95	1.80	1.81	1.80	-	1.92	0.95	-	-	12.83
134	5.50	-	-	6.94	-	6.93	-	6.18	-	-	1.96	1.95	1.80	1.80	1.81	-	1.92	0.97	-	-	12.83
135	5.49	-	-	6.93	-	6.92	-	6.19	-	-	1.96	1.94	1.80	1.80	1.81	-	1.92	0.99	-	-	12.83
136	5.50	-	-	6.93	-	6.93	-	6.16	-	-	1.95	1.94	1.80	1.80	1.81	-	1.92	1.02	-	-	12.83
137	5.49	-	-	6.93	-	6.92	-	6.15	-	-	1.94	1.93	1.80	1.80	1.81	-	1.92	1.05	-	-	12.83
138	5.50	-	-	6.93	-	6.93	-	6.11	-	-	1.94	1.93	1.80	1.80	1.81	-	1.92	1.07	-	-	12.84
139	5.50	-	-	6.93	-	6.94	-	6.09	-	-	1.94	1.92	1.81	1.80	1.81	-	1.92	1.10	-	-	12.84
140	5.49	-	-	6.93	-	6.94	-	6.07	-	-	1.93	1.92	1.81	1.80	1.81	-	1.92	1.13	-	-	12.84
141	5.50	-	-	6.92	-	6.93	-	6.06	-	-	1.93	1.91	1.81	1.80	1.81	-	1.93	1.15	-	-	12.84
142	5.50	-	-	6.92	-	6.93	-	6.04	-	-	1.92	1.91	1.81	1.81	1.81	-	1.92	1.18	-	-	12.84
143	5.50	-	-	6.93	-	6.92	-	6.01	-	-	1.92	1.90	1.81	1.81	1.81	-	1.93	1.21	-	-	12.84
144	5.50	-	-	6.93	-	6.92	-	6.00	-	-	1.91	1.90	1.81	1.81	1.81	-	1.92	1.23	-	-	12.84
145	5.50	-	-	6.93	-	6.92	-	5.98	-	-	1.91	1.90	1.81	1.80	1.81	-	1.92	1.26	-	-	12.84
146	5.51	-	-	6.93	-	6.92	-	5.96	-	-	1.91	1.89	1.81	1.80	1.81	-	1.92	1.28	-	-	12.84
147	5.51	-	-	6.92	-	6.92	-	5.95	-	-	1.90	1.89	1.81	1.80	1.81	-	1.92	1.30	-	-	12.84
148	5.51	-	-	6.92	-	6.93	-	5.94	-	-	1.90	1.89	1.81	1.80	1.81	-	1.92	1.32	-	-	12.84

149	5.50	-	-	6.91	-	6.92	-	5.93	-	-	1.90	1.88	1.81	1.80	1.81	-	1.92	1.33	-	-	12.84
150	5.51	-	-	6.92	-	6.92	-	5.91	-	-	1.90	1.88	1.81	1.80	1.81	-	1.90	1.35	-	-	12.84
151	5.51	-	-	6.92	-	6.93	-	5.90	-	-	1.90	1.88	1.81	1.81	1.81	-	1.90	1.36	-	-	12.84
152	5.51	-	-	6.92	-	6.93	-	5.90	-	-	1.90	1.88	1.81	1.80	1.81	-	1.91	1.37	-	-	12.84
153	5.51	-	-	6.92	-	6.92	-	5.89	-	-	1.90	1.88	1.81	1.80	1.81	-	1.91	1.38	-	-	12.83
154	5.52	-	-	6.92	-	6.92	-	5.89	-	-	1.90	1.87	1.81	1.80	1.81	-	1.90	1.38	-	-	12.83
155	5.51	-	-	6.92	-	6.92	-	5.89	-	-	1.90	1.88	1.81	1.80	1.81	-	1.90	1.39	-	-	12.83
156	5.52	-	-	6.92	-	6.93	-	5.88	-	-	1.90	1.88	1.82	1.80	1.81	-	1.89	1.39	-	-	12.83
157	5.53	-	-	6.91	-	6.92	-	5.88	-	-	1.89	1.88	1.81	1.80	1.81	-	1.90	1.40	-	-	12.83
158	5.54	-	-	3.82	3.10	6.91	-	5.88	-	-	1.89	1.88	1.82	1.80	1.81	-	1.89	1.40	-	-	12.83
159	5.54	-	-	3.83	3.09	3.13	3.79	5.87	-	-	1.89	1.88	1.81	1.80	1.81	-	1.89	1.40	-	-	12.82
160	5.55	-	-	3.78	3.14	3.14	3.79	5.86	-	-	1.89	1.88	1.81	1.80	1.81	-	1.88	1.40	-	-	12.82
161	5.55	-	-	3.77	3.16	3.15	3.77	5.85	-	-	1.89	1.88	1.81	1.80	1.81	-	1.88	1.41	-	-	12.82
162	5.56	-	-	3.73	3.19	3.18	3.75	5.85	-	-	1.89	1.88	1.81	1.80	1.81	-	1.88	1.41	-	-	12.82
163	5.56	-	-	3.71	3.21	3.21	3.71	5.84	-	-	1.89	1.88	1.81	1.81	1.81	-	1.87	1.41	-	-	12.82
164	5.57	-	-	3.68	3.24	3.25	3.68	5.84	-	-	1.89	1.88	1.81	1.80	1.81	-	1.88	1.41	-	-	12.82
165	5.57	-	-	3.65	3.26	3.29	3.63	5.84	-	-	1.89	1.88	1.81	1.80	1.81	-	1.87	1.41	-	-	12.81
166	5.55	-	-	3.63	3.29	3.31	3.61	5.85	-	-	1.89	1.88	1.81	1.80	1.81	-	1.87	1.42	-	-	12.81
167	5.57	-	-	3.59	3.33	3.34	3.58	5.84	-	-	1.89	1.88	1.81	1.80	1.81	-	1.87	1.41	-	-	12.81
168	5.57	-	-	3.55	3.38	3.36	3.56	5.83	-	-	1.89	1.88	1.81	1.80	1.81	-	1.87	1.42	-	-	12.81
169	5.57	-	-	3.53	3.40	3.40	3.53	5.83	-	-	1.89	1.88	1.81	1.80	1.81	-	1.87	1.42	-	-	12.81
170	5.57	-	-	3.51	3.42	3.43	3.50	5.84	-	-	1.89	1.88	1.81	1.80	1.81	-	1.87	1.41	-	-	12.81
171	5.56	-	-	3.49	3.44	3.47	3.47	5.83	-	-	1.89	1.88	1.81	1.80	1.81	-	1.87	1.41	-	-	12.81
172	5.57	-	-	3.47	3.47	3.49	3.43	5.83	-	-	1.89	1.88	1.81	1.80	1.81	-	1.87	1.42	-	-	12.81
173	5.58	-	-	3.44	3.50	3.50	3.42	5.83	-	-	1.90	1.88	1.81	1.80	1.81	-	1.86	1.41	-	-	12.80
174	5.58	-	-	3.42	3.51	3.52	3.40	5.83	-	-	1.90	1.88	1.81	1.80	1.81	-	1.86	1.42	-	-	12.80
175	5.59	-	-	3.39	3.54	3.53	3.38	5.82	-	-	1.90	1.88	1.81	1.80	1.81	-	1.86	1.42	-	-	12.80
176	5.58	-	-	3.38	3.56	3.57	3.36	5.82	-	-	1.90	1.88	1.81	1.80	1.81	-	1.86	1.42	-	-	12.80
177	5.58	-	-	3.35	3.59	3.58	3.35	5.81	-	-	1.89	1.87	1.81	1.81	1.81	-	1.86	1.42	-	-	12.80
178	5.59	-	-	3.33	3.60	3.62	3.31	5.81	-	-	1.90	1.88	1.81	1.81	1.81	-	1.86	1.43	-	-	12.80
179	5.58	-	-	3.31	3.63	3.63	3.31	5.81	-	-	1.90	1.87	1.81	1.80	1.81	-	1.85	1.43	-	-	12.80
180	5.59	-	-	3.30	3.62	3.64	3.30	5.82	-	-	1.89	1.87	1.81	1.80	1.81	-	1.85	1.43	-	-	12.80
181	5.59	-	-	3.28	3.62	3.66	3.28	5.82	-	-	1.89	1.87	1.81	1.80	1.81	-	1.86	1.43	-	-	12.80
182	5.59	-	-	3.28	3.63	3.65	3.28	5.82	-	-	1.89	1.87	1.82	1.80	1.81	-	1.86	1.43	-	-	12.81
183	5.58	-	-	3.28	3.65	3.65	3.28	5.81	-	-	1.90	1.87	1.81	1.80	1.81	-	1.85	1.43	-	-	12.81

184	5.59	-	-	3.28	3.66	3.66	3.27	5.83	-	-	1.90	1.87	1.81	1.80	1.81	-	1.85	1.43	-	-	12.81
185	5.58	-	-	3.30	3.62	3.66	3.26	5.83	-	-	1.90	1.88	1.81	1.80	1.81	-	1.85	1.43	-	-	12.80
186	5.59	-	-	3.29	3.62	3.64	3.28	5.83	-	-	1.90	1.88	1.81	1.80	1.81	-	1.85	1.43	-	-	12.81
187	5.58	-	-	3.28	3.62	3.64	3.29	5.83	-	-	1.89	1.88	1.81	1.80	1.81	-	1.85	1.43	-	-	12.80
188	5.58	-	-	3.29	3.63	3.65	3.28	5.82	-	-	1.89	1.88	1.82	1.80	1.81	-	1.85	1.43	-	-	12.81
189	5.57	-	-	3.29	3.64	3.27	3.67	5.82	-	-	1.89	1.88	1.81	1.81	1.81	-	1.86	1.43	-	-	12.81
190	5.58	-	-	3.29	3.65	3.27	3.67	5.82	-	-	1.90	1.88	1.81	1.81	1.81	-	1.85	1.43	-	-	12.81
191	5.59	-	-	3.28	3.65	3.66	3.28	5.81	-	-	1.89	1.88	1.81	1.81	1.81	-	1.85	1.43	-	-	12.81
192	5.58	-	-	3.28	3.64	3.66	3.27	5.82	-	-	1.89	1.88	1.81	1.80	1.81	-	1.86	1.43	-	-	12.81
193	5.58	-	-	3.28	3.64	3.66	3.28	5.82	-	-	1.89	1.88	1.81	1.80	1.81	-	1.86	1.42	-	-	12.81
194	5.58	-	-	3.28	3.65	3.67	3.26	5.81	-	-	1.89	1.88	1.81	1.80	1.81	-	1.86	1.42	-	-	12.81
195	5.59	-	-	3.28	3.65	3.66	3.26	5.82	-	-	1.89	1.88	1.81	1.80	1.81	-	1.86	1.43	-	-	12.81
196	5.59	-	-	3.29	3.64	3.65	3.28	5.82	-	-	1.90	1.88	1.82	1.80	1.81	-	1.85	1.43	-	-	12.81
197	5.58	-	-	3.28	3.65	3.66	3.27	5.82	-	-	1.90	1.88	1.82	1.81	1.81	-	1.86	1.43	-	-	12.81
198	5.58	-	-	3.29	3.64	3.65	3.28	5.82	-	-	1.90	1.88	1.81	1.81	1.81	-	1.86	1.43	-	-	12.81
199	5.58	-	-	3.29	3.65	3.65	3.28	5.82	-	-	1.90	1.87	1.82	1.81	1.81	-	1.86	1.43	-	-	12.81
200	5.58	-	-	3.28	3.66	3.66	3.26	5.82	-	-	1.90	1.88	1.82	1.81	1.81	-	1.86	1.43	-	-	12.81
201	5.58	-	-	3.28	3.64	3.26	3.67	5.80	-	-	1.90	1.87	1.82	1.81	1.81	-	1.86	1.43	-	-	12.82
202	5.58	-	-	3.28	3.65	3.65	3.28	5.82	-	-	1.90	1.87	1.82	1.81	1.81	-	1.86	1.43	-	-	12.82
203	5.59	-	-	3.28	3.65	3.66	3.27	5.81	-	-	1.90	1.87	1.82	1.81	1.81	-	1.86	1.43	-	-	12.82
204	5.59	-	-	3.29	3.64	3.66	3.28	5.81	-	-	1.90	1.87	1.82	1.80	1.81	-	1.86	1.43	-	-	12.82
205	5.58	-	-	3.29	3.63	3.65	3.27	5.81	-	-	1.90	1.87	1.82	1.80	1.81	-	1.86	1.43	-	-	12.82
206	5.58	-	-	3.29	3.63	3.64	3.30	5.81	-	-	1.90	1.88	1.82	1.80	1.81	-	1.85	1.42	-	-	12.82
207	5.58	-	-	3.28	3.63	3.64	3.30	5.81	-	-	1.90	1.88	1.81	1.80	1.80	-	1.85	1.42	-	-	12.82
208	5.58	-	-	3.28	3.64	3.65	3.29	5.81	-	-	1.90	1.87	1.81	1.80	1.81	-	1.86	1.42	-	-	12.82
209	5.58	-	-	3.29	3.64	3.65	3.29	5.82	-	-	1.90	1.87	1.81	1.81	1.81	-	1.86	1.42	-	-	12.82
210	5.58	-	-	3.29	3.64	3.65	3.28	5.82	-	-	1.90	1.87	1.81	1.81	1.81	-	1.85	1.43	-	-	12.82
211	5.59	-	-	3.29	3.65	3.65	3.27	5.82	-	-	1.90	1.87	1.81	1.81	1.81	-	1.85	1.43	-	-	12.82
212	5.58	-	-	3.29	3.65	3.65	3.27	5.81	-	-	1.90	1.87	1.82	1.81	1.81	-	1.86	1.43	-	-	12.82
213	5.58	-	-	3.28	3.65	3.64	3.28	5.81	-	-	1.90	1.87	1.82	1.81	1.81	-	1.86	1.43	-	-	12.82
214	5.58	-	-	3.28	3.65	3.63	3.28	5.81	-	-	1.90	1.87	1.82	1.80	1.81	-	1.86	1.43	-	-	12.82
215	5.58	-	-	3.28	3.65	3.65	3.27	5.81	-	-	1.90	1.87	1.82	1.80	1.81	-	1.86	1.43	-	-	12.82
216	5.59	-	-	3.29	3.64	3.65	3.27	5.80	-	-	1.90	1.88	1.82	1.80	1.81	-	1.85	1.43	-	-	12.82
217	5.58	-	-	3.29	3.65	3.65	3.28	5.80	-	-	1.90	1.88	1.81	1.80	1.81	-	1.85	1.43	-	-	12.82
218	5.59	-	-	3.28	3.65	3.65	3.28	5.80	-	-	1.91	1.88	1.81	1.81	1.80	-	1.85	1.43	-	-	12.82

219	5.58	-	-	3.26	3.66	3.65	3.28	5.81	-	-	1.91	1.88	1.81	1.81	1.80	-	1.85	1.43	-	-	12.82
220	5.59	-	-	3.27	3.66	3.66	3.27	5.82	-	-	1.90	1.88	1.81	1.81	1.81	-	1.85	1.43	-	-	12.82
221	5.59	-	-	3.28	3.66	3.66	3.27	5.82	-	-	1.90	1.87	1.81	1.81	1.81	-	1.85	1.43	-	-	12.82
222	5.60	-	-	3.28	3.65	3.65	3.28	5.82	-	-	1.91	1.87	1.82	1.81	1.81	-	1.85	1.43	-	-	12.82
223	5.59	-	-	3.27	3.65	3.65	3.28	5.82	-	-	1.90	1.87	1.82	1.81	1.81	-	1.85	1.43	-	-	12.82
224	5.59	-	-	3.28	3.65	3.66	3.28	5.82	-	-	1.90	1.87	1.81	1.81	1.81	-	1.85	1.43	-	-	12.82
225	5.59	-	-	3.28	3.65	3.66	3.28	5.81	-	-	1.90	1.87	1.82	1.81	1.81	-	1.85	1.43	-	-	12.82
226	5.59	-	-	3.28	3.65	3.66	3.27	5.81	-	-	1.90	1.87	1.82	1.81	1.81	-	1.86	1.43	-	-	12.82
227	5.59	-	-	3.28	3.64	3.66	3.27	5.81	-	-	1.90	1.87	1.82	1.80	1.82	-	1.86	1.43	-	-	12.82
228	5.59	-	-	3.29	3.63	3.66	3.27	5.81	-	-	1.90	1.87	1.82	1.81	1.82	-	1.86	1.43	-	-	12.82
229	5.60	-	-	3.28	3.64	3.66	3.27	5.81	-	-	1.90	1.87	1.82	1.80	1.81	-	1.86	1.43	-	-	12.82
230	5.59	-	-	3.28	3.65	3.67	3.27	5.81	-	-	1.91	1.87	1.81	1.81	1.81	-	1.86	1.43	-	-	12.82
231	5.59	-	-	3.28	3.65	3.67	3.27	5.82	-	-	1.91	1.87	1.82	1.81	1.80	-	1.86	1.43	-	-	12.82
232	5.59	-	-	3.28	3.65	3.67	3.27	5.81	-	-	1.91	1.87	1.82	1.81	1.80	-	1.85	1.43	-	-	12.82
233	5.59	-	-	3.28	3.66	3.25	3.69	5.81	-	-	1.90	1.87	1.82	1.81	1.80	-	1.85	1.43	-	-	12.82
234	5.59	-	-	3.29	3.65	3.66	3.27	5.81	-	-	1.90	1.87	1.82	1.81	1.80	-	1.86	1.43	-	-	12.82
235	5.59	-	-	3.29	3.66	3.66	3.28	5.81	-	-	1.90	1.87	1.82	1.81	1.80	-	1.85	1.43	-	-	12.82
236	5.58	-	-	3.28	3.66	3.26	3.68	5.81	-	-	1.91	1.86	1.82	1.81	1.81	-	1.86	1.43	-	-	12.82
237	5.58	-	-	3.28	3.66	3.65	3.27	5.81	-	-	1.91	1.87	1.81	1.81	1.81	-	1.86	1.43	-	-	12.82
238	5.59	-	-	3.28	3.66	3.24	3.69	5.80	-	-	1.91	1.87	1.81	1.81	1.81	-	1.85	1.43	-	-	12.82
239	5.58	-	-	3.27	3.65	3.66	3.27	5.81	-	-	1.91	1.87	1.82	1.80	1.81	-	1.86	1.43	-	-	12.82
240	5.58	-	-	3.28	3.65	3.66	3.27	5.81	-	-	1.91	1.87	1.81	1.80	1.81	-	1.86	1.43	-	-	12.83
241	5.58	-	-	3.27	3.65	3.66	3.28	5.81	-	-	1.91	1.87	1.82	1.80	1.82	-	1.86	1.43	-	-	12.82
242	5.57	-	-	3.27	3.65	3.66	3.28	5.81	-	-	1.91	1.87	1.82	1.81	1.81	-	1.86	1.43	-	-	12.83
243	5.58	-	-	3.28	3.65	3.65	3.27	5.81	-	-	1.91	1.87	1.81	1.81	1.81	-	1.86	1.43	-	-	12.83
244	5.59	-	-	3.27	3.66	3.65	3.27	5.82	-	-	1.90	1.87	1.81	1.81	1.81	-	1.86	1.43	-	-	12.83
245	5.58	-	-	3.27	3.66	3.66	3.27	5.81	-	-	1.91	1.87	1.82	1.80	1.80	-	1.86	1.43	-	-	12.83
246	5.58	-	-	3.27	3.66	3.66	3.26	5.80	-	-	1.91	1.87	1.82	1.81	1.80	-	1.86	1.43	-	-	12.83
247	5.58	-	-	3.27	3.66	3.67	3.26	5.81	-	-	1.91	1.87	1.82	1.81	1.80	-	1.85	1.44	-	-	12.83
248	5.57	-	-	3.28	3.66	3.67	3.25	5.81	-	-	1.91	1.86	1.82	1.81	1.80	-	1.86	1.44	-	-	12.82
249	5.57	-	-	3.28	3.66	3.67	3.27	5.80	-	-	1.91	1.87	1.82	1.81	1.80	-	1.86	1.43	-	-	12.82
250	5.58	-	-	3.27	3.66	3.66	3.26	5.80	-	-	1.91	1.87	1.82	1.81	1.80	-	1.86	1.43	-	-	12.83
251	5.58	-	-	3.27	3.66	3.66	3.27	5.80	-	-	1.91	1.87	1.82	1.80	1.81	-	1.85	1.43	-	-	12.83
252	5.58	-	-	3.27	3.66	3.67	3.26	5.80	-	-	1.91	1.87	1.82	1.80	1.81	-	1.85	1.43	-	-	12.83
253	5.59	-	-	3.27	3.66	3.68	3.25	5.81	-	-	1.91	1.87	1.82	1.80	1.81	-	1.86	1.43	-	-	12.83

254	5.59	-	-	3.26	3.67	3.68	3.25	5.81	-	-	1.90	1.87	1.82	1.80	1.81	-	1.86	1.43	-	-	12.83
255	5.58	-	-	3.27	3.66	3.68	3.25	5.80	-	-	1.91	1.87	1.82	1.80	1.81	-	1.86	1.43	-	-	12.83
256	5.58	-	-	3.27	3.66	3.67	3.25	5.80	-	-	1.91	1.87	1.82	1.80	1.81	-	1.86	1.44	-	-	12.83
257	5.59	-	-	3.27	3.66	3.68	3.25	5.80	-	-	1.91	1.87	1.82	1.81	1.81	-	1.86	1.44	-	-	12.83
258	5.58	-	-	3.27	3.66	3.67	3.25	5.80	-	-	1.91	1.87	1.82	1.81	1.81	-	1.86	1.44	-	-	12.83
259	5.58	-	-	3.27	3.66	3.68	3.24	5.79	-	-	1.91	1.87	1.82	1.81	1.81	-	1.86	1.43	-	-	12.83
260	5.58	-	-	3.27	3.66	3.68	3.24	5.80	-	-	1.91	1.87	1.82	1.81	1.81	-	1.86	1.43	-	-	12.83
261	5.58	-	-	3.27	3.66	3.67	3.26	5.81	-	-	1.91	1.87	1.82	1.81	1.80	-	1.86	1.42	-	-	12.83
262	5.58	-	-	3.27	3.66	3.67	3.26	5.81	-	-	1.91	1.87	1.82	1.81	1.80	-	1.86	1.43	-	-	12.83
263	5.58	-	-	3.27	3.66	3.68	3.25	5.80	-	-	1.91	1.87	1.82	1.81	1.80	-	1.86	1.43	-	-	12.83
264	5.58	-	-	3.26	3.67	3.68	3.25	5.80	-	-	1.91	1.87	1.82	1.81	1.81	-	1.86	1.43	-	-	12.83
265	5.58	-	-	3.26	3.66	3.68	3.26	5.80	-	-	1.91	1.87	1.82	1.80	1.80	-	1.86	1.43	-	-	12.83
266	5.58	-	-	3.24	3.67	3.68	3.25	5.79	-	-	1.91	1.87	1.82	1.81	1.81	-	1.86	1.43	-	-	12.83
267	5.59	-	-	3.25	3.65	3.68	3.25	5.79	-	-	1.91	1.87	1.82	1.81	1.81	-	1.86	1.43	-	-	12.83

*ELF analysis for oxidation of furan **R2** and tetrehydrothiophene **R3***

Table S12. ELF basin populations for the oxidation of **R2**

	V1(O3)	V2(O3)	V3(O3)	V1(O4)	V2(O4)	V1(O5)	V2(O5)	V1(O6)	V2(O6)	V(C1,O10)	V(C9,O10)	V(C1,C2)	V(C2,C8)	V(C8,C9)	V1(O10)	V2(O10)	V(O3,H)	V(C1,H)	V(C1,O6)	V(C1)	V(H)	Ru
1	6.86	-	-	6.86	-	6.84	-	6.90	-	1.36	1.35	1.86	1.79	1.87	2.48	2.21	-	2.06	-	-	-	
2	6.86	-	-	6.86	-	6.86	-	6.90	-	1.36	1.35	1.86	1.79	1.87	2.48	2.21	-	2.06	-	-	-	12.13
3	6.86	-	-	6.86	-	6.86	-	6.90	-	1.36	1.35	1.87	1.79	1.87	2.21	2.48	-	2.07	-	-	-	12.13
4	6.86	-	-	6.87	-	6.86	-	6.90	-	1.36	1.35	1.86	1.79	1.87	2.47	2.21	-	2.07	-	-	-	12.13
5	6.86	-	-	6.87	-	6.87	-	6.91	-	1.36	1.35	1.87	1.79	1.87	2.21	2.48	-	2.06	-	-	-	12.13
6	6.86	-	-	6.86	-	6.87	-	6.91	-	1.35	1.35	1.87	1.79	1.87	2.22	2.48	-	2.06	-	-	-	12.14
7	6.86	-	-	6.86	-	6.87	-	6.91	-	1.35	1.35	1.87	1.79	1.87	2.49	2.22	-	2.06	-	-	-	12.14
8	6.85	-	-	6.86	-	6.86	-	6.90	-	1.36	1.35	1.87	1.79	1.87	2.48	2.22	-	2.06	-	-	-	12.13
9	6.85	-	-	6.85	-	6.87	-	6.89	-	1.36	1.36	1.87	1.79	1.87	2.21	2.48	-	2.06	-	-	-	12.13
10	6.86	-	-	6.84	-	6.87	-	6.90	-	1.36	1.36	1.87	1.79	1.87	2.22	2.48	-	2.06	-	-	-	12.13
11	6.85	-	-	6.85	-	6.87	-	6.90	-	1.36	1.36	1.87	1.79	1.87	2.22	2.47	-	2.06	-	-	-	12.14
12	6.86	-	-	6.86	-	6.87	-	6.90	-	1.36	1.36	1.86	1.79	1.87	2.22	2.47	-	2.07	-	-	-	12.14
13	6.86	-	-	6.87	-	6.87	-	6.90	-	1.36	1.36	1.87	1.79	1.87	2.21	2.47	-	2.06	-	-	-	12.14
14	6.85	-	-	6.87	-	6.86	-	6.91	-	1.36	1.36	1.87	1.79	1.87	2.21	2.48	-	2.06	-	-	-	12.14
15	6.85	-	-	6.87	-	6.87	-	6.90	-	1.36	1.35	1.87	1.79	1.87	2.21	2.48	-	2.06	-	-	-	12.14
16	6.86	-	-	6.86	-	6.87	-	6.90	-	1.36	1.36	1.87	1.79	1.87	2.21	2.47	-	2.06	-	-	-	12.14
17	6.85	-	-	6.86	-	6.85	-	6.89	-	1.36	1.35	1.87	1.79	1.87	2.22	2.48	-	2.06	-	-	-	12.14
18	6.85	-	-	6.86	-	6.87	-	6.90	-	1.36	1.35	1.87	1.79	1.87	2.21	2.48	-	2.06	-	-	-	12.14
19	6.85	-	-	6.85	-	6.86	-	6.88	-	1.36	1.36	1.87	1.79	1.87	2.48	2.21	-	2.06	-	-	-	12.15
20	6.85	-	-	6.85	-	6.87	-	6.88	-	1.37	1.36	1.87	1.79	1.87	2.47	2.21	-	2.06	-	-	-	12.16
21	6.85	-	-	6.85	-	6.85	-	6.89	-	1.36	1.35	1.87	1.79	1.87	2.48	2.20	-	2.06	-	-	-	12.16
22	6.84	-	-	6.85	-	6.86	-	6.89	-	1.36	1.36	1.87	1.79	1.87	2.21	2.47	-	2.06	-	-	-	12.17
23	6.85	-	-	6.86	-	6.86	-	6.90	-	1.37	1.35	1.87	1.79	1.87	2.21	2.48	-	2.06	-	-	-	12.17
24	6.85	-	-	6.86	-	6.88	-	6.90	-	1.37	1.35	1.87	1.79	1.87	2.21	2.47	-	2.06	-	-	-	12.16
25	6.85	-	-	6.87	-	6.84	-	6.90	-	1.36	1.36	1.87	1.79	1.87	2.21	2.48	-	2.05	-	-	-	12.15
26	6.84	-	-	6.86	-	6.88	-	6.90	-	1.37	1.35	1.87	1.79	1.87	2.48	2.21	-	2.06	-	-	-	12.14
27	6.85	-	-	6.86	-	6.87	-	6.91	-	1.37	1.35	1.87	1.79	1.87	2.47	2.21	-	2.06	-	-	-	12.15

28	6.84	-	-	6.87	-	6.88	-	6.91	-	1.37	1.36	1.87	1.79	1.87	2.48	2.20	-	2.06	-	-	-	12.14
29	6.85	-	-	6.86	-	6.86	-	6.90	-	1.37	1.36	1.87	1.79	1.87	2.47	2.20	-	2.06	-	-	-	12.14
30	6.85	-	-	6.87	-	6.87	-	6.90	-	1.37	1.35	1.87	1.79	1.87	2.47	2.21	-	2.05	-	-	-	12.14
31	6.85	-	-	6.87	-	6.89	-	6.91	-	1.36	1.36	1.87	1.79	1.87	2.48	2.20	-	2.06	-	-	-	12.14
32	6.85	-	-	6.86	-	6.88	-	6.91	-	1.37	1.36	1.87	1.79	1.87	2.47	2.19	-	2.05	-	-	-	12.14
33	6.85	-	-	6.87	-	6.88	-	6.91	-	1.38	1.36	1.87	1.79	1.88	2.47	2.19	-	2.05	-	-	-	12.14
34	6.85	-	-	6.87	-	6.88	-	6.91	-	1.37	1.35	1.87	1.78	1.88	2.48	2.19	-	2.05	-	-	-	12.14
35	6.85	-	-	6.87	-	6.89	-	6.91	-	1.37	1.35	1.87	1.79	1.87	2.48	2.19	-	2.05	-	-	-	12.14
36	6.84	-	-	6.88	-	6.89	-	6.91	-	1.37	1.35	1.87	1.79	1.87	2.48	2.20	-	2.05	-	-	-	12.14
37	6.84	-	-	6.88	-	6.88	-	6.91	-	1.37	1.36	1.87	1.79	1.87	2.48	2.19	-	2.05	-	-	-	12.14
38	6.84	-	-	6.87	-	6.89	-	6.91	-	1.37	1.36	1.87	1.79	1.87	2.47	2.19	-	2.05	-	-	-	12.14
39	6.84	-	-	6.88	-	6.89	-	6.90	-	1.38	1.36	1.87	1.79	1.87	2.47	2.19	-	2.05	-	-	-	12.14
40	6.84	-	-	6.88	-	6.89	-	6.90	-	1.38	1.36	1.88	1.79	1.87	2.47	2.20	-	2.05	-	-	-	12.15
41	6.84	-	-	6.87	-	6.89	-	6.90	-	1.37	1.36	1.88	1.79	1.87	2.47	2.20	-	2.05	-	-	-	12.15
42	6.84	-	-	6.88	-	6.89	-	6.91	-	1.38	1.36	1.88	1.79	1.88	2.47	2.19	-	2.04	-	-	-	12.15
43	6.85	-	-	6.88	-	6.90	-	6.91	-	1.38	1.36	1.87	1.79	1.87	2.47	2.18	-	2.04	-	-	-	12.15
44	6.84	-	-	6.88	-	6.89	-	6.92	-	1.38	1.36	1.88	1.79	1.87	2.48	2.18	-	2.04	-	-	-	12.15
45	6.84	-	-	6.88	-	6.88	-	6.92	-	1.39	1.36	1.88	1.79	1.87	2.48	2.18	-	2.04	-	-	-	12.15
46	6.83	-	-	6.88	-	6.89	-	6.92	-	1.39	1.36	1.88	1.79	1.87	2.48	2.17	-	2.04	-	-	-	12.15
47	6.83	-	-	6.89	-	6.88	-	6.91	-	1.39	1.35	1.88	1.79	1.87	2.47	2.17	-	2.04	-	-	-	12.15
48	6.84	-	-	6.89	-	6.88	-	6.91	-	1.39	1.36	1.88	1.78	1.87	2.48	2.17	-	2.03	-	-	-	12.15
49	6.84	-	-	6.88	-	6.88	-	6.92	-	1.39	1.36	1.88	1.78	1.87	2.48	2.16	-	2.03	-	-	-	12.16
50	6.83	-	-	6.89	-	6.89	-	6.92	-	1.39	1.36	1.88	1.79	1.88	2.49	2.16	-	2.02	-	-	-	12.16
51	6.83	-	-	6.89	-	6.88	-	6.92	-	1.40	1.36	1.88	1.79	1.87	2.49	2.14	-	2.02	-	-	-	12.16
52	6.83	-	-	6.89	-	6.90	-	6.92	-	1.40	1.36	1.88	1.79	1.88	2.49	2.13	-	2.02	-	-	-	12.16
53	6.83	-	-	6.89	-	6.90	-	6.92	-	1.40	1.36	1.88	1.79	1.87	2.49	2.12	-	2.01	-	-	-	12.16
54	6.82	-	-	6.89	-	6.90	-	6.91	-	1.41	1.37	1.88	1.79	1.87	2.48	2.12	-	2.01	-	-	-	12.17
55	6.83	-	-	6.89	-	6.90	-	6.92	-	1.41	1.37	1.88	1.79	1.87	2.49	2.12	-	2.00	-	-	-	12.17
56	6.83	-	-	6.89	-	6.90	-	6.91	-	1.41	1.37	1.88	1.79	1.87	2.50	2.12	-	2.00	-	-	-	12.18
57	6.83	-	-	6.89	-	6.90	-	6.91	-	1.42	1.37	1.89	1.79	1.88	2.51	2.10	-	1.99	-	-	-	12.18
58	3.30	3.53	-	6.89	-	4.19	2.71	6.91	-	1.42	1.37	1.89	1.79	1.88	2.51	2.08	-	1.99	-	-	-	12.19
59	3.23	3.60	-	6.89	-	4.07	2.83	6.91	-	1.42	1.37	1.89	1.79	1.88	2.52	2.07	-	1.98	-	-	-	12.19
60	3.27	3.56	-	4.13	2.75	4.00	2.91	6.91	-	1.42	1.37	1.89	1.79	1.88	2.52	2.04	-	1.97	-	-	-	12.2
61	3.29	3.54	-	4.04	2.85	3.95	2.95	6.91	-	1.43	1.37	1.89	1.79	1.88	2.53	2.02	-	1.97	-	-	-	12.2
62	3.33	3.51	-	3.99	2.90	3.88	3.02	6.90	-	1.44	1.37	1.89	1.79	1.88	2.54	2.01	-	1.96	-	-	-	12.2

63	3.35	3.49	-	3.97	2.91	3.87	3.04	6.91	-	1.45	1.37	1.90	1.79	1.88	2.56	1.98	-	1.95	-	-	-	12.21
64	3.38	3.47	-	3.94	2.93	3.84	3.06	1.87	5.04	1.45	1.37	1.90	1.79	1.88	2.59	1.95	-	1.94	-	-	-	12.21
65	3.41	3.44	-	3.89	2.98	3.82	3.09	2.19	4.73	1.46	1.37	1.90	1.79	1.88	2.62	1.92	-	1.93	-	-	-	12.22
66	3.42	3.43	-	3.89	2.99	3.81	3.10	2.42	4.50	1.47	1.37	1.90	1.78	1.88	2.64	1.89	-	1.91	-	-	-	12.22
67	3.43	3.42	-	3.89	2.99	3.80	3.10	2.58	4.34	1.50	1.39	1.90	1.79	1.88	4.48	-	-	1.90	-	-	-	12.23
68	3.42	3.42	-	3.90	2.99	3.80	3.10	2.72	4.19	1.51	1.39	1.90	1.78	1.88	4.46	-	-	1.88	-	-	-	12.24
69	3.43	3.41	-	3.92	2.97	3.81	3.09	2.84	4.08	1.52	1.39	1.90	1.79	1.88	4.44	-	-	1.86	-	-	-	12.25
70	3.43	3.42	-	3.93	2.97	3.83	3.08	2.91	4.00	1.54	1.39	1.91	1.79	1.88	4.43	-	-	1.84	-	-	-	12.27
71	3.44	3.41	-	3.95	2.96	3.85	3.06	2.98	3.94	1.55	1.39	1.91	1.78	1.88	4.40	-	-	1.80	-	-	-	12.28
72	3.43	3.42	-	3.96	2.96	3.86	3.06	3.03	3.91	1.57	1.39	1.93	1.78	1.88	4.39	-	-	1.75	-	-	-	12.3
73	3.34	3.25	0.24	2.97	3.94	3.86	3.07	3.07	3.88	1.61	1.41	1.94	1.78	1.88	4.34	-	-	1.70	-	-	-	12.33
74	3.26	3.16	0.39	3.00	3.92	3.85	3.08	3.09	3.87	1.63	1.41	1.95	1.78	1.88	4.31	-	-	1.64	-	-	-	12.36
75	3.20	3.06	0.53	3.90	3.03	3.10	3.85	3.87	3.09	1.65	1.41	1.97	1.78	1.88	4.28	-	-	-	-	0.82	0.75	12.42
76	3.17	2.94	0.65	3.87	3.07	3.13	3.82	3.88	3.08	1.68	1.42	1.99	1.78	1.88	4.24	-	-	-	-	0.78	0.74	12.46
77	3.19	2.84	-	3.84	3.10	3.79	3.16	3.07	3.90	1.71	1.42	2.01	1.78	1.88	4.21	-	1.45	-	-	0.72	-	12.49
78	5.95	-	-	3.81	3.13	3.77	3.17	3.07	3.91	1.74	1.42	2.03	1.78	1.89	4.17	-	1.52	-	-	0.66	-	12.52
79	3.14	2.78	-	3.79	3.15	3.75	3.20	3.90	3.08	1.77	1.42	2.05	1.78	1.89	4.14	-	1.55	-	-	0.60	-	12.54
80	3.11	2.78	-	3.78	3.17	3.74	3.21	3.91	3.08	1.80	1.43	2.06	1.77	1.89	4.11	-	1.59	-	-	0.55	-	12.56
81	3.08	2.80	-	3.77	3.18	3.74	3.22	3.92	3.06	1.82	1.43	2.07	1.78	1.89	4.10	-	1.60	-	-	0.51	-	12.57
82	3.04	2.82	-	3.75	3.19	3.73	3.23	3.93	3.05	1.84	1.42	2.09	1.78	1.89	4.08	-	1.62	-	-	0.47	-	12.59
83	3.01	2.83	-	3.21	3.74	3.72	3.25	3.03	3.94	1.86	1.42	2.09	1.78	1.89	4.07	-	1.64	-	-	0.43	-	12.6
84	3.01	2.84	-.	3.22	3.73	3.70	3.26	3.03	3.92	1.87	1.42	2.10	1.78	1.89	4.06	-	1.64	-	-	0.41	-	12.61
85	2.99	2.85	-	3.72	3.23	3.70	3.26	3.02	3.96	1.88	1.42	2.11	1.78	1.89	4.05	-	1.65	-	-	0.38	-	12.62
86	2.96	2.86	-	3.72	3.24	3.70	3.26	3.02	3.98	1.90	1.42	2.12	1.78	1.89	4.04	-	1.67	-	-	0.36	-	12.63
87	2.95	2.86	-	3.71	3.25	3.69	3.27	3.01	3.98	1.91	1.42	2.13	1.78	1.89	4.03	-	1.67	-	-	0.34	-	12.63
88	2.93	2.87	-	3.24	3.70	3.69	3.27	3.99	2.99	1.92	1.43	2.13	1.78	1.89	4.02	-	1.68	-	-	0.32	-	12.64
89	2.92	2.87	-	3.25	3.70	3.68	3.28	4.02	2.97	1.93	1.42	2.14	1.78	1.89	4.01	-	1.70	-	-	0.30	-	12.65
90	2.93	2.86	-	3.70	3.26	3.68	3.29	2.95	4.03	1.94	1.43	2.14	1.78	1.89	4.01	-	1.70	-	-	0.29	-	12.65
91	2.92	2.86	-	3.70	3.26	3.68	3.29	2.93	4.06	1.95	1.43	2.15	1.78	1.89	4.00	-	1.71	-	-	0.27	-	12.66
92	2.90	2.88	-	3.70	3.26	3.68	3.30	2.90	4.09	1.95	1.43	2.15	1.78	1.89	4.00	-	1.71	-	-	0.26	-	12.66
93	2.87	2.90	-	3.69	3.27	3.67	3.30	2.87	4.12	1.96	1.42	2.16	1.78	1.89	4.00	-	1.72	-	-	0.25	-	12.66
94	2.86	2.91	-	3.69	3.27	3.67	3.30	2.85	4.14	1.96	1.42	2.16	1.78	1.89	4.00	-	1.72	-	-	0.24	-	12.67
95	2.86	2.91	-	3.68	3.27	3.67	3.30	2.82	4.17	1.97	1.43	2.16	1.78	1.89	3.99	-	1.72	-	-	0.23	-	12.67
96	2.83	2.92	-	3.68	3.28	3.66	3.31	2.78	4.22	1.97	1.43	2.16	1.78	1.89	3.99	-	1.73	-	-	0.23	-	12.68
97	2.83	2.92	-	3.68	3.27	3.65	3.31	2.72	4.27	1.97	1.43	2.16	1.78	1.89	3.99	-	1.74	-	-	0.23	-	12.68

98	2.82	2.93	-	3.67	3.29	3.65	3.32	2.69	4.29	1.97	1.42	2.16	1.78	1.89	4.00	-	1.74	-	-	-	-	12.69
99	2.81	2.94	-	3.29	3.67	3.65	3.32	4.34	2.65	1.97	1.42	2.16	1.78	1.89	4.00	-	1.75	-	-	-	-	12.69
100	2.79	2.94	-	3.28	3.67	3.65	3.31	4.39	2.60	1.98	1.42	2.16	1.78	1.89	4.00	-	1.75	-	-	-	-	12.69
101	2.79	2.94	-	3.29	3.66	3.65	3.31	4.42	2.56	1.98	1.42	2.16	1.78	1.89	4.00	-	1.76	-	-	-	-	12.69
102	2.78	2.96	-	3.29	3.66	3.65	3.31	4.46	2.51	1.98	1.42	2.16	1.78	1.89	4.01	-	1.76	-	-	-	-	12.7
103	2.76	2.96	-	3.30	3.66	3.65	3.31	4.51	2.47	1.97	1.42	2.16	1.77	1.89	4.01	-	1.76	-	-	-	-	12.7
104	2.76	2.95	-	3.29	3.67	3.65	3.31	4.56	2.42	1.97	1.42	2.16	1.77	1.89	4.01	-	1.77	-	-	-	-	12.7
105	2.74	2.97	-	3.28	3.67	3.65	3.31	4.60	2.36	1.97	1.42	2.16	1.77	1.89	4.02	-	1.77	-	-	-	-	12.7
106	2.73	2.97	-	3.28	3.68	3.65	3.31	4.67	2.30	1.98	1.42	2.16	1.77	1.89	4.01	-	1.78	-	-	-	-	12.71
107	2.72	2.99	-	3.27	3.67	3.65	3.31	4.71	2.26	1.97	1.42	2.16	1.78	1.89	4.02	-	1.78	-	-	-	-	12.71
108	2.71	2.99	-	3.27	3.67	3.66	3.31	4.76	2.22	1.97	1.42	2.16	1.78	1.89	4.02	-	1.78	-	-	-	-	12.71
109	2.72	2.98	-	3.27	3.68	3.65	3.31	4.81	2.16	1.97	1.42	2.16	1.78	1.89	4.03	-	1.79	-	-	-	-	12.72
110	2.68	3.02	-	3.27	3.69	3.66	3.31	4.88	2.10	1.98	1.42	2.16	1.78	1.89	4.03	-	1.78	-	-	-	-	12.72
111	2.66	3.03	-	3.28	3.67	3.66	3.31	4.93	2.04	1.99	1.42	2.16	1.78	1.89	4.02	-	1.79	-	-	-	-	12.72
112	2.64	3.04	-	3.27	3.69	3.66	3.31	4.96	2.00	1.98	1.41	2.16	1.78	1.89	4.02	-	1.80	-	-	-	-	12.72
113	2.67	3.01	-	3.69	3.27	3.67	3.31	1.93	5.03	1.99	1.41	2.16	1.78	1.89	4.02	-	1.79	-	-	-	-	12.73
114	2.65	3.03	-	3.69	3.27	3.66	3.31	1.88	5.09	1.98	1.40	2.16	1.78	1.89	4.03	-	1.80	-	-	-	-	12.73
115	2.61	3.06	-	3.69	3.27	3.66	3.32	1.82	5.14	1.98	1.40	2.16	1.78	1.89	4.03	-	1.80	-	-	-	-	12.73
116	2.58	3.09	-	3.25	3.71	3.65	3.31	1.75	5.19	1.98	1.40	2.16	1.78	1.89	4.04	-	1.80	-	-	-	-	12.73
117	2.62	3.07	-	3.24	3.71	3.66	3.31	1.70	5.23	1.97	1.40	2.16	1.78	1.89	4.03	-	1.80	-	-	-	-	12.73
118	2.59	3.09	-	3.69	3.26	3.65	3.32	1.64	5.30	1.98	1.40	2.16	1.78	1.89	4.04	-	1.81	-	-	-	-	12.74
119	2.56	3.11	-	3.68	3.26	3.66	3.31	1.59	5.37	1.98	1.40	2.16	1.78	1.89	4.03	-	1.81	-	-	-	-	12.74
120	2.59	3.07	-	3.24	3.70	3.65	3.31	1.53	5.43	1.97	1.40	2.16	1.78	1.89	4.04	-	1.81	-	-	-	-	12.74
121	5.65	-	-	3.26	3.69	3.66	3.31	1.46	5.50	1.97	1.40	2.16	1.78	1.89	4.05	-	1.81	-	-	-	-	12.74
122	5.64	-	-	3.26	3.69	3.66	3.31	5.54	1.42	1.97	1.40	2.15	1.78	1.88	4.06	-	1.82	-	-	0.18	-	12.75
123	5.64	-	-	3.25	3.71	3.66	3.30	5.60	1.35	1.96	1.41	2.15	1.78	1.88	4.07	-	1.82	-	-	0.18	-	12.75
124	5.64	-	-	3.24	3.71	3.66	3.29	5.65	1.29	1.96	1.40	2.15	1.78	1.88	4.07	-	1.83	-	-	0.18	-	12.75
125	5.64	-	-	3.23	3.72	3.66	3.30	5.70	1.24	1.95	1.40	2.14	1.78	1.88	4.09	-	1.83	-	-	0.18	-	12.75
126	5.64	-	-	3.23	3.72	3.67	3.30	5.76	1.18	1.95	1.39	2.14	1.78	1.88	4.10	-	1.83	-	-	0.18	-	12.76
127	5.64	-	-	3.23	3.72	3.67	3.29	5.81	1.13	1.95	1.39	2.14	1.78	1.89	4.11	-	1.83	-	-	0.18	-	12.76
128	5.63	-	-	3.72	3.24	3.30	3.67	1.07	5.86	1.94	1.39	2.14	1.77	1.89	4.12	-	1.83	-	-	0.19	-	12.76
129	5.62	-	-	3.24	3.72	3.67	3.30	5.90	1.02	1.94	1.39	2.14	1.78	1.89	4.12	-	1.83	-	-	0.19	-	12.76
130	5.62	-	-	3.22	3.73	3.67	3.30	5.95	0.96	1.93	1.39	2.13	1.78	1.89	4.14	-	1.83	-	-	0.20	-	12.77
131	5.62	-	-	3.23	3.73	3.68	3.29	6.00	0.93	1.93	1.38	2.13	1.77	1.88	4.14	-	1.84	-	-	0.20	-	12.77
132	5.62	-	-	3.21	3.74	3.67	3.29	6.06	0.87	1.92	1.38	2.13	1.77	1.89	4.15	-	1.84	-	-	0.21	-	12.77

133	5.60	-	-	3.74	3.21	3.67	3.29	0.83	6.10	1.91	1.38	2.12	1.77	1.89	4.16	-	1.85	-	-	0.21	-	12.77
134	5.60	-	-	3.23	3.74	3.29	3.67	6.14	0.78	1.91	1.38	2.12	1.78	1.89	4.16	-	1.85	-	-	0.21	-	12.78
135	5.60	-	-	3.21	3.75	3.28	3.68	6.17	0.74	1.90	1.38	2.11	1.78	1.89	4.18	-	1.85	-	-	0.22	-	12.78
136	5.61	-	-	3.75	3.20	3.68	3.27	0.71	6.21	1.90	1.37	2.11	1.77	1.89	4.19	-	1.85	-	-	0.23	-	12.78
137	5.60	-	-	3.75	3.20	3.68	3.28	0.67	6.22	1.89	1.37	2.10	1.78	1.89	4.20	-	1.85	-	-	0.23	-	12.79
138	5.60	-	-	3.76	3.20	3.68	3.27	0.65	6.25	1.88	1.37	2.10	1.78	1.89	4.21	-	1.85	-	-	0.24	-	12.79
139	5.61	-	-	3.18	3.77	3.69	3.28	6.26	0.64	1.87	1.37	2.09	1.78	1.89	4.22	-	1.85	-	-	0.24	-	12.79
140	5.60	-	-	3.17	3.78	3.69	3.28	6.27	0.63	1.87	1.37	2.09	1.78	1.89	4.23	-	1.85	-	-	0.25	-	12.79
141	5.59	-	-	3.77	3.17	3.69	3.27	0.61	6.29	1.85	1.36	2.09	1.78	1.89	4.25	-	1.85	-	-	0.25	-	12.79
142	5.59	-	-	3.77	3.17	3.70	3.26	0.59	6.31	1.84	1.36	2.09	1.78	1.88	4.27	-	1.84	-	-	0.26	-	12.79
143	5.58	-	-	3.78	3.16	3.70	3.25	0.58	6.32	1.84	1.35	2.08	1.78	1.88	4.28	-	1.85	-	-	0.27	-	12.8
144	5.59	-	-	3.78	3.16	3.71	3.25	0.56	6.33	1.83	1.35	2.08	1.78	1.88	4.29	-	1.85	-	-	0.27	-	12.8
145	5.58	-	-	3.80	3.15	3.71	3.24	0.55	6.34	1.82	1.35	2.07	1.78	1.88	4.30	-	1.86	-	-	0.28	-	12.8
146	5.58	-	-	3.81	3.14	3.71	3.24	0.55	6.34	1.81	1.35	2.07	1.78	1.88	4.31	-	1.86	-	-	0.28	-	12.81
147	5.58	-	-	3.82	3.13	3.72	3.24	0.54	6.35	1.79	1.34	2.06	1.78	1.88	4.33	-	1.86	-	-	0.29	-	12.81
148	5.57	-	-	3.82	3.12	3.72	3.23	0.54	6.35	1.78	1.34	2.06	1.78	1.89	4.35	-	1.86	-	-	0.30	-	12.81
149	5.57	-	-	3.11	3.84	3.72	3.23	6.34	0.54	1.77	1.34	2.06	1.78	1.88	4.36	-	1.85	-	-	0.30	-	12.81
150	5.58	-	-	3.12	3.82	3.73	3.22	6.34	0.54	1.76	1.34	2.06	1.78	1.88	4.38	-	1.85	-	-	0.31	-	12.81
151	5.27	-	-	3.13	3.81	3.74	3.21	6.33	0.55	1.75	1.34	2.05	1.78	1.88	4.40	-	1.85	-	-	0.32	-	12.82
152	5.59	-	-	3.13	3.81	3.76	3.20	6.31	0.58	1.75	1.33	2.05	1.78	1.88	4.41	-	1.86	-	-	0.26	-	12.82
153	5.59	-	-	6.93	-	3.76	3.19	6.34	0.59	1.74	1.33	2.04	1.78	1.88	4.42	-	1.86	-	-	0.25	-	12.82
154	5.58	-	-	6.93	-	3.77	3.16	6.34	0.60	1.72	1.34	2.04	1.78	1.88	4.43	-	1.86	-	-	0.24	-	12.82
155	5.58	-	-	6.93	-	3.17	3.78	6.32	0.64	1.71	1.33	2.04	1.78	1.88	4.44	-	1.86	-	-	0.22	-	12.83
156	5.60	-	-	6.93	-	3.16	3.79	6.30	0.66	1.71	1.33	2.03	1.78	1.88	4.45	-	1.85	-	-	0.21	-	12.82
157	5.58	-	-	6.93	-	3.16	3.79	6.29	0.71	1.70	1.33	2.03	1.78	1.88	4.46	-	1.85	-	-	0.18	-	12.83
158	5.58	-	-	6.93	-	3.15	3.79	6.24	-	1.68	1.33	2.02	1.78	1.88	4.48	-	1.86	-	0.97	-	-	12.83
159	5.58	-	-	6.93	-	3.14	3.80	6.22	-	1.67	1.33	2.02	1.78	1.88	4.49	-	1.86	-	0.99	-	-	12.83
160	5.58	-	-	6.92	-	3.14	3.80	6.21	-	1.64	1.31	2.01	1.78	1.88	2.76	1.77	1.86	-	1.01	-	-	12.83
161	5.57	-	-	6.91	-	3.81	3.13	6.21	-	1.63	1.31	2.01	1.78	1.88	2.74	1.80	1.86	-	1.03	-	-	12.84
162	5.57	-	-	6.92	-	3.82	3.12	6.19	-	1.62	1.31	2.00	1.78	1.87	2.70	1.86	1.86	-	1.05	-	-	12.84
163	5.56	-	-	6.92	-	3.82	3.11	6.19	-	1.61	1.31	2.00	1.78	1.88	2.67	1.90	1.86	-	1.07	-	-	12.84
164	5.56	-	-	6.91	-	3.82	3.12	6.16	-	1.60	1.31	1.99	1.78	1.88	2.64	1.94	1.86	-	1.09	-	-	12.84
165	5.56	-	-	6.92	-	3.82	3.12	6.13	-	1.58	1.31	1.99	1.78	1.87	2.62	1.98	1.86	-	1.12	-	-	12.84
166	5.56	-	-	6.92	-	3.83	3.11	6.11	-	1.57	1.31	1.99	1.78	1.88	2.61	2.01	1.86	-	1.14	-	-	12.84
167	5.56	-	-	6.92	-	3.83	3.10	6.09	-	1.56	1.31	1.98	1.78	1.87	2.60	2.04	1.86	-	1.17	-	-	12.84

168	5.55	-	-	6.91	-	6.92	-	6.07	-	1.55	1.31	1.98	1.78	1.87	2.58	2.07	1.87	-	1.20	-	-	12.85
169	5.55	-	-	6.91	-	6.92	-	6.05	-	1.53	1.30	1.98	1.78	1.87	2.57	2.09	1.87	-	1.22	-	-	12.85
170	5.54	-	-	6.92	-	6.92	-	6.03	-	1.52	1.30	1.97	1.78	1.87	2.56	2.11	1.87	-	1.24	-	-	12.85
171	5.55	-	-	6.91	-	6.92	-	6.01	-	1.51	1.31	1.97	1.78	1.87	2.55	2.13	1.87	-	1.28	-	-	12.85
172	5.55	-	-	6.91	-	6.93	-	5.99	-	1.50	1.30	1.97	1.78	1.87	2.55	2.15	1.86	-	1.30	-	-	12.85
173	5.55	-	-	6.91	-	6.92	-	5.97	-	1.49	1.30	1.96	1.78	1.87	2.54	2.16	1.86	-	1.33	-	-	12.85
174	5.54	-	-	6.91	-	6.92	-	5.96	-	1.49	1.30	1.96	1.79	1.87	2.54	2.17	1.87	-	1.34	-	-	12.85
175	5.55	-	-	6.91	-	6.92	-	5.94	-	1.49	1.31	1.95	1.79	1.87	2.53	2.17	1.87	-	1.37	-	-	12.85
176	5.54	-	-	6.91	-	3.84	3.08	5.92	-	1.48	1.31	1.95	1.79	1.87	2.53	2.18	1.88	-	1.38	-	-	12.84
177	5.55	-	-	6.91	-	3.83	3.09	5.91	-	1.48	1.31	1.95	1.79	1.87	2.52	2.19	1.87	-	1.39	-	-	12.84
178	5.55	-	-	6.92	-	3.83	3.09	5.90	-	1.48	1.31	1.95	1.79	1.87	2.53	2.19	1.86	-	1.40	-	-	12.84
179	5.55	-	-	6.92	-	3.82	3.10	5.89	-	1.48	1.31	1.95	1.79	1.87	2.53	2.19	1.86	-	1.41	-	-	12.84
180	5.54	-	-	6.92	-	3.78	3.14	5.89	-	1.47	1.30	1.95	1.79	1.87	2.53	2.20	1.87	-	1.42	-	-	12.83
181	5.55	-	-	6.92	-	3.77	3.16	5.88	-	1.47	1.31	1.95	1.79	1.87	2.52	2.21	1.87	-	1.42	-	-	12.83
182	5.55	-	-	6.93	-	3.74	3.19	5.88	-	1.47	1.31	1.95	1.79	1.87	2.52	2.21	1.86	-	1.43	-	-	12.83
183	5.56	-	-	3.83	3.09	3.72	3.21	5.88	-	1.48	1.30	1.94	1.79	1.87	2.51	2.21	1.86	-	1.43	-	-	12.83
184	5.56	-	-	3.83	3.09	3.68	3.24	5.88	-	1.48	1.30	1.95	1.79	1.87	2.51	2.21	1.86	-	1.43	-	-	12.83
185	5.56	-	-	3.79	3.13	3.67	3.26	5.87	-	1.48	1.30	1.95	1.79	1.87	2.51	2.21	1.86	-	1.44	-	-	12.82
186	5.57	-	-	3.76	3.17	3.63	3.30	5.87	-	1.48	1.30	1.95	1.79	1.87	2.51	2.21	1.86	-	1.44	-	-	12.82
187	5.58	-	-	3.73	3.20	3.59	3.34	5.86	-	1.48	1.30	1.94	1.79	1.87	2.51	2.21	1.86	-	1.45	-	-	12.82
188	5.57	-	-	3.69	3.23	3.58	3.34	5.87	-	1.48	1.30	1.95	1.79	1.87	2.51	2.21	1.85	-	1.45	-	-	12.82
189	5.57	-	-	3.66	3.26	3.55	3.37	5.87	-	1.48	1.30	1.95	1.79	1.87	2.52	2.21	1.85	-	1.45	-	-	12.82
190	5.56	-	-	3.63	3.30	3.53	3.40	5.87	-	1.48	1.30	1.95	1.79	1.87	2.51	2.22	1.86	-	1.45	-	-	12.82
191	5.57	-	-	3.60	3.32	3.51	3.43	5.86	-	1.48	1.31	1.95	1.79	1.87	2.50	2.22	1.86	-	1.45	-	-	12.81
192	5.58	-	-	3.57	3.36	3.49	3.44	5.86	-	1.48	1.30	1.95	1.79	1.87	2.50	2.21	1.86	-	1.45	-	-	12.81
193	5.59	-	-	3.55	3.38	3.46	3.46	5.85	-	1.48	1.30	1.95	1.79	1.87	2.51	2.21	1.85	-	1.45	-	-	12.81
194	5.58	-	-	3.51	3.41	3.44	3.48	5.86	-	1.47	1.31	1.95	1.79	1.87	2.51	2.22	1.85	-	1.45	-	-	12.81
195	5.58	-	-	3.48	3.44	3.40	3.51	5.85	-	1.47	1.31	1.95	1.79	1.87	2.50	2.22	1.85	-	1.46	-	-	12.81
196	5.58	-	-	3.46	3.46	3.37	3.53	5.85	-	1.47	1.31	1.95	1.79	1.87	2.50	2.21	1.86	-	1.46	-	-	12.81
197	5.59	-	-	3.48	3.43	3.35	3.56	5.84	-	1.48	1.31	1.95	1.79	1.87	2.22	2.50	1.85	-	1.46	-	-	12.81
198	5.59	-	-	3.41	3.50	3.34	3.56	5.85	-	1.48	1.31	1.95	1.79	1.87	2.22	2.50	1.84	-	1.46	-	-	12.81
199	5.59	-	-	3.40	3.52	3.31	3.58	5.85	-	1.48	1.31	1.95	1.79	1.87	2.21	2.50	1.84	-	1.47	-	-	12.81
200	5.60	-	-	3.37	3.54	3.30	3.61	5.84	-	1.48	1.31	1.95	1.79	1.87	2.21	2.51	1.85	-	1.47	-	-	12.81
201	5.59	-	-	3.36	3.56	3.29	3.63	5.84	-	1.48	1.31	1.95	1.79	1.87	2.21	2.50	1.85	-	1.47	-	-	12.81
202	5.58	-	-	3.34	3.56	3.26	3.64	5.84	-	1.48	1.31	1.95	1.79	1.87	2.22	2.50	1.86	-	1.47	-	-	12.81

203	5.57	-	-	3.33	3.57	3.26	3.64	5.84	-	1.48	1.32	1.95	1.79	1.88	2.22	2.50	1.86	-	1.47	-	-	12.81
204	5.57	-	-	3.32	3.59	3.25	3.65	5.83	-	1.47	1.32	1.95	1.79	1.87	2.22	2.50	1.86	-	1.48	-	-	12.81
205	5.58	-	-	3.32	3.60	3.24	3.66	5.83	-	1.47	1.32	1.95	1.79	1.87	2.22	2.50	1.86	-	1.48	-	-	12.81
206	5.58	-	-	3.31	3.61	3.24	3.67	5.84	-	1.47	1.32	1.94	1.79	1.87	2.23	2.50	1.85	-	1.48	-	-	12.81
207	5.58	-	-	3.30	3.61	3.25	3.68	5.83	-	1.48	1.32	1.95	1.79	1.87	2.23	2.50	1.85	-	1.48	-	-	12.81
208	5.59	-	-	3.30	3.62	3.25	3.67	5.83	-	1.47	1.32	1.95	1.79	1.87	2.24	2.49	1.85	-	1.48	-	-	12.81
209	5.59	-	-	3.30	3.62	3.25	3.69	5.83	-	1.47	1.31	1.94	1.79	1.87	2.23	2.49	1.85	-	1.48	-	-	12.81
210	5.59	-	-	3.28	3.63	3.25	3.69	5.83	-	1.47	1.31	1.94	1.79	1.87	2.23	2.49	1.85	-	1.48	-	-	12.81
211	5.59	-	-	3.29	3.64	3.24	3.70	5.83	-	1.47	1.31	1.94	1.79	1.87	2.24	2.49	1.85	-	1.48	-	-	12.81
212	5.60	-	-	3.29	3.64	3.24	3.70	5.83	-	1.47	1.31	1.95	1.79	1.87	2.24	2.48	1.84	-	1.48	-	-	12.81
213	5.59	-	-	3.29	3.64	3.25	3.68	5.83	-	1.47	1.31	1.95	1.78	1.87	2.24	2.48	1.85	-	1.48	-	-	12.81
214	5.59	-	-	3.28	3.64	3.25	3.69	5.84	-	1.47	1.31	1.94	1.78	1.88	2.24	2.47	1.85	-	1.47	-	-	12.81
215	5.58	-	-	3.29	3.64	3.25	3.69	5.83	-	1.47	1.31	1.94	1.78	1.88	2.24	2.47	1.85	-	1.47	-	-	12.81
216	5.58	-	-	3.29	3.64	3.24	3.69	5.82	-	1.47	1.31	1.94	1.78	1.87	2.25	2.47	1.86	-	1.47	-	-	12.82
217	5.59	-	-	3.28	3.64	3.24	3.70	5.82	-	1.47	1.31	1.94	1.78	1.87	2.24	2.46	1.85	-	1.47	-	-	12.82
218	5.59	-	-	3.28	3.64	3.24	3.69	5.82	-	1.47	1.31	1.94	1.78	1.87	2.25	2.46	1.85	-	1.47	-	-	12.81
219	5.59	-	-	3.28	3.65	3.24	3.69	5.82	-	1.47	1.31	1.94	1.78	1.87	2.26	2.46	1.84	-	1.47	-	-	12.82
220	5.59	-	-	3.27	3.65	3.24	3.68	5.83	-	1.47	1.31	1.95	1.78	1.87	2.27	2.46	1.85	-	1.47	-	-	12.82
221	5.59	-	-	3.27	3.65	3.23	3.69	5.82	-	1.47	1.31	1.95	1.78	1.87	2.27	2.45	1.85	-	1.48	-	-	12.82
222	5.58	-	-	3.28	3.64	3.23	3.70	5.83	-	1.47	1.31	1.95	1.78	1.87	2.27	2.45	1.85	-	1.48	-	-	12.82
223	5.58	-	-	3.27	3.65	3.25	3.69	5.82	-	1.47	1.31	1.95	1.78	1.88	2.28	2.45	1.84	-	1.48	-	-	12.82
224	5.57	-	-	3.28	3.64	3.25	3.68	5.82	-	1.47	1.30	1.95	1.80	1.88	2.28	2.45	1.84	-	1.48	-	-	12.82
225	5.58	-	-	3.28	3.64	3.25	3.69	5.82	-	1.47	1.30	1.95	1.80	1.88	2.29	2.45	1.83	-	1.48	-	-	12.82
226	5.59	-	-	3.28	3.63	3.24	3.69	5.82	-	1.47	1.30	1.95	1.80	1.88	2.29	2.45	1.84	-	1.48	-	-	12.82
227	5.60	-	-	3.28	3.63	3.24	3.70	5.82	-	1.47	1.31	1.94	1.80	1.88	2.29	2.44	1.84	-	1.48	-	-	12.82
228	5.60	-	-	3.28	3.63	3.25	3.69	5.82	-	1.47	1.31	1.94	1.80	1.88	2.29	2.44	1.85	-	1.48	-	-	12.82
229	5.60	-	-	3.28	3.64	3.25	3.69	5.81	-	1.48	1.31	1.94	1.80	1.88	2.29	2.44	1.84	-	1.48	-	-	12.81
230	5.59	-	-	3.64	3.27	3.24	3.70	5.81	-	1.48	1.31	1.94	1.79	1.88	2.30	2.43	1.84	-	1.48	-	-	12.82
231	5.59	-	-	3.64	3.27	3.23	3.70	5.79	-	1.48	1.30	1.95	1.79	1.88	2.30	2.44	1.84	-	1.48	-	-	12.81
232	5.59	-	-	3.64	3.27	3.24	3.70	5.78	-	1.48	1.30	1.95	1.79	1.87	2.30	2.44	1.84	-	1.48	-	-	12.81
233	5.59	-	-	3.64	3.27	3.25	3.70	5.79	-	1.47	1.30	1.95	1.79	1.87	2.30	2.44	1.85	-	1.48	-	-	12.81
234	5.59	-	-	3.64	3.28	3.24	3.69	5.81	-	1.46	1.30	1.95	1.79	1.87	2.31	2.44	1.84	-	1.48	-	-	12.81
235	5.58	-	-	3.64	3.28	3.25	3.69	5.82	-	1.45	1.30	1.94	1.79	1.87	2.32	2.44	1.85	-	1.48	-	-	12.81
236	5.59	-	-	3.63	3.29	3.25	3.69	5.83	-	1.46	1.30	1.95	1.79	1.88	2.32	2.44	1.85	-	1.48	-	-	12.81
237	5.59	-	-	3.64	3.29	3.25	3.69	5.83	-	1.46	1.30	1.95	1.79	1.88	2.32	2.43	1.85	-	1.48	-	-	12.81

238	5.59	-	-	3.65	3.28	3.24	3.70	5.83	-	1.46	1.30	1.95	1.79	1.88	2.32	2.43	1.85	-	1.47	-	-	12.81
239	5.58	-	-	3.65	3.28	3.25	3.69	5.83	-	1.46	1.30	1.95	1.79	1.88	2.32	2.43	1.85	-	1.47	-	-	12.82
240	5.58	-	-	3.65	3.28	3.24	3.69	5.82	-	1.46	1.30	1.95	1.79	1.88	2.32	2.43	1.85	-	1.48	-	-	12.81
241	5.58	-	-	3.65	3.28	3.24	3.69	5.81	-	1.46	1.30	1.95	1.79	1.88	2.33	2.43	1.85	-	1.48	-	-	12.81
242	5.57	-	-	3.65	3.27	3.23	3.70	5.79	-	1.46	1.30	1.95	1.79	1.87	2.33	2.42	1.85	-	1.48	-	-	12.81
243	5.56	-	-	3.66	3.27	3.24	3.69	5.81	-	1.46	1.30	1.94	1.79	1.87	2.33	2.42	1.84	-	1.48	-	-	12.82
244	5.57	-	-	3.65	3.27	3.25	3.68	5.81	-	1.46	1.30	1.95	1.79	1.88	2.34	2.41	1.85	-	1.48	-	-	12.82
245	5.57	-	-	3.65	3.27	3.25	3.68	5.81	-	1.46	1.30	1.95	1.79	1.88	2.33	2.42	1.85	-	1.48	-	-	12.82
246	5.58	-	-	3.65	3.27	3.25	3.68	5.82	-	1.46	1.30	1.95	1.79	1.88	2.33	2.41	1.84	-	1.48	-	-	12.82
247	5.59	-	-	3.64	3.28	3.24	3.68	5.82	-	1.45	1.30	1.95	1.79	1.87	2.34	2.41	1.84	-	1.48	-	-	12.82
248	5.58	-	-	3.64	3.27	3.23	3.69	5.82	-	1.45	1.31	1.95	1.80	1.88	2.34	2.40	1.85	-	1.48	-	-	12.82
249	5.58	-	-	3.27	3.65	3.23	3.68	5.82	-	1.45	1.31	1.94	1.80	1.88	2.40	2.34	1.85	-	1.48	-	-	12.82
250	5.58	-	-	3.27	3.65	3.24	3.68	5.81	-	1.45	1.30	1.94	1.80	1.88	2.34	2.41	1.85	-	1.48	-	-	12.82
251	5.58	-	-	3.27	3.64	3.25	3.68	5.79	-	1.46	1.31	1.94	1.79	1.88	2.34	2.40	1.85	-	1.48	-	-	12.82
252	5.57	-	-	3.27	3.65	3.25	3.69	5.79	-	1.46	1.30	1.94	1.79	1.88	2.34	2.41	1.86	-	1.48	-	-	12.82
253	5.58	-	-	3.26	3.65	3.26	3.68	5.81	-	1.46	1.30	1.94	1.79	1.88	2.34	2.41	1.85	-	1.48	-	-	12.82
254	5.58	-	-	3.26	3.65	3.26	3.67	5.81	-	1.46	1.30	1.94	1.79	1.88	2.34	2.40	1.85	-	1.48	-	-	12.82
255	5.58	-	-	3.26	3.66	3.26	3.68	5.82	-	1.46	1.30	1.94	1.79	1.88	2.35	2.40	1.85	-	1.49	-	-	12.82
256	5.58	-	-	3.26	3.65	3.26	3.68	5.82	-	1.46	1.30	1.95	1.79	1.87	2.34	2.41	1.85	-	1.49	-	-	12.82
257	5.58	-	-	3.26	3.65	3.26	3.67	5.82	-	1.46	1.30	1.94	1.79	1.87	2.34	2.41	1.85	-	1.49	-	-	12.82
258	5.59	-	-	3.26	3.65	3.26	3.67	5.83	-	1.46	1.30	1.95	1.79	1.87	2.35	2.42	1.84	-	1.48	-	-	12.82
259	5.58	-	-	3.27	3.65	3.25	3.68	5.81	-	1.45	1.30	1.95	1.79	1.87	2.35	2.42	1.85	-	1.48	-	-	12.82
260	5.58	-	-	3.27	3.65	3.26	3.68	5.81	-	1.45	1.30	1.95	1.79	1.87	2.35	2.41	1.85	-	1.48	-	-	12.82
261	5.59	-	-	3.27	3.65	3.26	3.68	5.79	-	1.45	1.29	1.95	1.79	1.87	2.36	2.40	1.84	-	1.48	-	-	12.82
262	5.58	-	-	3.27	3.65	3.26	3.68	5.79	-	1.45	1.29	1.95	1.79	1.88	2.36	2.40	1.85	-	1.48	-	-	12.82
263	5.58	-	-	3.26	3.66	3.26	3.68	5.81	-	1.45	1.29	1.95	1.79	1.88	2.37	2.40	1.85	-	1.48	-	-	12.82
264	5.58	-	-	3.27	3.66	3.26	3.68	5.81	-	1.45	1.30	1.95	1.79	1.88	2.36	2.40	1.86	-	1.49	-	-	12.82

Table S13. ELF basin populations for the oxidation of **R3**

	V1(O3)	V2(O3)	V3(O3)	V1(O4)	V2(O4)	V1(O5)	V2(O5)	V1(O6)	V2(O6)	V3(O6)	V(C1,S10)	V(C9,S10)	V(C1,C2)	V(C2,C8)	V(C8,C9)	V1(S10)	V2(S10)	V(O3,H)	V(C1,H)	V(C1,O6)	V(C1)	V(H)	Ru
1	6.87	-	-	6.85	-	6.87	-	6.91	-	-	1.52	1.51	1.83	1.81	1.84	2.22	2.29	-	2.05	-	-	-	12.17
2	6.87	-	-	6.85	-	6.87	-	6.91	-	-	1.52	1.51	1.83	1.81	1.84	2.22	2.29	-	2.05	-	-	-	12.17
3	6.87	-	-	6.86	-	6.88	-	6.91	-	-	1.52	1.51	1.83	1.81	1.84	2.22	2.29	-	2.05	-	-	-	12.17
4	6.87	-	-	6.85	-	6.88	-	6.91	-	-	1.53	1.51	1.83	1.81	1.84	2.22	2.29	-	2.05	-	-	-	12.17
5	6.86	-	-	6.86	-	6.87	-	6.91	-	-	1.52	1.51	1.83	1.81	1.84	2.21	2.29	-	2.05	-	-	-	12.17
6	6.86	-	-	6.86	-	6.87	-	6.92	-	-	1.53	1.51	1.83	1.81	1.84	2.21	2.29	-	2.05	-	-	-	12.18
7	6.85	-	-	6.87	-	6.87	-	6.92	-	-	1.53	1.51	1.83	1.81	1.84	2.21	2.29	-	2.05	-	-	-	12.18
8	6.86	-	-	6.87	-	6.87	-	6.92	-	-	1.53	1.51	1.83	1.81	1.84	2.21	2.29	-	2.05	-	-	-	12.18
9	6.85	-	-	6.87	-	6.87	-	6.91	-	-	1.53	1.51	1.83	1.81	1.84	2.21	2.28	-	2.04	-	-	-	12.18
10	6.85	-	-	6.87	-	6.88	-	6.91	-	-	1.53	1.51	1.83	1.81	1.84	2.28	2.21	-	2.04	-	-	-	12.18
11	6.85	-	-	6.87	-	6.88	-	6.91	-	-	1.54	1.51	1.83	1.81	1.84	2.28	2.20	-	2.04	-	-	-	12.19
12	6.85	-	-	6.87	-	6.89	-	6.92	-	-	1.54	1.51	1.83	1.82	1.84	2.28	2.20	-	2.03	-	-	-	12.19
13	6.85	-	-	6.88	-	6.89	-	6.92	-	-	1.54	1.51	1.83	1.81	1.84	2.28	2.20	-	2.03	-	-	-	12.19
14	6.85	-	-	6.88	-	6.89	-	4.84	2.08	-	1.54	1.51	1.83	1.81	1.84	2.28	2.20	-	2.03	-	-	-	12.19
15	6.85	-	-	6.88	-	6.89	-	4.69	2.23	-	1.55	1.51	1.84	1.81	1.84	2.20	2.28	-	2.03	-	-	-	12.19
16	6.84	-	-	6.87	-	6.89	-	4.63	2.29	-	1.55	1.51	1.83	1.81	1.84	2.19	2.27	-	2.02	-	-	-	12.19
17	6.84	-	-	6.87	-	6.89	-	4.55	2.36	-	1.55	1.51	1.83	1.81	1.84	2.19	2.28	-	2.02	-	-	-	12.20
18	6.84	-	-	3.68	3.18	6.90	-	4.48	2.43	-	1.55	1.51	1.84	1.81	1.83	2.19	2.27	-	2.01	-	-	-	12.20
19	6.85	-	-	3.60	3.26	4.01	2.88	4.40	2.51	-	1.55	1.51	1.83	1.81	1.84	2.18	2.27	-	2.01	-	-	-	12.20
20	6.84	-	-	3.53	3.34	3.91	2.99	4.32	2.59	-	1.55	1.51	1.84	1.81	1.84	2.18	2.27	-	2.01	-	-	-	12.20
21	6.85	-	-	3.52	3.36	3.84	3.05	2.64	4.28	-	1.56	1.51	1.83	1.81	1.84	2.18	2.27	-	2.00	-	-	-	12.21
22	6.84	-	-	3.52	3.37	3.78	3.11	2.72	4.20	-	1.56	1.51	1.84	1.81	1.84	2.18	2.27	-	2.00	-	-	-	12.21
23	1.81	-	-	3.53	3.36	3.75	3.14	2.79	4.13	-	1.57	1.51	1.84	1.81	1.84	2.17	2.26	-	1.99	-	-	-	12.21
24	6.84	-	-	3.54	3.35	3.73	3.16	2.86	4.05	-	1.57	1.50	1.84	1.81	1.84	2.17	2.26	-	1.99	-	-	-	12.22
25	6.84	-	-	3.55	3.35	3.72	3.18	2.92	4.00	-	1.58	1.50	1.84	1.81	1.84	2.17	2.26	-	1.98	-	-	-	12.22
26	6.85	-	-	3.56	3.34	3.71	3.18	2.98	3.93	-	1.58	1.51	1.84	1.81	1.84	2.16	2.26	-	1.97	-	-	-	12.22
27	6.85	-	-	3.57	3.34	3.70	3.20	3.04	3.88	-	1.58	1.51	1.84	1.81	1.84	2.16	2.26	-	1.96	-	-	-	12.22
28	6.85	-	-	3.58	3.32	3.68	3.21	3.07	3.85	-	1.59	1.51	1.84	1.81	1.84	2.15	2.25	-	1.96	-	-	-	12.23

29	6.85	-	-	3.60	3.30	3.69	3.22	3.11	3.80	-	1.60	1.51	1.84	1.81	1.84	2.14	2.25	-	1.95	-	-	-	12.24
30	6.85	-	-	3.63	3.28	3.70	3.20	3.15	3.77	-	1.60	1.51	1.84	1.81	1.84	2.14	2.25	-	1.93	-	-	-	12.24
31	3.69	3.17	-	3.62	3.28	3.70	3.20	3.17	3.74	-	1.61	1.51	1.84	1.81	1.84	2.13	2.25	-	1.92	-	-	-	12.25
32	3.65	3.21	-	3.64	3.26	3.71	3.20	3.20	3.72	-	1.61	1.51	1.84	1.81	1.84	2.13	2.25	-	1.91	-	-	-	12.26
33	3.60	3.26	-	3.65	3.24	3.72	3.19	3.22	3.70	-	1.62	1.51	1.84	1.81	1.84	2.12	2.25	-	1.91	-	-	-	12.27
34	3.56	3.30	-	3.68	3.21	3.74	3.17	3.23	3.68	-	1.64	1.51	1.85	1.81	1.84	2.11	2.25	-	1.89	-	-	-	12.28
35	3.54	3.33	-	3.72	3.18	3.77	3.15	3.24	3.67	-	1.65	1.51	1.85	1.81	1.84	2.09	2.25	-	1.85	-	-	-	12.30
36	3.52	3.34	-	3.76	3.13	3.81	3.11	3.27	3.65	-	1.66	1.51	1.85	1.81	1.84	2.08	2.24	-	1.82	-	-	-	12.32
37	3.47	3.39	-	3.80	3.10	3.83	3.09	3.28	3.65	-	1.69	1.51	1.86	1.81	1.84	2.06	2.25	-	1.78	-	-	-	12.34
38	3.39	3.15	0.32	3.83	3.08	3.85	3.08	3.30	3.64	-	1.71	1.51	1.87	1.81	1.84	2.03	2.25	-	1.72	-	-	-	12.37
39	3.34	3.05	0.45	3.83	3.09	3.85	3.08	3.31	3.65	-	1.73	1.51	1.89	1.81	1.84	2.01	2.26	-	1.65	-	-	-	12.40
40	2.93	3.29	0.59	3.82	3.09	3.82	3.11	3.29	3.66	-	1.76	1.52	1.90	1.81	1.84	1.98	2.26	-	-	-	0.89	0.70	12.45
41	6.08	-	0.70	3.80	3.12	3.80	3.14	3.28	3.68	-	1.79	1.52	1.91	1.81	1.84	1.95	2.27	-	-	-	0.83	0.70	12.48
42	6.00	-	-	3.78	3.15	3.78	3.16	3.28	3.67	-	1.83	1.52	1.93	1.81	1.84	1.93	2.27	1.47	-	-	0.77	-	12.50
43	5.95	-	-	3.76	3.18	3.77	3.17	3.28	3.68	-	1.86	1.52	1.94	1.81	1.84	1.90	2.28	1.51	-	-	0.71	-	12.52
44	5.92	-	-	3.74	3.20	3.75	3.19	3.28	3.68	-	1.89	1.52	1.95	1.81	1.84	1.88	2.29	1.55	-	-	0.66	-	12.54
45	5.90	-	-	3.73	3.21	3.74	3.21	3.28	3.69	-	1.92	1.52	1.96	1.81	1.84	1.86	2.29	1.57	-	-	0.61	-	12.56
46	2.77	3.11	-	3.70	3.23	3.73	3.22	3.26	3.71	-	1.94	1.52	1.97	1.81	1.84	1.84	2.30	1.59	-	-	0.57	-	12.57
47	2.75	3.13	-	3.69	3.24	3.72	3.22	3.26	3.71	-	1.96	1.52	1.97	1.81	1.84	1.83	2.30	1.60	-	-	0.54	-	12.58
48	2.74	3.12	-	3.67	3.26	3.70	3.24	3.25	3.72	-	1.98	1.52	1.98	1.81	1.84	2.30	1.82	1.62	-	-	0.51	-	12.58
49	2.72	3.12	-	3.66	3.27	3.70	3.25	3.24	3.72	-	2.00	1.52	1.98	1.81	1.84	1.81	2.31	1.63	-	-	0.48	-	12.59
50	2.73	3.11	-	3.66	3.28	3.70	3.26	3.22	3.73	-	2.02	1.52	1.99	1.81	1.84	1.80	2.31	1.64	-	-	0.45	-	12.60
51	2.74	3.09	-	3.65	3.29	3.69	3.26	3.19	3.74	-	2.03	1.52	1.99	1.81	1.85	1.79	2.31	1.65	-	-	0.43	-	12.60
52	2.73	3.09	-	3.65	3.30	3.69	3.26	3.17	3.79	-	2.05	1.52	1.99	1.81	1.85	1.79	2.31	1.66	-	-	0.40	-	12.60
53	2.74	3.07	-	3.29	3.66	3.27	3.67	3.16	3.80	-	2.06	1.51	2.00	1.81	1.85	1.78	2.32	1.67	-	-	0.38	-	12.61
54	2.74	3.06	-	3.31	3.64	3.28	3.68	3.80	0.40	2.77	2.08	1.51	2.00	1.81	1.85	1.78	2.32	1.67	-	-	0.36	-	12.61
55	2.75	3.05	-	3.31	3.64	3.27	3.68	3.80	3.16	-	2.09	1.51	2.00	1.81	1.85	1.78	2.32	1.69	-	-	0.35	-	12.61
56	2.74	3.05	-	3.31	3.63	3.28	3.68	3.15	3.82	-	2.41	1.51	2.00	1.81	1.85	1.77	2.32	1.69	-	-	-	-	12.62
57	2.76	3.03	-	3.30	3.64	3.27	3.68	3.11	3.86	-	2.42	1.51	2.00	1.81	1.85	1.77	2.32	1.69	-	-	-	-	12.61
58	2.75	3.02	-	3.30	3.64	3.28	3.68	3.09	3.88	-	2.41	1.51	2.01	1.81	1.85	1.77	2.32	1.70	-	-	-	-	12.62
59	2.75	3.01	-	3.30	3.63	3.27	3.68	3.08	3.89	-	2.41	1.51	2.01	1.81	1.85	1.77	2.32	1.71	-	-	-	-	12.63
60	2.75	3.01	-	3.64	3.30	3.28	3.68	3.04	3.94	-	2.40	1.51	2.01	1.81	1.85	1.77	2.32	1.70	-	-	-	-	12.62
61	2.76	2.99	-	3.30	3.64	3.28	3.68	3.01	3.95	-	2.40	1.51	2.01	1.81	1.85	1.77	2.33	1.71	-	-	-	-	12.63
62	2.76	2.99	-	3.29	3.64	3.27	3.67	2.95	4.00	-	2.39	1.51	2.02	1.81	1.85	1.77	2.32	1.72	-	-	-	-	12.63
63	2.76	2.99	-	3.30	3.65	3.28	3.68	2.91	4.05	-	2.39	1.51	2.02	1.81	1.85	1.77	2.32	1.72	-	-	-	-	12.64

64	2.76	2.98	-	3.29	3.66	3.28	3.67	2.89	4.07	-	2.39	1.51	2.02	1.81	1.85	1.77	2.32	1.73	-	-	-	-	12.64
65	2.77	2.97	-	3.66	3.30	3.27	3.67	2.85	4.11	-	2.38	1.51	2.02	1.81	1.85	1.77	2.32	1.73	-	-	-	-	12.64
66	2.76	2.96	-	3.66	3.30	3.27	3.68	2.80	4.15	-	2.37	1.51	2.02	1.81	1.85	1.77	2.32	1.73	-	-	-	-	12.65
67	2.77	2.96	-	3.65	3.29	3.27	3.69	2.76	4.19	-	2.37	1.51	2.02	1.81	1.85	1.78	2.32	1.74	-	-	-	-	12.65
68	2.78	2.95	-	3.66	3.29	3.27	3.69	2.70	4.24	-	2.37	1.51	2.02	1.81	1.85	1.78	2.32	1.73	-	-	-	-	12.65
69	2.78	2.95	-	3.66	3.29	3.27	3.69	2.63	4.30	-	2.37	1.51	2.03	1.81	1.85	1.78	2.32	1.74	-	-	-	-	12.66
70	2.78	2.94	-	3.67	3.28	3.27	3.69	2.59	4.35	-	2.36	1.51	2.03	1.81	1.85	1.78	2.32	1.76	-	-	-	-	12.66
71	2.79	2.93	-	3.66	3.29	3.26	3.69	2.54	4.42	-	2.36	1.51	2.03	1.81	1.85	1.79	2.32	1.76	-	-	-	-	12.66
72	2.80	2.92	-	3.67	3.28	3.26	3.69	2.47	4.48	-	2.36	1.51	2.03	1.81	1.85	1.79	2.31	1.75	-	-	-	-	12.66
73	2.80	2.91	-	3.67	3.29	3.27	3.69	2.41	4.53	-	2.35	1.51	2.03	1.81	1.85	1.79	2.31	1.76	-	-	-	-	12.67
74	2.81	2.90	-	3.67	3.29	3.26	3.70	2.36	4.59	-	2.34	1.51	2.03	1.81	1.85	1.80	2.31	1.77	-	-	-	-	12.67
75	2.81	2.89	-	3.67	3.29	3.26	3.69	2.27	4.68	-	2.34	1.51	2.03	1.81	1.85	1.80	2.31	1.77	-	-	-	-	12.67
76	2.82	2.88	-	3.67	3.29	3.27	3.68	2.22	4.72	-	2.34	1.50	2.03	1.81	1.85	1.80	2.31	1.77	-	-	-	-	12.68
77	2.83	2.87	-	3.67	3.28	3.27	3.68	2.13	4.82	-	2.33	1.50	2.03	1.81	1.85	1.80	2.31	1.77	-	-	-	-	12.68
78	2.83	2.87	-	3.67	3.28	3.27	3.68	2.05	4.89	-	2.33	1.50	2.03	1.81	1.85	1.81	2.31	1.78	-	-	-	-	12.68
79	2.83	2.86	-	3.68	3.28	3.26	3.69	1.99	4.94	-	2.33	1.50	2.03	1.81	1.85	1.81	2.31	1.79	-	-	-	-	12.68
80	2.85	2.84	-	3.69	3.26	3.26	3.69	1.91	5.01	-	2.33	1.50	2.03	1.81	1.85	1.81	2.31	1.78	-	-	-	-	12.69
81	2.84	2.83	-	3.69	3.26	3.26	3.70	1.83	5.09	-	2.33	1.50	2.04	1.81	1.85	1.82	2.30	1.79	-	-	-	-	12.69
82	2.85	2.82	-	3.70	3.26	3.26	3.69	1.78	5.15	-	2.32	1.50	2.04	1.81	1.85	1.82	2.30	1.79	-	-	-	-	12.69
83	2.86	2.81	-	3.69	3.26	3.26	3.69	1.71	5.22	-	2.32	1.50	2.03	1.81	1.85	1.83	2.30	1.80	-	-	-	-	12.69
84	2.86	2.80	-	3.69	3.27	3.27	3.69	1.64	5.28	-	2.31	1.50	2.04	1.81	1.85	1.83	2.30	1.80	-	-	-	-	12.69
85	2.88	2.79	-	3.70	3.26	3.27	3.68	1.59	5.34	-	2.31	1.50	2.04	1.81	1.84	1.84	2.30	1.80	-	-	-	-	12.70
86	2.88	2.78	-	3.70	3.25	3.27	3.69	1.51	5.42	-	2.31	1.50	2.04	1.81	1.84	1.84	2.30	1.81	-	-	-	-	12.70
87	2.90	2.76	-	3.70	3.25	3.27	3.69	1.43	5.48	-	2.30	1.50	2.04	1.81	1.85	1.84	2.30	1.80	-	-	-	-	12.71
88	2.90	2.74	-	3.70	3.25	3.27	3.69	1.36	5.55	-	2.30	1.50	2.03	1.81	1.85	1.85	2.30	1.81	-	-	-	-	12.71
89	2.92	2.73	-	3.70	3.25	3.27	3.68	5.60	1.31	-	2.29	1.50	2.04	1.81	1.85	1.85	2.30	1.81	-	-	-	-	12.71
90	2.92	2.72	-	3.70	3.24	3.27	3.69	5.67	1.24	-	2.29	1.50	2.04	1.81	1.85	1.86	2.30	1.81	-	-	-	-	12.71
91	2.94	2.70	-	3.70	3.24	3.26	3.70	5.73	1.17	-	2.29	1.50	2.04	1.81	1.84	1.86	2.30	1.82	-	-	-	-	12.72
92	2.96	2.67	-	3.70	3.24	3.26	3.70	5.81	1.10	-	2.29	1.50	2.03	1.81	1.84	1.87	2.30	1.82	-	-	-	-	12.72
93	2.98	2.66	-	3.71	3.24	3.26	3.70	5.85	1.05	-	2.28	1.49	2.04	1.81	1.84	1.88	2.30	1.82	-	-	-	-	12.72
94	2.98	2.64	-	3.72	3.24	3.26	3.70	5.91	0.98	-	2.28	1.49	2.04	1.81	1.84	1.88	2.30	1.83	-	-	-	-	12.73
95	3.00	2.62	-	3.71	3.25	3.25	3.71	5.97	0.93	-	2.28	1.49	2.04	1.81	1.85	1.89	2.30	1.83	-	-	-	-	12.73
96	3.02	2.60	-	3.72	3.24	3.25	3.71	6.02	0.87	-	2.27	1.49	2.04	1.81	1.85	1.89	2.30	1.82	-	-	-	-	12.73
97	3.00	2.62	-	3.73	3.23	3.25	3.71	6.08	0.81	-	2.27	1.49	2.04	1.81	1.85	1.90	2.30	1.84	-	-	-	-	12.73
98	5.61	-	-	3.22	3.73	3.25	3.71	0.76	6.13	-	2.27	1.49	2.04	1.81	1.84	1.91	2.30	1.84	-	-	-	-	12.74

99	5.61	-	-	3.22	3.73	3.24	3.71	0.71	6.17	-	2.26	1.49	2.04	1.81	1.84	1.92	2.30	1.84	-	-	-	-	12.74
100	5.61	-	-	3.21	3.73	3.24	3.72	0.67	6.21	-	2.26	1.49	2.04	1.81	1.84	1.92	2.29	1.84	-	-	-	-	12.74
101	5.61	-	-	3.21	3.73	3.23	3.72	0.62	6.26	-	2.25	1.49	2.03	1.81	1.84	1.93	2.29	1.84	-	-	-	-	12.75
102	5.60	-	-	3.21	3.73	3.23	3.72	0.58	6.29	-	2.25	1.49	2.03	1.81	1.85	1.93	2.29	1.84	-	-	-	-	12.75
103	5.59	-	-	3.20	3.74	3.22	3.73	0.55	6.32	-	2.25	1.49	2.03	1.81	1.85	1.94	2.29	1.85	-	-	-	-	12.75
104	5.60	-	-	3.20	3.74	3.21	3.73	0.51	6.34	-	2.24	1.49	2.03	1.81	1.85	1.95	2.29	1.84	-	-	-	-	12.76
105	5.61	-	-	3.20	3.76	3.21	3.74	0.48	6.36	-	2.24	1.49	2.03	1.81	1.85	1.95	2.29	1.85	-	-	-	-	12.76
106	5.60	-	-	3.19	3.76	3.21	3.73	0.45	6.40	-	2.24	1.48	2.03	1.81	1.84	1.96	2.29	1.85	-	-	-	-	12.76
107	5.59	-	-	3.20	3.75	3.20	3.74	0.42	6.43	-	2.24	1.48	2.03	1.81	1.84	1.97	2.29	1.85	-	-	-	-	12.77
108	5.59	-	-	3.18	3.77	3.20	3.74	0.40	6.46	-	2.23	1.48	2.03	1.81	1.84	1.98	2.30	1.86	-	-	-	-	12.77
109	5.58	-	-	3.17	3.78	3.20	3.74	0.39	6.46	-	2.23	1.48	2.03	1.81	1.84	1.99	2.30	1.86	-	-	-	-	12.78
110	5.58	-	-	3.17	3.78	3.19	3.75	0.38	6.46	-	2.22	1.48	2.02	1.81	1.84	2.00	2.30	1.86	-	-	-	-	12.78
111	5.59	-	-	3.15	3.80	3.20	3.74	0.38	6.46	-	2.21	1.48	2.02	1.81	1.84	2.00	2.30	1.85	-	-	-	-	12.78
112	5.59	-	-	3.14	3.81	3.18	3.76	0.37	6.47	-	2.20	1.48	2.02	1.81	1.84	2.01	2.31	1.85	-	-	-	-	12.78
113	5.59	-	-	3.13	3.82	3.18	3.77	0.37	6.47	-	2.00	1.47	2.02	1.81	1.84	2.02	2.30	1.86	-	-	0.21	-	12.78
114	5.58	-	-	3.12	3.83	3.17	3.78	0.37	6.47	-	1.99	1.47	2.01	1.81	1.84	2.03	2.30	1.87	-	-	0.21	-	12.79
115	5.58	-	-	3.11	3.84	3.16	3.78	0.37	6.46	-	1.98	1.47	2.01	1.81	1.84	2.04	2.30	1.86	-	-	0.22	-	12.79
116	5.58	-	-	3.10	3.85	3.15	3.79	0.37	6.47	-	1.97	1.47	2.01	1.81	1.85	2.05	2.30	1.86	-	-	0.23	-	12.79
117	5.58	-	-	3.10	3.84	3.15	3.80	0.37	6.46	-	1.95	1.47	2.01	1.81	1.84	2.06	2.30	1.86	-	-	0.24	-	12.80
118	5.58	-	-	3.09	3.84	3.14	3.80	0.38	6.45	-	1.94	1.47	2.00	1.81	1.84	2.07	2.30	1.86	-	-	0.25	-	12.80
119	5.57	-	-	3.09	3.84	3.14	3.81	0.39	6.43	-	1.93	1.47	2.00	1.81	1.84	2.07	2.31	1.87	-	-	0.26	-	12.81
120	5.57	-	-	3.09	3.84	3.13	3.82	0.41	6.42	-	1.92	1.47	2.00	1.81	1.84	2.08	2.31	1.86	-	-	0.27	-	12.81
121	5.57	-	-	6.93	-	3.12	3.83	0.41	6.41	-	1.91	1.47	2.00	1.81	1.84	2.09	2.31	1.87	-	-	0.27	-	12.81
122	5.56	-	-	6.94	-	3.10	3.84	0.43	6.39	-	1.89	1.47	1.99	1.81	1.84	2.10	2.31	1.87	-	-	0.28	-	12.81
123	5.56	-	-	6.94	-	3.10	3.85	0.45	6.37	-	1.88	1.47	1.99	1.81	1.84	2.11	2.32	1.87	-	-	0.29	-	12.82
124	5.57	-	-	6.93	-	3.08	3.86	0.48	6.37	-	1.88	1.46	1.99	1.81	1.84	2.12	2.32	1.87	-	-	0.24	-	12.82
125	5.56	-	-	6.93	-	3.08	3.86	0.50	6.38	-	1.87	1.46	1.99	1.81	1.84	2.13	2.32	1.87	-	-	0.23	-	12.82
126	5.56	-	-	6.93	-	3.06	3.88	0.53	6.38	-	1.86	1.46	1.98	1.81	1.84	2.15	2.32	1.87	-	-	0.20	-	12.82
127	5.56	-	-	6.92	-	3.05	3.89	0.56	6.36	-	1.85	1.46	1.98	1.81	1.84	2.15	2.33	1.87	-	-	0.19	-	12.82
128	5.56	-	-	6.92	-	3.04	3.90	0.64	6.36	-	1.83	1.46	1.98	1.81	1.84	2.16	2.33	1.87	-	-	0.13	-	12.83
129	5.56	-	-	6.91	-	3.04	3.89	6.28	-	-	1.81	1.46	1.97	1.81	1.84	2.17	2.33	1.87	-	0.87	-	-	12.83
130	5.55	-	-	6.91	-	3.03	3.89	6.25	-	-	1.80	1.46	1.96	1.81	1.84	2.18	2.33	1.88	-	0.89	-	-	12.83
131	5.56	-	-	6.91	-	3.06	3.87	6.23	-	-	1.79	1.46	1.96	1.81	1.84	2.19	2.33	1.87	-	0.92	-	-	12.84
132	5.56	-	-	6.91	-	6.92	-	6.22	-	-	1.78	1.46	1.95	1.81	1.84	2.19	2.34	1.87	-	0.95	-	-	12.84
133	5.56	-	-	6.92	-	6.93	-	6.19	-	-	1.77	1.46	1.95	1.81	1.84	2.20	2.34	1.88	-	0.97	-	-	12.84

134	5.56	-	-	6.91	-	6.93	-	6.17	-	-	1.76	1.46	1.94	1.81	1.84	2.21	2.34	1.87	-	1.00	-	-	12.84
135	5.56	-	-	6.91	-	6.92	-	6.14	-	-	1.74	1.46	1.94	1.81	1.84	2.21	2.34	1.87	-	1.03	-	-	12.85
136	5.55	-	-	6.91	-	6.92	-	6.14	-	-	1.73	1.46	1.94	1.81	1.84	2.22	2.35	1.87	-	1.06	-	-	12.85
137	5.56	-	-	6.92	-	6.92	-	6.10	-	-	1.72	1.46	1.93	1.81	1.84	2.23	2.35	1.87	-	1.09	-	-	12.85
138	5.56	-	-	6.92	-	6.92	-	6.08	-	-	1.71	1.46	1.93	1.81	1.84	2.24	2.35	1.88	-	1.12	-	-	12.85
139	5.55	-	-	6.91	-	6.92	-	6.06	-	-	1.70	1.46	1.93	1.82	1.84	2.24	2.35	1.87	-	1.15	-	-	12.85
140	5.55	-	-	6.91	-	6.92	-	6.03	-	-	1.68	1.46	1.92	1.81	1.84	2.25	2.36	1.88	-	1.18	-	-	12.85
141	5.54	-	-	6.90	-	6.91	-	6.01	-	-	1.67	1.46	1.92	1.82	1.84	2.25	2.36	1.88	-	1.21	-	-	12.85
142	5.54	-	-	6.91	-	6.92	-	6.00	-	-	1.66	1.46	1.91	1.82	1.84	2.26	2.37	1.88	-	1.24	-	-	12.85
143	5.55	-	-	6.91	-	6.92	-	5.98	-	-	1.65	1.46	1.91	1.82	1.84	2.26	2.37	1.88	-	1.28	-	-	12.85
144	5.55	-	-	6.91	-	6.92	-	5.96	-	-	1.64	1.46	1.91	1.82	1.83	2.27	2.37	1.87	-	1.30	-	-	12.85
145	5.55	-	-	6.91	-	6.91	-	5.94	-	-	1.63	1.46	1.90	1.82	1.83	2.27	2.37	1.87	-	1.32	-	-	12.85
146	5.54	-	-	6.91	-	6.91	-	5.92	-	-	1.62	1.46	1.90	1.82	1.84	2.27	2.37	1.87	-	1.35	-	-	12.85
147	5.54	-	-	6.91	-	6.92	-	5.90	-	-	1.62	1.47	1.90	1.82	1.84	2.28	2.37	1.87	-	1.36	-	-	12.85
148	5.55	-	-	6.89	-	6.92	-	5.89	-	-	1.61	1.47	1.90	1.82	1.84	2.28	2.38	1.87	-	1.38	-	-	12.85
149	5.55	-	-	6.90	-	6.92	-	5.88	-	-	1.61	1.47	1.90	1.82	1.84	2.28	2.37	1.87	-	1.39	-	-	12.85
150	5.54	-	-	6.90	-	3.85	3.07	5.89	-	-	1.61	1.47	1.90	1.82	1.83	2.28	2.38	1.87	-	1.39	-	-	12.85
151	5.56	-	-	6.90	-	3.84	3.08	5.88	-	-	1.61	1.47	1.90	1.82	1.83	2.28	2.37	1.86	-	1.39	-	-	12.84
152	5.56	-	-	6.90	-	3.83	3.09	5.88	-	-	1.61	1.47	1.90	1.82	1.83	2.28	2.37	1.86	-	1.40	-	-	12.85
153	5.57	-	-	6.90	-	3.79	3.13	5.87	-	-	1.61	1.47	1.90	1.82	1.83	2.28	2.37	1.86	-	1.40	-	-	12.84
154	5.57	-	-	6.91	-	3.77	3.15	5.86	-	-	1.61	1.47	1.90	1.82	1.83	2.28	2.37	1.86	-	1.41	-	-	12.84
155	5.57	-	-	3.80	3.11	3.75	3.17	5.86	-	-	1.61	1.47	1.90	1.81	1.83	2.28	2.37	1.86	-	1.42	-	-	12.84
156	5.56	-	-	3.80	3.11	3.71	3.21	5.86	-	-	1.61	1.47	1.90	1.81	1.83	2.28	2.37	1.86	-	1.42	-	-	12.84
157	5.57	-	-	3.76	3.15	3.69	3.24	5.84	-	-	1.61	1.47	1.90	1.81	1.83	2.28	2.37	1.85	-	1.42	-	-	12.83
158	5.56	-	-	3.73	3.19	3.68	3.26	5.85	-	-	1.61	1.47	1.91	1.81	1.83	2.28	2.37	1.86	-	1.42	-	-	12.83
159	5.57	-	-	3.68	3.22	3.64	3.28	5.85	-	-	1.61	1.47	1.90	1.81	1.83	2.28	2.37	1.86	-	1.42	-	-	12.83
160	5.57	-	-	3.66	3.25	3.61	3.31	5.85	-	-	1.61	1.47	1.90	1.81	1.83	2.28	2.37	1.86	-	1.42	-	-	12.83
161	5.57	-	-	3.63	3.27	3.60	3.33	5.86	-	-	1.61	1.47	1.90	1.80	1.83	2.28	2.36	1.86	-	1.42	-	-	12.83
162	5.56	-	-	3.59	3.31	3.57	3.36	5.85	-	-	1.61	1.48	1.90	1.81	1.83	2.28	2.37	1.86	-	1.42	-	-	12.83
163	5.56	-	-	3.56	3.34	3.54	3.39	5.84	-	-	1.62	1.47	1.90	1.81	1.83	2.27	2.36	1.85	-	1.42	-	-	12.83
164	5.57	-	-	3.54	3.35	3.52	3.41	5.83	-	-	1.62	1.47	1.90	1.81	1.83	2.27	2.36	1.85	-	1.43	-	-	12.83
165	5.58	-	-	3.51	3.38	3.49	3.43	5.84	-	-	1.61	1.47	1.90	1.82	1.83	2.28	2.36	1.85	-	1.43	-	-	12.83
166	5.58	-	-	3.48	3.42	3.47	3.46	5.83	-	-	1.61	1.47	1.90	1.82	1.83	2.28	2.36	1.85	-	1.43	-	-	12.83
167	5.58	-	-	3.46	3.45	3.45	3.49	5.82	-	-	1.62	1.47	1.90	1.82	1.83	2.27	2.36	1.86	-	1.43	-	-	12.83
168	5.58	-	-	3.44	3.47	3.43	3.50	5.82	-	-	1.62	1.48	1.90	1.82	1.83	2.27	2.36	1.86	-	1.43	-	-	12.83

169	5.58	-	-	3.43	3.49	3.41	3.51	5.82	-	-	1.62	1.47	1.90	1.82	1.83	2.28	2.36	1.85	-	1.43	-	-	12.83
170	5.59	-	-	3.40	3.51	3.40	3.54	5.81	-	-	1.62	1.47	1.90	1.82	1.83	2.27	2.36	1.85	-	1.43	-	-	12.83
171	5.59	-	-	3.38	3.54	3.37	3.56	5.82	-	-	1.62	1.47	1.89	1.82	1.83	2.27	2.36	1.85	-	1.43	-	-	12.83
172	5.59	-	-	3.36	3.56	3.35	3.58	5.82	-	-	1.62	1.48	1.89	1.82	1.83	2.27	2.36	1.85	-	1.44	-	-	12.83
173	5.58	-	-	3.34	3.57	3.35	3.59	5.82	-	-	1.62	1.48	1.89	1.82	1.83	2.28	2.36	1.85	-	1.44	-	-	12.83
174	5.58	-	-	3.32	3.58	3.34	3.59	5.82	-	-	1.62	1.48	1.90	1.82	1.83	2.27	2.36	1.85	-	1.44	-	-	12.83
175	5.59	-	-	3.32	3.59	3.32	3.61	5.81	-	-	1.62	1.48	1.90	1.82	1.83	2.27	2.36	1.85	-	1.44	-	-	12.83
176	5.59	-	-	3.30	3.61	3.32	3.61	5.81	-	-	1.62	1.48	1.91	1.82	1.83	2.27	2.36	1.85	-	1.44	-	-	12.83
177	5.58	-	-	3.30	3.61	3.31	3.62	5.82	-	-	1.62	1.48	1.91	1.81	1.83	2.27	2.35	1.85	-	1.44	-	-	12.83
178	5.58	-	-	3.30	3.62	3.29	3.62	5.82	-	-	1.62	1.48	1.91	1.81	1.83	2.28	2.35	1.85	-	1.44	-	-	12.83
179	5.58	-	-	3.29	3.63	3.29	3.62	5.82	-	-	1.62	1.48	1.91	1.81	1.83	2.28	2.35	1.85	-	1.44	-	-	12.83
180	5.58	-	-	3.29	3.62	3.28	3.63	5.81	-	-	1.62	1.48	1.91	1.81	1.83	2.28	2.35	1.85	-	1.44	-	-	12.83
181	5.58	-	-	3.28	3.63	3.28	3.62	5.81	-	-	1.63	1.48	1.90	1.81	1.83	2.28	2.35	1.84	-	1.45	-	-	12.83
182	5.58	-	-	3.27	3.64	3.29	3.62	5.82	-	-	1.62	1.48	1.91	1.81	1.83	2.28	2.35	1.84	-	1.45	-	-	12.83
183	5.59	-	-	3.28	3.64	3.29	3.63	5.81	-	-	1.62	1.48	1.90	1.81	1.83	2.28	2.35	1.85	-	1.45	-	-	12.83
184	5.59	-	-	3.28	3.64	3.28	3.64	5.82	-	-	1.62	1.48	1.91	1.81	1.83	2.28	2.35	1.85	-	1.44	-	-	12.83
185	5.59	-	-	3.27	3.64	3.29	3.63	5.82	-	-	1.62	1.48	1.90	1.82	1.83	2.28	2.35	1.85	-	1.44	-	-	12.83
186	5.60	-	-	3.26	3.64	3.29	3.64	5.83	-	-	1.62	1.47	1.91	1.82	1.83	2.29	2.35	1.84	-	1.44	-	-	12.83
187	5.59	-	-	3.28	3.63	3.30	3.64	5.83	-	-	1.62	1.47	1.90	1.82	1.83	2.28	2.36	1.84	-	1.45	-	-	12.83
188	5.60	-	-	3.27	3.64	3.29	3.64	5.81	-	-	1.62	1.47	1.90	1.82	1.83	2.28	2.36	1.84	-	1.44	-	-	12.83
189	5.58	-	-	3.27	3.64	3.29	3.64	5.81	-	-	1.62	1.47	1.90	1.82	1.83	2.28	2.35	1.84	-	1.44	-	-	12.83
190	5.58	-	-	3.27	3.64	3.29	3.63	5.80	-	-	1.62	1.47	1.90	1.82	1.83	2.28	2.35	1.84	-	1.44	-	-	12.83
191	5.58	-	-	3.26	3.63	3.29	3.64	5.81	-	-	1.62	1.47	1.90	1.82	1.83	2.28	2.36	1.85	-	1.44	-	-	12.83
192	5.58	-	-	3.25	3.63	3.63	3.29	5.82	-	-	1.62	1.48	1.90	1.82	1.83	2.28	2.36	1.85	-	1.44	-	-	12.83
193	5.58	-	-	3.25	3.63	3.63	3.29	5.82	-	-	1.62	1.48	1.90	1.82	1.83	2.28	2.36	1.85	-	1.44	-	-	12.83
194	5.58	-	-	3.25	3.63	3.63	3.29	5.82	-	-	1.62	1.48	1.90	1.83	1.83	2.28	2.35	1.85	-	1.44	-	-	12.83
195	5.59	-	-	3.27	3.64	3.63	3.29	5.82	-	-	1.62	1.48	1.90	1.83	1.83	2.28	2.35	1.85	-	1.44	-	-	12.83
196	5.59	-	-	3.27	3.64	3.63	3.29	5.83	-	-	1.62	1.48	1.90	1.83	1.83	2.28	2.35	1.85	-	1.44	-	-	12.83
197	5.59	-	-	3.26	3.65	3.63	3.29	5.83	-	-	1.62	1.48	1.90	1.82	1.83	2.28	2.35	1.85	-	1.44	-	-	12.83
198	5.59	-	-	3.26	3.65	3.63	3.29	5.82	-	-	1.62	1.48	1.90	1.82	1.83	2.28	2.35	1.85	-	1.44	-	-	12.83
199	5.59	-	-	3.26	3.65	3.63	3.29	5.83	-	-	1.62	1.48	1.90	1.82	1.83	2.35	2.28	1.85	-	1.44	-	-	12.83
200	5.58	-	-	3.26	3.65	3.63	3.30	5.83	-	-	1.62	1.48	1.90	1.82	1.82	2.28	2.35	1.85	-	1.44	-	-	12.83
201	5.59	-	-	3.27	3.65	3.63	3.30	5.84	-	-	1.62	1.48	1.90	1.82	1.83	2.28	2.35	1.85	-	1.44	-	-	12.83
202	5.58	-	-	3.27	3.66	3.62	3.30	5.84	-	-	1.62	1.48	1.90	1.83	1.83	2.28	2.35	1.85	-	1.43	-	-	12.83
203	5.59	-	-	3.27	3.65	3.62	3.30	5.84	-	-	1.62	1.48	1.90	1.83	1.83	2.28	2.35	1.84	-	1.43	-	-	12.83

204	5.59	-	-	3.26	3.66	3.63	3.30	5.82	-	-	1.62	1.48	1.90	1.83	1.83	2.28	2.35	1.85	-	1.44	-	-	12.83
205	5.59	-	-	3.26	3.65	3.63	3.30	5.82	-	-	1.62	1.47	1.90	1.83	1.83	2.28	2.36	1.85	-	1.44	-	-	12.83
206	5.58	-	-	3.64	3.27	3.63	3.29	5.83	-	-	1.62	1.47	1.90	1.83	1.83	2.29	2.36	1.85	-	1.44	-	-	12.83
207	5.58	-	-	3.65	3.26	3.62	3.29	5.83	-	-	1.62	1.47	1.90	1.83	1.83	2.29	2.36	1.85	-	1.44	-	-	12.83
208	5.58	-	-	3.65	3.26	3.63	3.30	5.81	-	-	1.62	1.47	1.90	1.82	1.83	2.29	2.36	1.85	-	1.44	-	-	12.83
209	5.58	-	-	3.65	3.26	3.63	3.30	5.81	-	-	1.62	1.47	1.90	1.82	1.83	2.29	2.35	1.85	-	1.44	-	-	12.83
210	5.58	-	-	3.24	3.65	3.62	3.30	5.82	-	-	1.62	1.47	1.90	1.82	1.84	2.29	2.35	1.85	-	1.44	-	-	12.83
211	5.58	-	-	3.24	3.64	3.63	3.30	5.82	-	-	1.62	1.47	1.90	1.82	1.83	2.29	2.35	1.85	-	1.44	-	-	12.83
212	5.59	-	-	3.24	3.64	3.62	3.30	5.82	-	-	1.62	1.47	1.90	1.82	1.83	2.29	2.35	1.84	-	1.45	-	-	12.83
213	5.60	-	-	3.24	3.64	3.61	3.31	5.82	-	-	1.61	1.47	1.90	1.82	1.83	2.29	2.35	1.84	-	1.44	-	-	12.83
214	5.59	-	-	3.24	3.64	3.61	3.31	5.82	-	-	1.62	1.47	1.90	1.82	1.83	2.30	2.35	1.84	-	1.44	-	-	12.83
215	5.59	-	-	3.25	3.66	3.62	3.30	5.81	-	-	1.62	1.47	1.89	1.82	1.83	2.30	2.35	1.84	-	1.44	-	-	12.83
216	5.58	-	-	3.26	3.66	3.62	3.30	5.81	-	-	1.62	1.47	1.89	1.82	1.83	2.30	2.35	1.84	-	1.44	-	-	12.83
217	5.58	-	-	3.25	3.66	3.62	3.31	5.79	-	-	1.62	1.47	1.89	1.82	1.83	2.30	2.35	1.84	-	1.45	-	-	12.83
218	5.59	-	-	3.25	3.66	3.61	3.32	5.79	-	-	1.62	1.47	1.89	1.81	1.83	2.31	2.35	1.84	-	1.45	-	-	12.83
219	5.59	-	-	3.25	3.66	3.62	3.32	5.77	-	-	1.62	1.47	1.89	1.82	1.83	2.30	2.35	1.85	-	1.45	-	-	12.83
220	5.58	-	-	3.26	3.66	3.62	3.31	5.78	-	-	1.62	1.47	1.89	1.82	1.83	2.31	2.35	1.85	-	1.45	-	-	12.83
221	5.59	-	-	3.26	3.66	3.62	3.32	5.78	-	-	1.62	1.47	1.89	1.82	1.83	2.31	2.35	1.85	-	1.45	-	-	12.83
222	5.58	-	-	3.27	3.66	3.62	3.31	5.80	-	-	1.62	1.46	1.89	1.82	1.83	2.31	2.35	1.85	-	1.44	-	-	12.83
223	5.59	-	-	3.28	3.65	3.61	3.31	5.80	-	-	1.62	1.46	1.89	1.82	1.83	2.31	2.35	1.85	-	1.44	-	-	12.83
224	5.59	-	-	3.26	3.66	3.61	3.31	5.80	-	-	1.62	1.46	1.89	1.82	1.83	2.31	2.35	1.85	-	1.44	-	-	12.83
225	5.60	-	-	3.26	3.65	3.61	3.31	5.81	-	-	1.62	1.47	1.89	1.82	1.83	2.31	2.35	1.85	-	1.45	-	-	12.83
226	5.59	-	-	3.25	3.66	3.61	3.31	5.80	-	-	1.61	1.47	1.89	1.82	1.83	2.31	2.35	1.85	-	1.45	-	-	12.83
227	5.60	-	-	3.25	3.66	3.61	3.31	5.80	-	-	1.62	1.47	1.89	1.82	1.83	2.31	2.35	1.85	-	1.45	-	-	12.83
228	5.59	-	-	3.24	3.67	3.61	3.31	5.80	-	-	1.61	1.47	1.89	1.82	1.83	2.32	2.35	1.85	-	1.45	-	-	12.83
229	5.59	-	-	3.24	3.67	3.62	3.31	5.79	-	-	1.61	1.47	1.89	1.82	1.83	2.32	2.35	1.85	-	1.45	-	-	12.83
230	5.59	-	-	3.24	3.67	3.62	3.32	5.80	-	-	1.61	1.46	1.89	1.82	1.83	2.32	2.35	1.86	-	1.45	-	-	12.83

*ELF analysis for oxidation of pyrrolidine **R4***

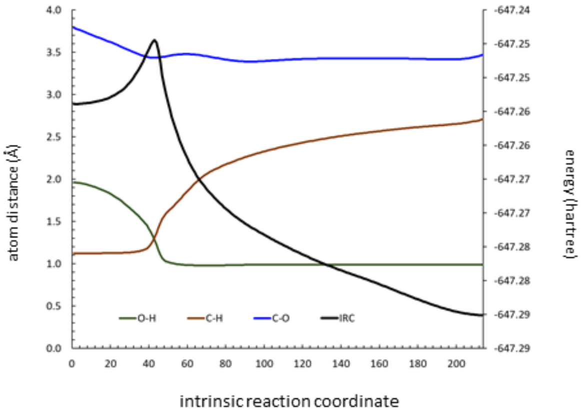

Figure S5. IRC analysis of the formation of **IN4a**.

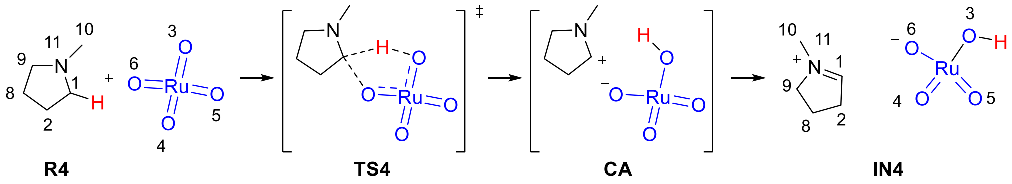

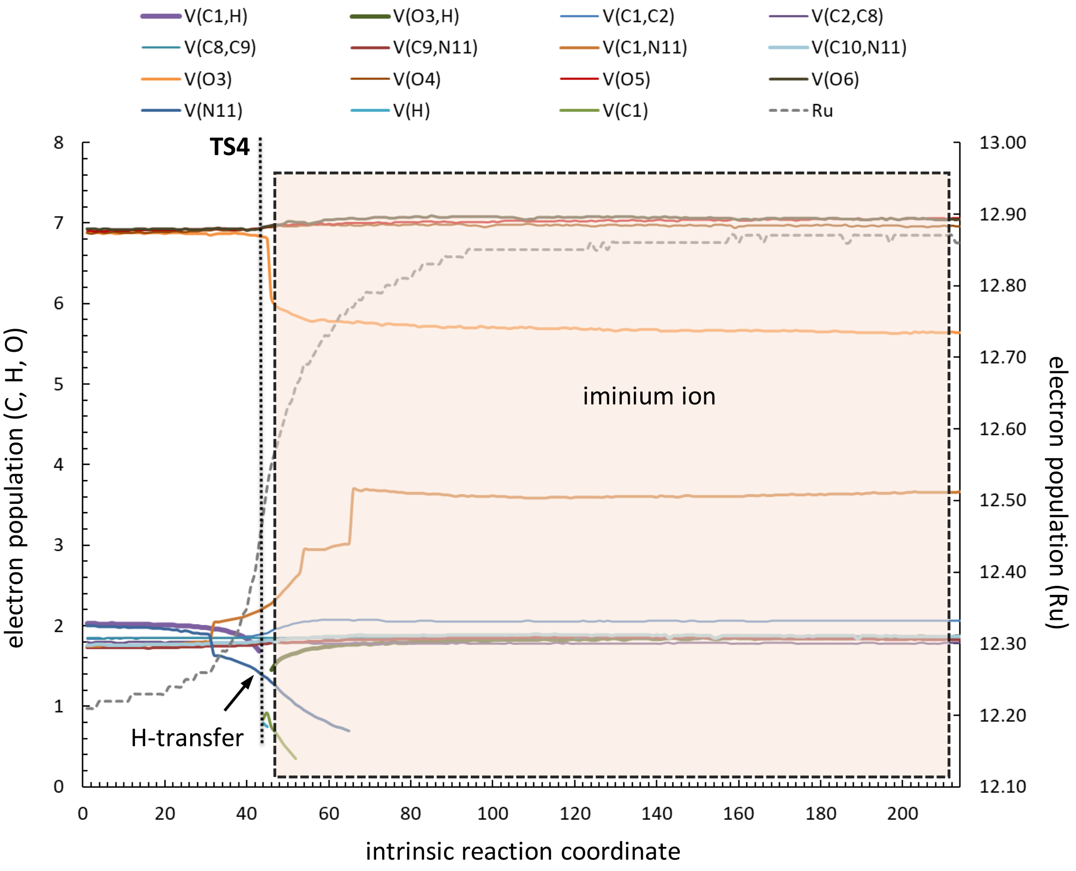

Figure S6. ELF analysis of the formation of **4a**..

Table S14. ELF basin populations for the oxidation of **R4** to give **IN4a**

	V1(O3)	V2(O3)	V3(O3)	V1(O4)	V2(O4)	V1(O5)	V2(O5)	V1(O6)	V2(O6)	V1(C1,N11)	V2(C1,N11)	V(C9,N11)	V(C1,C2)	V(C2,C8)	V(C8,C9)	V(C10,N11)	V1(N11)	V2(N11)	V(O3,H)	V(C1,H)	V(C1)	V(H)	Ru
1	6.88	-	-	6.88	-	6.90	-	5.39	1.54	1.75	-	1.73	1.84	1.80	1.85	1.76	1.76	0.24	-	2.03	-	-	12.21
2	6.88	-	-	6.87	-	6.90	-	5.40	1.53	1.75	-	1.73	1.84	1.79	1.85	1.76	1.76	0.24	-	2.03	-	-	12.21
3	6.88	-	-	6.88	-	6.89	-	5.41	1.52	1.75	-	1.73	1.84	1.80	1.85	1.77	1.76	0.24	-	2.03	-	-	12.21
4	6.88	-	-	6.89	-	6.90	-	5.41	1.51	1.75	-	1.73	1.84	1.80	1.85	1.77	1.76	0.23	-	2.02	-	-	12.22
5	6.87	-	-	6.88	-	6.89	-	5.39	1.54	1.76	-	1.73	1.84	1.80	1.84	1.77	1.76	0.23	-	2.02	-	-	12.22
6	6.88	-	-	6.88	-	6.90	-	5.39	1.54	1.76	-	1.73	1.85	1.80	1.85	1.76	1.76	0.23	-	2.02	-	-	12.22
7	6.88	-	-	6.89	-	6.91	-	5.38	1.54	1.76	-	1.73	1.85	1.80	1.84	1.76	1.76	0.23	-	2.03	-	-	12.22
8	6.87	-	-	6.89	-	6.90	-	5.38	1.54	1.76	-	1.73	1.85	1.79	1.85	1.76	1.76	0.23	-	2.02	-	-	12.22
9	6.87	-	-	6.88	-	6.90	-	5.38	1.54	1.76	-	1.73	1.84	1.79	1.85	1.76	1.75	0.23	-	2.02	-	-	12.22
10	6.88	-	-	6.88	-	6.90	-	5.40	1.52	1.76	-	1.73	1.85	1.79	1.85	1.76	1.76	0.23	-	2.02	-	-	12.22
11	6.88	-	-	6.88	-	6.90	-	5.41	1.51	1.76	-	1.73	1.85	1.80	1.85	1.76	1.75	0.23	-	2.02	-	-	12.22
12	6.88	-	-	6.88	-	6.91	-	5.42	1.50	1.76	-	1.73	1.85	1.80	1.85	1.76	1.75	0.23	-	2.02	-	-	12.23
13	6.88	-	-	6.89	-	6.91	-	5.43	1.50	1.76	-	1.73	1.85	1.80	1.84	1.76	1.75	0.23	-	2.02	-	-	12.23
14	6.88	-	-	6.89	-	6.91	-	5.43	1.50	1.76	-	1.73	1.85	1.80	1.85	1.76	1.74	0.24	-	2.02	-	-	12.23
15	6.87	-	-	6.88	-	6.91	-	5.43	1.50	1.76	-	1.73	1.85	1.80	1.85	1.77	1.74	0.24	-	2.02	-	-	12.23
16	6.88	-	-	6.88	-	6.91	-	5.44	1.48	1.77	-	1.72	1.85	1.80	1.85	1.77	1.74	0.24	-	2.02	-	-	12.23
17	6.88	-	-	6.89	-	4.82	2.09	5.45	1.48	1.77	-	1.73	1.85	1.80	1.85	1.77	1.73	0.24	-	2.01	-	-	12.23
18	6.88	-	-	6.89	-	4.62	2.29	5.41	1.52	1.77	-	1.73	1.85	1.80	1.85	1.77	1.73	0.24	-	2.01	-	-	12.23
19	6.88	-	-	6.89	-	2.42	4.49	5.37	1.56	1.77	-	1.73	1.85	1.80	1.85	1.77	1.73	0.24	-	2.01	-	-	12.23
20	6.87	-	-	4.95	1.94	2.50	4.41	5.22	1.71	1.77	-	1.73	1.85	1.79	1.85	1.77	1.72	0.24	-	2.01	-	-	12.23
21	6.88	-	-	4.74	2.15	2.67	4.24	5.25	1.68	1.77	-	1.73	1.85	1.80	1.85	1.77	1.72	0.24	-	2.01	-	-	12.24
22	6.88	-	-	4.56	2.34	2.75	4.16	5.06	1.87	1.78	-	1.73	1.85	1.80	1.85	1.77	0.24	1.71	-	2.01	-	-	12.24
23	6.88	-	-	4.48	2.42	2.81	4.10	4.73	2.19	1.78	-	1.73	1.85	1.80	1.85	1.77	1.71	0.24	-	2.00	-	-	12.24
24	6.88	-	-	4.35	2.54	2.86	4.05	4.54	2.38	1.78	-	1.74	1.85	1.80	1.85	1.77	1.70	0.23	-	2.00	-	-	12.24
25	6.87	-	-	4.30	2.59	2.92	4.01	4.41	2.52	1.79	-	1.74	1.85	1.80	1.85	1.77	1.70	0.24	-	2.00	-	-	12.25
26	6.87	-	-	4.25	2.65	2.99	3.93	4.31	2.61	1.79	-	1.74	1.85	1.80	1.85	1.77	1.69	0.24	-	2.00	-	-	12.25
27	6.87	-	-	4.22	2.68	3.01	3.91	4.20	2.72	1.80	-	1.74	1.85	1.80	1.85	1.77	0.23	1.68	-	1.99	-	-	12.25
28	6.87	-	-	4.15	2.75	3.03	3.89	4.11	2.81	1.80	-	1.74	1.85	1.80	1.85	1.78	0.24	1.67	-	1.99	-	-	12.26

29	6.87	-	-	4.12	2.79	3.07	3.85	4.03	2.89	1.80	-	1.74	1.85	1.80	1.85	1.77	1.66	0.24	-	1.98	-	-	12.26
30	6.87	-	-	4.12	2.79	3.08	3.85	3.97	2.96	1.81	-	1.75	1.85	1.80	1.85	1.78	1.66	0.24	-	1.98	-	-	12.26
31	6.85	-	-	4.12	2.79	3.11	3.82	3.91	3.02	1.81	-	1.75	1.85	1.80	1.85	1.78	1.65	0.23	-	1.97	-	-	12.26
32	6.86	-	-	4.12	2.80	3.79	3.14	3.86	3.07	2.04	-	1.75	1.85	1.79	1.85	1.79	1.64	-	-	1.96	-	-	12.27
33	6.87	-	-	4.13	2.79	3.12	3.80	3.82	3.12	2.05	-	1.75	1.85	1.79	1.85	1.79	1.63	-	-	1.96	-	-	12.28
34	6.87	-	-	4.12	2.79	3.14	3.79	3.77	3.16	2.06	-	1.75	1.85	1.79	1.85	1.79	1.62	-	-	1.95	-	-	12.29
35	6.87	-	-	4.12	2.79	3.14	3.79	3.74	3.19	2.07	-	1.75	1.85	1.79	1.85	1.79	1.60	-	-	1.94	-	-	12.29
36	6.87	-	-	4.09	2.82	3.13	3.78	3.72	3.21	2.08	-	1.75	1.85	1.79	1.85	1.79	1.59	-	-	1.92	-	-	12.3
37	6.88	-	-	4.05	2.86	3.14	3.77	3.69	3.24	2.09	-	1.75	1.85	1.79	1.85	1.79	1.57	-	-	1.91	-	-	12.31
38	6.87	-	-	4.04	2.87	3.14	3.78	3.66	3.27	2.10	-	1.76	1.86	1.79	1.85	1.79	1.55	-	-	1.89	-	-	12.32
39	6.87	-	-	4.04	2.88	3.14	3.78	3.64	3.29	2.11	-	1.76	1.86	1.79	1.85	1.79	1.53	-	-	1.87	-	-	12.34
40	6.86	-	-	4.02	2.90	3.13	3.78	3.61	3.30	2.13	-	1.76	1.87	1.79	1.85	1.79	1.51	-	-	1.84	-	-	12.35
41	3.14	3.71	-	4.01	2.92	3.13	3.79	3.59	3.33	2.15	-	1.76	1.87	1.79	1.85	1.80	1.49	-	-	1.80	-	-	12.38
42	3.21	3.64	-	3.97	2.96	3.14	3.79	3.57	3.35	2.17	-	1.77	1.88	1.79	1.85	1.80	1.46	-	-	1.75	-	-	12.4
43	2.91	3.57	0.36	3.93	3.00	3.15	3.78	3.56	3.37	2.19	-	1.78	1.89	1.79	1.85	1.81	1.42	-	-	1.69	-	-	12.44
44	6.33	0.50	-	3.89	3.05	3.17	3.77	3.56	3.39	2.22	-	1.78	1.90	1.79	1.85	1.81	1.38	-	-	-	0.84	0.79	12.48
45	4.64	2.16	-	3.85	3.10	3.21	3.74	3.56	3.40	2.25	-	1.78	1.91	1.79	1.85	1.82	1.35	-	-	-	0.92	0.75	12.52
46	6.07	-	-	3.80	3.15	3.25	3.71	3.57	3.40	2.28	-	1.79	1.94	1.79	1.85	1.82	1.30	-	1.45	-	0.75	-	12.55
47	5.98	-	-	3.77	3.19	3.28	3.69	3.57	3.41	2.33	-	1.79	1.96	1.79	1.85	1.79	1.26	-	1.53	-	0.68	-	12.57
48	5.94	-	-	3.74	3.23	3.30	3.67	3.58	3.40	2.38	-	1.80	1.97	1.79	1.85	1.83	1.20	-	1.58	-	0.61	-	12.59
49	5.92	-	-	3.72	3.24	3.32	3.66	3.58	3.41	2.44	-	1.79	1.99	1.79	1.85	1.83	1.15	-	1.61	-	0.53	-	12.61
50	5.90	-	-	3.70	3.26	3.33	3.64	3.60	3.42	2.49	-	1.79	2.00	1.79	1.85	1.84	1.11	-	1.63	-	0.47	-	12.63
51	5.87	-	-	3.70	3.26	3.34	3.64	3.60	3.42	2.55	-	1.80	2.02	1.79	1.85	1.85	1.07	-	1.65	-	0.41	-	12.64
52	5.85	-	-	3.68	3.28	3.35	3.63	3.59	3.42	2.61	-	1.80	2.03	1.79	1.85	1.85	1.02	-	1.66	-	0.35	-	12.66
53	5.83	-	-	3.66	3.31	3.37	3.62	3.59	3.42	2.67	-	1.80	2.04	1.79	1.85	1.85	0.99	-	1.68	-	-	-	12.67
54	5.81	-	-	3.65	3.32	3.37	3.61	3.60	3.41	2.95	-	1.80	2.05	1.79	1.85	1.85	0.95	-	1.70	-	-	-	12.69
55	5.79	-	-	3.63	3.34	3.38	3.60	3.59	3.40	2.95	-	1.81	2.05	1.79	1.85	1.85	0.92	-	1.71	-	-	-	12.69
56	2.76	3.02	-	3.62	3.36	3.39	3.60	3.61	3.39	2.95	-	1.81	2.06	1.78	1.85	1.86	0.89	-	1.72	-	-	-	12.7
57	2.87	2.92	-	3.62	3.36	3.40	3.58	3.62	3.39	2.95	-	1.81	2.06	1.78	1.85	1.86	0.86	-	1.72	-	-	-	12.71
58	2.96	2.84	-	3.61	3.37	3.41	3.59	3.63	3.39	2.95	-	1.81	2.07	1.78	1.85	1.86	0.84	-	1.73	-	-	-	12.72
59	3.03	2.77	-	3.61	3.37	3.41	3.57	3.65	3.39	2.95	-	1.82	2.07	1.78	1.85	1.87	0.82	-	1.74	-	-	-	12.73
60	3.07	2.71	-	3.60	3.38	3.42	3.57	3.66	3.38	2.97	-	1.82	2.07	1.78	1.85	1.87	0.79	-	1.74	-	-	-	12.73
61	3.12	2.66	-	3.58	3.39	3.41	3.57	3.66	3.38	2.99	-	1.82	2.07	1.78	1.85	1.87	0.76	-	1.75	-	-	-	12.74
62	3.14	2.64	-	3.57	3.41	3.43	3.56	3.67	3.38	3.00	-	1.82	2.07	1.78	1.86	1.87	0.74	-	1.76	-	-	-	12.75
63	3.14	2.64	-	3.56	3.41	3.43	3.56	3.67	3.38	3.01	-	1.82	2.07	1.78	1.85	1.87	0.73	-	1.76	-	-	-	12.76

64	3.15	2.62	-	3.55	3.42	3.43	3.57	3.67	3.38	3.02	-	1.83	2.07	1.78	1.86	1.87	0.71	-	1.77	-	-	-	12.76
65	3.18	2.59	-	3.55	3.43	3.44	3.56	3.68	3.38	3.03	-	1.83	2.07	1.78	1.85	1.88	0.69	-	1.77	-	-	-	12.77
66	3.19	2.57	-	3.53	3.44	3.44	3.56	3.68	3.38	3.70	-	1.83	2.06	1.79	1.85	1.88	-	-	1.77	-	-	-	12.77
67	3.22	2.56	-	3.53	3.44	3.44	3.56	3.67	3.38	3.69	-	1.83	2.07	1.79	1.85	1.89	-	-	1.77	-	-	-	12.78
68	3.21	2.56	-	3.52	3.45	3.44	3.56	3.68	3.37	3.68	-	1.83	2.07	1.78	1.85	1.89	-	-	1.78	-	-	-	12.78
69	3.20	2.56	-	3.52	3.46	3.44	3.56	3.68	3.37	1.75	1.95	1.83	2.07	1.78	1.85	1.88	-	-	1.78	-	-	-	12.79
70	3.20	2.56	-	3.52	3.46	3.45	3.55	3.68	3.38	1.77	1.92	1.83	2.07	1.78	1.85	1.88	-	-	1.77	-	-	-	12.79
71	3.20	2.56	-	3.51	3.46	3.45	3.55	3.69	3.38	1.79	1.90	1.83	2.07	1.78	1.85	1.88	-	-	1.78	-	-	-	12.79
72	3.19	2.56	-	3.51	3.47	3.46	3.55	3.69	3.38	1.80	1.88	1.83	2.07	1.77	1.85	1.88	-	-	1.78	-	-	-	12.79
73	3.18	2.55	-	3.52	3.47	3.45	3.55	3.69	3.38	1.81	1.87	1.83	2.06	1.78	1.85	1.88	-	-	1.79	-	-	-	12.79
74	3.19	2.56	-	3.51	3.47	3.45	3.55	3.70	3.37	1.81	1.87	1.83	2.05	1.79	1.85	1.88	-	-	1.78	-	-	-	12.8
75	3.18	2.57	-	3.52	3.46	3.45	3.54	3.69	3.37	1.80	1.87	1.83	2.05	1.79	1.85	1.88	-	-	1.79	-	-	-	12.8
76	3.18	2.56	-	3.53	3.45	3.45	3.56	3.70	3.36	1.80	1.87	1.84	2.05	1.79	1.85	1.88	-	-	1.80	-	-	-	12.8
77	3.18	2.56	-	3.53	3.44	3.45	3.56	3.70	3.37	1.79	1.87	1.84	2.05	1.79	1.85	1.88	-	-	1.79	-	-	-	12.81
78	3.17	2.57	-	3.55	3.43	3.44	3.57	3.71	3.36	1.79	1.87	1.84	2.05	1.79	1.85	1.88	-	-	1.79	-	-	-	12.81
79	3.17	2.57	-	3.56	3.42	3.44	3.57	3.71	3.36	1.78	1.87	1.84	2.05	1.79	1.86	1.88	-	-	1.79	-	-	-	12.81
80	3.16	2.57	-	3.58	3.40	3.43	3.58	3.71	3.36	1.78	1.87	1.84	2.05	1.78	1.86	1.88	-	-	1.80	-	-	-	12.81
81	3.16	2.57	-	3.59	3.40	3.43	3.58	3.72	3.36	1.78	1.87	1.84	2.05	1.78	1.85	1.88	-	-	1.81	-	-	-	12.82
82	3.16	2.55	-	3.59	3.39	3.42	3.59	3.72	3.36	1.78	1.87	1.84	2.06	1.78	1.85	1.88	-	-	1.81	-	-	-	12.82
83	3.15	2.57	-	3.61	3.37	3.42	3.60	3.72	3.35	1.78	1.87	1.84	2.06	1.78	1.86	1.89	-	-	1.81	-	-	-	12.83
84	3.15	2.57	-	3.61	3.36	3.42	3.60	3.73	3.34	1.78	1.86	1.84	2.06	1.78	1.86	1.88	-	-	1.80	-	-	-	12.83
85	3.15	2.57	-	3.63	3.35	3.40	3.62	3.74	3.35	1.78	1.86	1.84	2.06	1.78	1.86	1.88	-	-	1.80	-	-	-	12.83
86	3.15	2.57	-	3.64	3.35	3.40	3.61	3.74	3.34	1.78	1.86	1.84	2.05	1.78	1.85	1.88	-	-	1.81	-	-	-	12.83
87	3.15	2.56	-	3.64	3.34	3.40	3.61	3.74	3.33	1.78	1.86	1.84	2.05	1.78	1.85	1.88	-	-	1.81	-	-	-	12.83
88	3.15	2.55	-	3.66	3.32	3.40	3.62	3.74	3.33	1.78	1.85	1.85	2.05	1.78	1.85	1.88	-	-	1.82	-	-	-	12.84
89	3.16	2.55	-	3.67	3.31	3.39	3.62	3.74	3.33	1.78	1.85	1.85	2.05	1.78	1.86	1.89	-	-	1.82	-	-	-	12.84
90	3.15	2.56	-	3.68	3.30	3.39	3.62	3.75	3.33	1.77	1.85	1.85	2.05	1.79	1.86	1.89	-	-	1.81	-	-	-	12.84
91	3.14	2.57	-	3.69	3.30	3.38	3.62	3.76	3.32	1.77	1.84	1.85	2.05	1.78	1.86	1.89	-	-	1.81	-	-	-	12.84
92	3.16	2.56	-	3.70	3.29	3.38	3.63	3.76	3.32	1.78	1.84	1.85	2.05	1.79	1.86	1.89	-	-	1.82	-	-	-	12.84
93	3.16	2.54	-	3.71	3.27	3.38	3.63	3.76	3.32	1.78	1.84	1.85	2.05	1.79	1.86	1.89	-	-	1.82	-	-	-	12.84
94	3.16	2.54	-	3.71	3.26	3.38	3.63	3.76	3.32	1.77	1.84	1.85	2.05	1.78	1.86	1.89	-	-	1.82	-	-	-	12.85
95	3.17	2.53	-	3.73	3.24	3.38	3.63	3.31	3.77	1.78	1.84	1.85	2.05	1.78	1.86	1.89	-	-	1.83	-	-	-	12.85
96	3.17	2.53	-	3.73	3.24	3.38	3.63	3.31	3.77	1.78	1.83	1.85	2.05	1.78	1.86	1.89	-	-	1.83	-	-	-	12.85
97	3.18	2.54	-	3.73	3.23	3.38	3.63	3.31	3.77	1.78	1.83	1.85	2.05	1.78	1.86	1.89	-	-	1.82	-	-	-	12.85
98	3.18	2.53	-	3.73	3.22	3.39	3.62	3.31	3.77	1.78	1.83	1.85	2.05	1.78	1.86	1.89	-	-	1.82	-	-	-	12.85

99	3.17	2.53	-	3.74	3.22	3.39	3.62	3.31	3.77	1.78	1.83	1.85	2.05	1.78	1.86	1.89	-	-	1.82	-	-	-	12.85
100	3.18	2.53	-	3.74	3.23	3.39	3.63	3.31	3.77	1.79	1.83	1.85	2.05	1.78	1.86	1.89	-	-	1.83	-	-	-	12.85
101	3.19	2.51	-	3.75	3.23	3.39	3.62	3.31	3.77	1.79	1.82	1.85	2.05	1.78	1.86	1.89	-	-	1.83	-	-	-	12.85
102	3.19	2.51	-	3.76	3.22	3.40	3.62	3.30	3.77	1.79	1.82	1.85	2.05	1.78	1.86	1.89	-	-	1.83	-	-	-	12.85
103	3.20	2.50	-	3.77	3.21	3.40	3.63	3.30	3.77	1.79	1.82	1.85	2.05	1.78	1.85	1.89	-	-	1.83	-	-	-	12.85
104	3.20	2.49	-	3.76	3.22	3.40	3.63	3.30	3.77	1.78	1.82	1.85	2.05	1.79	1.86	1.89	-	-	1.83	-	-	-	12.85
105	3.21	2.49	-	3.77	3.21	3.40	3.63	3.31	3.76	1.78	1.82	1.86	2.05	1.78	1.85	1.89	-	-	1.83	-	-	-	12.85
106	3.21	2.49	-	3.76	3.22	3.41	3.62	3.30	3.76	1.78	1.82	1.85	2.05	1.78	1.85	1.89	-	-	1.83	-	-	-	12.85
107	3.22	2.49	-	3.76	3.22	3.42	3.61	3.30	3.75	1.78	1.82	1.86	2.05	1.78	1.86	1.89	-	-	1.83	-	-	-	12.85
108	3.22	2.48	-	3.77	3.22	3.42	3.60	3.28	3.76	1.77	1.82	1.86	2.06	1.78	1.86	1.89	-	-	1.83	-	-	-	12.85
109	3.23	2.48	-	3.78	3.21	3.42	3.60	3.29	3.76	1.77	1.82	1.86	2.06	1.78	1.86	1.89	-	-	1.83	-	-	-	12.85
110	3.22	2.48	-	3.76	3.22	3.42	3.60	3.30	3.75	1.77	1.82	1.86	2.05	1.78	1.86	1.90	-	-	1.83	-	-	-	12.85
111	3.22	2.47	-	3.76	3.21	3.41	3.61	3.31	3.76	1.77	1.82	1.86	2.05	1.78	1.85	1.90	-	-	1.83	-	-	-	12.85
112	3.23	2.46	-	3.76	3.21	3.43	3.59	3.31	3.76	1.77	1.82	1.86	2.05	1.78	1.85	1.89	-	-	1.84	-	-	-	12.85
113	3.24	2.45	-	3.76	3.22	3.43	3.59	3.31	3.76	1.77	1.82	1.86	2.05	1.78	1.85	1.89	-	-	1.84	-	-	-	12.85
114	3.22	2.47	-	3.76	3.21	3.44	3.58	3.31	3.76	1.77	1.83	1.86	2.05	1.78	1.86	1.89	-	-	1.83	-	-	-	12.85
115	3.21	2.48	-	3.76	3.22	3.44	3.58	3.31	3.76	1.77	1.83	1.86	2.05	1.79	1.86	1.90	-	-	1.83	-	-	-	12.85
116	3.21	2.48	-	3.76	3.22	3.45	3.58	3.32	3.76	1.77	1.83	1.86	2.05	1.79	1.86	1.89	-	-	1.83	-	-	-	12.85
117	3.22	2.48	-	3.76	3.21	3.44	3.59	3.32	3.75	1.77	1.83	1.86	2.05	1.78	1.86	1.89	-	-	1.82	-	-	-	12.85
118	3.21	2.49	-	3.77	3.21	3.43	3.59	3.32	3.75	1.77	1.83	1.86	2.05	1.79	1.86	1.89	-	-	1.83	-	-	-	12.85
119	3.22	2.47	-	3.77	3.20	3.43	3.59	3.32	3.75	1.76	1.83	1.86	2.05	1.78	1.86	1.89	-	-	1.84	-	-	-	12.85
120	3.21	2.49	-	3.77	3.21	3.46	3.57	3.32	3.75	1.77	1.83	1.87	2.05	1.78	1.85	1.89	-	-	1.84	-	-	-	12.85
121	3.20	2.49	-	3.77	3.21	3.46	3.56	3.32	3.75	1.77	1.83	1.87	2.05	1.78	1.85	1.89	-	-	1.83	-	-	-	12.85
122	5.68	-	-	3.76	3.21	3.47	3.55	3.33	3.74	1.77	1.83	1.86	2.06	1.78	1.85	1.89	-	-	1.84	-	-	-	12.85
123	5.68	-	-	3.76	3.21	3.46	3.56	3.33	3.75	1.77	1.83	1.86	2.05	1.78	1.85	1.89	-	-	1.84	-	-	-	12.85
124	5.69	-	-	3.76	3.20	3.45	3.58	3.33	3.75	1.77	1.83	1.87	2.05	1.78	1.86	1.88	-	-	1.83	-	-	-	12.86
125	5.68	-	-	3.77	3.19	3.45	3.57	3.33	3.75	1.77	1.83	1.87	2.05	1.78	1.86	1.89	-	-	1.83	-	-	-	12.85
126	5.69	-	-	3.77	3.20	3.47	3.56	3.34	3.74	1.77	1.83	1.87	2.06	1.78	1.86	1.89	-	-	1.84	-	-	-	12.85
127	5.67	-	-	3.78	3.19	3.48	3.55	3.33	3.74	1.77	1.83	1.86	2.05	1.78	1.86	1.89	-	-	1.85	-	-	-	12.86
128	5.68	-	-	3.79	3.19	3.48	3.56	3.34	3.74	1.78	1.83	1.86	2.06	1.78	1.86	1.89	-	-	1.84	-	-	-	12.85
129	5.67	-	-	3.78	3.20	3.47	3.56	3.33	3.74	1.78	1.82	1.87	2.05	1.78	1.86	1.88	-	-	1.85	-	-	-	12.86
130	5.66	-	-	3.78	3.20	3.44	3.58	3.33	3.75	1.78	1.83	1.86	2.05	1.78	1.86	1.89	-	-	1.85	-	-	-	12.86
131	5.67	-	-	3.78	3.19	3.45	3.58	3.34	3.74	1.78	1.83	1.86	2.05	1.78	1.86	1.89	-	-	1.84	-	-	-	12.86
132	5.68	-	-	3.78	3.19	3.46	3.57	3.34	3.74	1.78	1.83	1.86	2.05	1.78	1.86	1.89	-	-	1.83	-	-	-	12.86
133	5.68	-	-	3.78	3.19	3.48	3.55	3.34	3.74	1.78	1.83	1.86	2.05	1.78	1.86	1.89	-	-	1.84	-	-	-	12.86

134	5.67	-	-	3.78	3.19	3.48	3.56	3.35	3.73	1.78	1.83	1.86	2.05	1.78	1.86	1.89	-	-	1.85	-	-	-	12.86
135	5.67	-	-	3.79	3.19	3.46	3.57	3.35	3.72	1.78	1.83	1.86	2.05	1.78	1.86	1.89	-	-	1.85	-	-	-	12.86
136	5.68	-	-	3.79	3.19	3.44	3.59	3.35	3.72	1.78	1.83	1.86	2.05	1.78	1.86	1.89	-	-	1.85	-	-	-	12.86
137	5.68	-	-	3.79	3.18	3.45	3.57	3.35	3.72	1.78	1.83	1.86	2.05	1.78	1.85	1.89	-	-	1.85	-	-	-	12.86
138	5.68	-	-	3.79	3.19	3.46	3.57	3.35	3.71	1.78	1.83	1.86	2.06	1.78	1.85	1.89	-	-	1.85	-	-	-	12.86
139	5.68	-	-	3.80	3.18	3.47	3.56	3.35	3.72	1.78	1.83	1.86	2.06	1.78	1.85	1.88	-	-	1.84	-	-	-	12.86
140	5.68	-	-	3.78	3.20	3.47	3.57	3.35	3.72	1.78	1.82	1.85	2.06	1.78	1.85	1.89	-	-	1.84	-	-	-	12.86
141	5.66	-	-	3.78	3.19	3.46	3.58	3.35	3.72	1.79	1.83	1.85	2.06	1.78	1.85	1.88	-	-	1.84	-	-	-	12.86
142	5.66	-	-	3.80	3.17	3.45	3.58	3.36	3.72	1.78	1.83	1.85	2.06	1.79	1.85	1.89	-	-	1.85	-	-	-	12.86
143	5.66	-	-	3.79	3.17	3.45	3.58	3.35	3.72	1.78	1.83	1.85	2.06	1.79	1.85	1.85	-	-	1.85	-	-	-	12.86
144	5.66	-	-	3.80	3.17	3.46	3.57	3.35	3.72	1.79	1.82	1.85	2.06	1.78	1.86	1.88	-	-	1.86	-	-	-	12.86
145	5.66	-	-	3.80	3.17	3.45	3.57	3.35	3.72	1.78	1.82	1.85	2.06	1.79	1.86	1.88	-	-	1.85	-	-	-	12.86
146	5.67	-	-	3.80	3.17	3.46	3.58	3.35	3.71	1.78	1.83	1.85	2.05	1.79	1.85	1.88	-	-	1.84	-	-	-	12.86
147	5.67	-	-	3.80	3.17	3.45	3.59	3.36	3.71	1.79	1.83	1.86	2.05	1.79	1.85	1.89	-	-	1.84	-	-	-	12.86
148	5.67	-	-	3.81	3.17	3.44	3.60	3.35	3.71	1.79	1.83	1.86	2.06	1.79	1.85	1.89	-	-	1.84	-	-	-	12.86
149	5.66	-	-	3.81	3.16	3.45	3.59	3.35	3.71	1.78	1.83	1.86	2.06	1.79	1.85	1.89	-	-	1.85	-	-	-	12.86
150	5.66	-	-	3.81	3.16	3.45	3.59	3.34	3.72	1.78	1.83	1.86	2.05	1.79	1.85	1.88	-	-	1.85	-	-	-	12.86
151	5.66	-	-	3.81	3.16	3.44	3.60	3.34	3.72	1.78	1.83	1.86	2.05	1.79	1.85	1.88	-	-	1.86	-	-	-	12.86
152	5.67	-	-	3.81	3.16	3.44	3.60	3.34	3.72	1.78	1.83	1.85	2.05	1.79	1.85	1.88	-	-	1.86	-	-	-	12.86
153	5.67	-	-	3.81	3.15	3.43	3.61	3.35	3.72	1.78	1.83	1.85	2.05	1.79	1.85	1.88	-	-	1.85	-	-	-	12.86
154	5.67	-	-	3.82	3.16	3.42	3.61	3.35	3.71	1.78	1.83	1.85	2.05	1.79	1.85	1.85	-	-	1.84	-	-	-	12.86
155	5.67	-	-	3.82	3.16	3.43	3.61	3.35	3.71	1.78	1.83	1.86	2.06	1.79	1.85	1.86	-	-	1.84	-	-	-	12.86
156	5.67	-	-	3.82	3.16	3.42	3.61	3.35	3.71	1.78	1.83	1.85	2.06	1.79	1.86	1.88	-	-	1.85	-	-	-	12.86
157	5.66	-	-	3.82	3.15	3.42	3.61	3.35	3.71	1.78	1.83	1.85	2.06	1.79	1.86	1.88	-	-	1.85	-	-	-	12.86
158	5.66	-	-	3.82	3.15	3.42	3.62	3.35	3.71	1.78	1.83	1.85	2.06	1.78	1.86	1.88	-	-	1.85	-	-	-	12.87
159	5.66	-	-	3.79	3.15	3.42	3.63	3.34	3.71	1.78	1.83	1.85	2.06	1.78	1.86	1.88	-	-	1.86	-	-	-	12.87
160	5.67	-	-	3.81	3.15	3.41	3.62	3.35	3.69	1.78	1.83	1.85	2.06	1.78	1.86	1.88	-	-	1.85	-	-	-	12.86
161	5.67	-	-	3.81	3.15	3.41	3.62	3.35	3.70	1.78	1.83	1.85	2.06	1.78	1.86	1.88	-	-	1.85	-	-	-	12.87
162	5.67	-	-	3.82	3.15	3.41	3.62	3.35	3.70	1.79	1.83	1.85	2.05	1.78	1.86	1.88	-	-	1.85	-	-	-	12.87
163	5.66	-	-	3.82	3.15	3.41	3.62	3.34	3.70	1.79	1.83	1.85	2.05	1.78	1.86	1.88	-	-	1.85	-	-	-	12.87
164	5.67	-	-	3.82	3.15	3.41	3.63	3.34	3.71	1.79	1.83	1.84	2.05	1.78	1.86	1.88	-	-	1.84	-	-	-	12.87
165	5.67	-	-	3.82	3.15	3.41	3.64	3.33	3.71	1.79	1.83	1.84	2.05	1.78	1.86	1.88	-	-	1.85	-	-	-	12.87
166	5.66	-	-	3.82	3.15	3.41	3.64	3.34	3.71	1.79	1.84	1.84	2.05	1.79	1.86	1.88	-	-	1.85	-	-	-	12.86
167	5.66	-	-	3.83	3.14	3.41	3.63	3.35	3.71	1.79	1.83	1.84	2.05	1.79	1.86	1.88	-	-	1.85	-	-	-	12.87
168	5.66	-	-	3.83	3.14	3.42	3.63	3.36	3.70	1.79	1.83	1.84	2.06	1.79	1.86	1.88	-	-	1.85	-	-	-	12.87

169	5.66	-	-	3.82	3.15	3.42	3.63	3.36	3.70	1.79	1.84	1.85	2.06	1.79	1.86	1.88	-	-	1.85	-	-	-	12.87
170	5.66	-	-	3.82	3.15	3.40	3.64	3.36	3.69	1.79	1.84	1.85	2.06	1.79	1.86	1.88	-	-	1.85	-	-	-	12.87
171	5.67	-	-	3.82	3.15	3.40	3.64	3.36	3.70	1.79	1.84	1.84	2.06	1.79	1.86	1.87	-	-	1.85	-	-	-	12.87
172	5.66	-	-	3.84	3.14	3.39	3.65	3.36	3.70	1.79	1.84	1.84	2.06	1.79	1.86	1.88	-	-	1.85	-	-	-	12.87
173	5.66	-	-	3.84	3.13	3.39	3.65	3.36	3.70	1.79	1.85	1.84	2.06	1.79	1.86	1.88	-	-	1.85	-	-	-	12.87
174	5.67	-	-	3.83	3.13	3.39	3.64	3.37	3.69	1.79	1.84	1.84	2.06	1.78	1.86	1.87	-	-	1.85	-	-	-	12.87
175	5.66	-	-	3.84	3.13	3.40	3.64	3.37	3.69	1.79	1.84	1.84	2.06	1.78	1.85	1.87	-	-	1.85	-	-	-	12.87
176	5.66	-	-	3.84	3.13	3.40	3.64	3.37	3.69	1.79	1.84	1.84	2.06	1.78	1.86	1.87	-	-	1.85	-	-	-	12.87
177	5.66	-	-	3.86	3.11	3.39	3.65	3.37	3.69	1.80	1.84	1.84	2.06	1.78	1.86	1.88	-	-	1.85	-	-	-	12.87
178	5.66	-	-	3.86	3.11	3.40	3.65	3.36	3.70	1.79	1.84	1.84	2.06	1.78	1.86	1.87	-	-	1.85	-	-	-	12.87
179	5.65	-	-	3.84	3.14	3.39	3.66	3.36	3.70	1.79	1.84	1.84	2.06	1.78	1.86	1.88	-	-	1.85	-	-	-	12.87
180	5.65	-	-	3.83	3.14	3.40	3.65	3.36	3.69	1.80	1.84	1.84	2.06	1.78	1.86	1.88	-	-	1.85	-	-	-	12.87
181	5.65	-	-	3.84	3.14	3.39	3.65	3.36	3.69	1.79	1.84	1.84	2.06	1.78	1.86	1.88	-	-	1.85	-	-	-	12.87
182	5.65	-	-	3.86	3.12	3.39	3.67	3.37	3.69	1.80	1.84	1.84	2.06	1.78	1.86	1.88	-	-	1.86	-	-	-	12.87
183	5.66	-	-	3.85	3.12	3.40	3.65	3.37	3.69	1.80	1.84	1.84	2.06	1.78	1.86	1.88	-	-	1.85	-	-	-	12.87
184	5.66	-	-	3.84	3.13	3.40	3.65	3.36	3.70	1.80	1.84	1.84	2.06	1.78	1.86	1.88	-	-	1.85	-	-	-	12.87
185	5.65	-	-	3.84	3.13	3.40	3.65	3.35	3.70	1.80	1.84	1.84	2.06	1.78	1.86	1.88	-	-	1.85	-	-	-	12.87
186	5.65	-	-	3.85	3.13	3.40	3.65	3.35	3.70	1.80	1.84	1.84	2.06	1.78	1.86	1.88	-	-	1.85	-	-	-	12.86
187	5.65	-	-	3.85	3.11	3.39	3.66	3.36	3.69	1.80	1.84	1.84	2.06	1.78	1.86	1.88	-	-	1.85	-	-	-	12.86
188	5.65	-	-	3.85	3.11	3.38	3.66	3.36	3.69	1.80	1.84	1.84	2.06	1.78	1.86	1.88	-	-	1.86	-	-	-	12.87
189	5.64	-	-	3.86	3.11	3.39	3.67	3.36	3.69	1.80	1.85	1.84	2.06	1.78	1.86	1.84	-	-	1.86	-	-	-	12.87
190	5.64	-	-	3.85	3.11	3.39	3.66	3.35	3.69	1.80	1.84	1.84	2.06	1.79	1.86	1.88	-	-	1.86	-	-	-	12.87
191	5.64	-	-	3.84	3.12	3.39	3.65	3.35	3.69	1.80	1.85	1.84	2.06	1.79	1.86	1.88	-	-	1.86	-	-	-	12.87
192	5.64	-	-	3.86	3.11	3.39	3.66	3.35	3.69	1.81	1.84	1.84	2.06	1.79	1.86	1.88	-	-	1.86	-	-	-	12.87
193	5.64	-	-	3.86	3.11	3.40	3.65	3.36	3.69	1.81	1.85	1.84	2.06	1.79	1.86	1.88	-	-	1.86	-	-	-	12.87
194	5.64	-	-	3.85	3.11	3.39	3.66	3.36	3.69	1.81	1.84	1.83	2.06	1.78	1.86	1.87	-	-	1.86	-	-	-	12.87
195	5.64	-	-	3.86	3.11	3.40	3.66	3.37	3.69	1.81	1.84	1.84	2.06	1.78	1.86	1.87	-	-	1.86	-	-	-	12.87
196	5.64	-	-	3.86	3.11	3.40	3.65	3.36	3.68	1.81	1.84	1.83	2.06	1.78	1.86	1.88	-	-	1.86	-	-	-	12.87
197	5.63	-	-	3.87	3.10	3.40	3.66	3.37	3.69	1.81	1.84	1.83	2.06	1.78	1.86	1.87	-	-	1.86	-	-	-	12.86
198	5.63	-	-	3.87	3.10	3.40	3.65	3.37	3.68	1.81	1.85	1.83	2.06	1.78	1.86	1.88	-	-	1.86	-	-	-	12.87
199	5.63	-	-	3.86	3.11	3.38	3.66	3.36	3.68	1.80	1.85	1.83	2.06	1.78	1.86	1.87	-	-	1.86	-	-	-	12.87
200	5.64	-	-	3.86	3.10	3.38	3.67	3.37	3.68	1.80	1.85	1.83	2.06	1.78	1.86	1.87	-	-	1.86	-	-	-	12.87
201	5.64	-	-	3.88	3.07	3.39	3.66	3.37	3.69	1.81	1.85	1.83	2.06	1.78	1.86	1.87	-	-	1.86	-	-	-	12.87
202	5.63	-	-	3.88	3.08	3.39	3.67	3.37	3.69	1.81	1.85	1.84	2.06	1.78	1.86	1.87	-	-	1.86	-	-	-	12.87
203	5.65	-	-	3.86	3.10	3.39	3.66	3.36	3.70	1.82	1.85	1.83	2.06	1.78	1.86	1.87	-	-	1.86	-	-	-	12.87

204	5.64	-	-	3.86	3.10	3.39	3.66	3.36	3.69	1.82	1.84	1.84	2.06	1.79	1.86	1.87	-	-	1.87	-	-	-	12.87
205	5.65	-	-	3.88	3.08	3.39	3.66	3.37	3.69	1.83	1.83	1.83	2.06	1.79	1.86	1.87	-	-	1.87	-	-	-	12.87
206	5.64	-	-	3.87	3.08	3.39	3.67	3.36	3.69	1.83	1.83	1.83	2.06	1.78	1.86	1.87	-	-	1.86	-	-	-	12.87
207	5.65	-	-	3.88	3.08	3.40	3.66	3.36	3.69	1.83	1.83	1.83	2.06	1.78	1.86	1.87	-	-	1.86	-	-	-	12.87
208	5.64	-	-	3.87	3.09	3.41	3.65	3.35	3.69	1.83	1.83	1.83	2.06	1.78	1.86	1.87	-	-	1.86	-	-	-	12.87
209	5.64	-	-	3.87	3.09	3.39	3.67	3.36	3.68	1.83	1.83	1.83	2.06	1.78	1.86	1.87	-	-	1.86	-	-	-	12.87
210	5.64	-	-	3.88	3.09	3.38	3.68	3.37	3.67	1.82	1.84	1.83	2.06	1.79	1.85	1.87	-	-	1.86	-	-	-	12.87
211	5.64	-	-	3.87	3.09	3.39	3.66	3.36	3.68	1.80	1.86	1.84	2.06	1.79	1.86	1.87	-	-	1.86	-	-	-	12.87
212	5.65	-	-	3.88	3.09	3.40	3.66	3.37	3.68	1.80	1.86	1.83	2.06	1.79	1.86	1.87	-	-	1.86	-	-	-	12.87
213	5.64	-	-	3.88	3.08	3.39	3.67	3.37	3.67	1.80	1.86	1.83	2.06	1.79	1.86	1.87	-	-	1.87	-	-	-	12.86
214	5.64	-	-	3.87	3.09	3.40	3.66	3.37	3.68	1.81	1.86	1.83	2.06	1.79	1.86	1.87	-	-	1.87	-	-	-	12.86

ELF analysis of ruthenium tetroxide

The ELF analysis corresponding to the RuO_4 moiety considers a total of 40 electrons. From these 40 electrons, 8 electrons correspond to the valence core of ruthenium(VIII), being possible to identify such electrons with $4s^2 4p^6$ electronic levels (not represented in Figure 4 of the main article). Now, it remains to locate 32 electrons.

According to the classical notation each oxygen atom should have 4 electrons and the double bond formed with ruthenium should also have 4 electrons (2 electrons for Ru and 2 electrons for each oxygen atom- In that way, it is possible to explain the observed valences: Ru shares 2 × 4 electrons = 8 (corresponding to a valence of +8) and each oxygen shares 2 electrons (corresponding to a valence of −2). The total is (4 + 4) × 4 = 32 electrons.

On the other hand, the ELF analysis assigns 4 additional electrons to the valence layer of Ru since these 4 electrons are located in a monosynaptic basing (assigning to the metal a total of 4 C(Ru) + 8 V(Ru) = 12 electrons, in contrast to the classical view that assign 16 shared electrons through the corresponding bonds). The remaining 28 electrons are completely assigned to the oxygen atoms (4 × 7 = 28 electrons). In agreement with this electronic distribution, more in line with the real situation of a nude highly oxidized metal, there are not covalent bonds between ruthenium and oxygen atoms. The ELF analysis also is consistent with the high electronegativity of the oxygen atoms, by assigning to their monosynaptic basins an “extra” electron. Because of these reasons, the ELF analysis showed in the main article (Figure 4) shows no disynaptic basins corresponding to Ru–O covalent bonds.

Cartesian coordinates

EP1

0 1

C	-2.8548691578	0.9395373692	-0.7345813962
H	-3.5727649136	1.7153706797	-1.0431066344
H	-1.9862766602	1.0102693433	-1.4091605032
C	-2.3884046101	1.0997148307	0.7333415694
H	-1.4465844213	1.6666154491	0.7951372463
O	0.4959158503	0.6066242852	-1.3885746980
O	3.0860209521	0.0512631778	-0.6028537095
O	1.2547238083	1.0475649190	1.1948008292
O	1.0204515315	-1.5304498055	0.2616038309
Ru	1.4698504173	0.0418624240	-0.1368226860
H	-3.1311281428	1.6604900468	1.3219739655
C	-2.2325646554	-0.3454163213	1.2901884385
H	-2.9861598836	-0.5337430270	2.0715258172
H	-1.2486069695	-0.5145375344	1.7546635168
C	-2.4792229021	-1.2819931736	0.0907978509
H	-2.8766675374	-2.2650709881	0.3883169209
H	-1.5341961441	-1.4653852424	-0.4486641088
C	-3.4304228705	-0.4821736807	-0.8078563801
H	-3.4941508214	-0.8728978400	-1.8359063843
H	-4.4501488697	-0.5017719119	-0.3831684852

EP2

0 1

C	-2.7154285606	1.1423535944	-0.4359761192
H	-3.3829912771	2.0040248683	-0.5841588830
H	-1.7424605856	1.3686405128	-0.9173171697
C	-2.4977335166	0.7636661433	1.0440413850
H	-1.5351631077	1.1449668244	1.4140463438
O	0.5781331321	1.1838244632	-0.8580030352
O	2.8861725146	-0.2979780828	-0.8354948423
O	1.7494696658	0.5324282379	1.5425063022
O	0.5387585792	-1.4215290756	0.0345519699
Ru	1.4426023398	-0.0001422490	-0.0247979860
H	-3.2871372730	1.1857187760	1.6824901132
C	-2.5595367901	-0.7852008884	1.0351160214
H	-3.4374747161	-1.1434408936	1.5925571951
H	-1.6662572336	-1.2507718654	1.4743574939
C	-2.6943401580	-1.1237384390	-0.4584372189
H	-3.3182254900	-2.0054800105	-0.6662292470
H	-1.6910845051	-1.2968158756	-0.9018677515
O	-3.3069550179	0.0087049596	-1.0507955717

EP3

0 1

C	-2.2516848787	0.4588740230	-1.4643946460
H	-3.0092647143	-0.0811273953	-2.0538867608
H	-1.5750912695	0.9867117432	-2.1486526839
C	-2.8795561914	1.3375645950	-0.3848858923
H	-2.1384676086	2.0712815348	-0.0315242698
O	0.3429264692	0.7944646825	-0.4995346799
O	0.7670832437	-1.3850210350	0.6109671576
O	2.9947935781	-0.1512398442	-0.5420395347
O	1.7228187979	0.8546436576	1.9465859645
Ru	1.6352077216	0.1047653435	0.4317210030
H	-3.7423294935	1.8909928187	-0.7850840396
C	-3.2750540103	0.3971557739	0.7581265044
H	-4.1321915120	-0.2256461314	0.4556070728
H	-3.5643330847	0.9445984302	1.6678214464
C	-2.0609334756	-0.4877500490	1.0380426231
H	-2.2912988930	-1.4472330003	1.5197158027
H	-1.3007403383	0.0320526327	1.6403107500
S	-1.2867553404	-0.8423997800	-0.5918608175

EP4a

0 1

C	-2.3447082368	-0.0376201459	1.1706634258
H	-2.8925364633	0.3497509538	2.0468039458
H	-1.2533104097	-0.0271213139	1.4462769381
C	-2.7575394359	-1.4398547177	0.7243714658
H	-2.2940692421	-2.2284697925	1.3336398734
O	0.6754960543	-0.2581198888	1.4515247460
O	3.0721912860	0.6559755542	0.4130233377
O	1.6086529353	-1.3989349861	-0.8720139110
O	0.6889358411	1.2377646397	-0.8432366565
Ru	1.5479851896	0.0391059911	0.0163790550
H	-3.8517849697	-1.5534294822	0.7935038756
C	-2.2995723998	-1.4612838171	-0.7433764103
H	-2.8413061547	-2.1945458805	-1.3571192887
H	-1.2272319670	-1.7074966875	-0.7979095186
C	-2.5244916142	-0.0156774134	-1.2175247781
H	-3.4759315224	0.0961966033	-1.7713188502
H	-1.7188611169	0.3386638990	-1.8835996283
C	-2.0670575623	2.1134720973	-0.0096931238
H	-2.3095443745	2.6326623299	0.9298575229
H	-0.9484598904	2.1121731295	-0.1311170080
H	-2.4854809868	2.6796471556	-0.8561463281
N	-2.5620479600	0.7791877725	0.0080913156

EP4b

0 1

C	2.0671582944	2.1134576589	-0.0098185745
H	0.9485301137	2.1121783479	-0.1309909257
O	-0.6889335846	1.2377943647	-0.8430034106
O	-3.0722237582	0.6560075491	0.4132255157
O	-1.6087202949	-1.3989351830	-0.8716682356
O	-0.6755460564	-0.2579425147	1.4518409939
Ru	-1.5479814294	0.0391574065	0.0166458939
H	2.4854223290	2.6795908664	-0.8563774821
H	2.3098539627	2.6326776933	0.9296625656
C	2.3448206659	-0.0376081950	1.1705342990
C	2.2995368578	-1.4613496477	-0.7434428676
C	2.7576094676	-1.4398616172	0.7242702797
H	1.2534417061	-0.0270099914	1.4461700819
H	2.8927091346	0.3497670123	2.0466341522
H	1.2272073308	-1.7076165917	-0.7978767538
H	2.8412586376	-2.1946077905	-1.3572011539
H	2.2941895096	-2.2284588269	1.3336002542
H	3.8518604641	-1.5534237907	0.7933326951
N	2.5621254975	0.7791584130	0.0079231544
C	2.5243550164	-0.0157482281	-1.2176663294
H	1.7186001880	0.3385603576	-1.8836068039
H	3.4756919479	0.0961277071	-1.7716343484

EP5a

0 1

C	-2.3928290089	0.0122384812	1.2474041978
H	-3.0031279487	0.4290083897	2.0650141214
H	-1.3228581550	0.1646780614	1.5482646065
C	-2.6660669867	-1.4603248306	0.9387277025
H	-2.1374712355	-2.1360417614	1.6257762113
O	0.6423012325	0.3867395770	1.5905210042
O	2.9662299466	1.1886427837	0.3220760091
O	1.6116777489	-1.1847170665	-0.4564884898
O	0.5510074544	1.3315098624	-0.9874034221
Ru	1.4821548021	0.4066888749	0.1037050089
H	-3.7451944795	-1.6709283439	1.0180614494
C	-2.1956705874	-1.5865790935	-0.5181955319
H	-2.6436379633	-2.4372977396	-1.0507250071
H	-1.1013081418	-1.7067762951	-0.5496556728
C	-2.5834660363	-0.2348463922	-1.1350706189
H	-3.5676125319	-0.2822450448	-1.6394736409
H	-1.8521317728	0.1168316348	-1.8815593480
C	-2.2239371142	2.0427388342	-0.1845087531
H	-1.1086492361	2.0574036459	-0.3495254040
H	-2.6635237285	2.4229495546	-1.1214114185

N	-2.6453523765	0.6865006408	-0.0023936100
C	-2.5746887664	2.9396006801	0.9763012184
C	-1.6324435928	3.2654949627	1.9552661128
C	-3.8847523750	3.4275878885	1.1250855851
C	-1.9681582942	4.0588580288	3.0590987008
H	-0.6131770989	2.8816799552	1.8658542160
C	-4.2366844200	4.2161795687	2.2138965807
H	-4.6397566126	3.1794580637	0.3743583983
C	-3.2792445469	4.5390466247	3.1939464549
H	-1.2054047578	4.2903219742	3.8017129262
H	-5.2525505625	4.5986347083	2.3322540946
O	-3.7117544216	5.3112000864	4.2202006831
C	-2.8008845790	5.6623913430	5.2451795131
H	-1.9527611517	6.2485295688	4.8518135852
H	-3.3595601258	6.2766764808	5.9626541933
H	-2.4086668962	4.7699980230	5.7624743537

EP5b

0 1

C	0.6270841554	0.8053959173	-1.1425633049
H	-0.0528848759	-0.0874397634	-1.0545202173
O	-1.1940807159	-1.3359098182	-0.0736168962
O	-3.6816511340	-2.3430134144	-0.7050905069
O	-3.3581551246	-0.6636416791	1.5400812472
O	-3.1256077503	0.3675736314	-1.0018269086
Ru	-2.8701491487	-1.0170040925	-0.0386893079
H	0.6000422391	1.1058318391	-2.2044361589
C	-1.0579685375	2.5834051983	-0.8417386936
C	-1.2102904005	2.4488440283	1.5369807525
C	-1.5414623500	3.3869627811	0.3653116995
H	-1.8411367380	1.8404134647	-1.1470992660
H	-0.8251371425	3.1878681118	-1.7348090450
H	-1.9999054591	1.6887546693	1.6491486712
H	-1.1120884118	2.9721897700	2.4984909713
H	-2.6093565418	3.6388607643	0.2999560729
H	-0.9737054041	4.3280910784	0.4486793709
N	0.1043156784	1.8803190299	-0.3597673808
C	0.0987729277	1.7734813378	1.1004813861
H	0.1585804254	0.7169267426	1.4100561967
H	0.9841846538	2.2817144896	1.5238519679
C	2.0226453968	0.3961593149	-0.7358219868
C	3.0478068058	1.3557370062	-0.6524071197
C	2.3327705664	-0.9309732132	-0.4290650566
C	4.3382699515	0.9951969482	-0.2858469677
H	2.8194669687	2.4012032255	-0.8745875906
C	3.6291690635	-1.3136066103	-0.0613913307
H	1.5446278909	-1.6869841766	-0.4676240644
C	4.6425902723	-0.3469515674	0.0117557742

H	5.1373328385	1.7366506078	-0.2213011659
H	3.8308897581	-2.3587113305	0.1709893830
O	5.9270713214	-0.6054937452	0.3592834073
C	6.2979646694	-1.9348166339	0.6729621443
H	7.3671869131	-1.9112656932	0.9190998956
H	5.7362410876	-2.3190596482	1.5417270708
H	6.1395271508	-2.6134605699	-0.1827740430

IN4a

0 1

C	-2.1194025864	0.1486492413	1.1350769510
H	-2.0558483131	0.7284925313	2.0581576760
H	0.4045362359	1.4180209170	1.0251462975
C	-2.0809454713	-1.3291716262	1.0235615131
H	-1.0529306878	-1.6463776569	1.2703408040
O	0.7496785708	0.6000981413	1.4607541716
O	3.2121490481	0.0867500635	-0.0963202298
O	0.6558292561	-1.5284847422	-0.5888414454
O	0.7542691613	1.2613566455	-0.8732008941
Ru	1.5328919874	-0.1584622639	-0.0820230698
H	-2.7543850335	-1.8064927977	1.7507639676
C	-2.4380987542	-1.5813724117	-0.4586094469
H	-3.4913522425	-1.8819640819	-0.5484673242
H	-1.8072965673	-2.3588345511	-0.9021727015
C	-2.2099238182	-0.2241952025	-1.1394946033
H	-2.9912456931	0.0669747935	-1.8539866349
H	-1.2234247767	-0.1300127414	-1.6170428114
C	-2.1656476312	2.1652509284	-0.2559079104
H	-2.2586257698	2.7102737712	0.6905820203
H	-1.1905110165	2.3718522015	-0.7222910615
H	-2.9856327579	2.4382891868	-0.9332304742
N	-2.2147631401	0.7319656543	-0.0057297937

IN5a

0 1

C	-0.2947587291	1.7371696328	0.9255071433
H	0.6023466897	1.2718946712	1.3352232399
H	-0.1229650220	-0.9692213791	0.3870651558
C	-1.4889893222	2.1739649572	1.6899222287
H	-2.0022346382	1.2618302932	2.0386180304
O	-0.7922381777	-1.0940281469	1.0979930606
O	-2.7440888297	-2.9565827483	-0.0723743382
O	-3.3431647560	0.0468495071	0.2110310914
O	-1.1990948450	-0.9420230285	-1.3263721506
Ru	-2.3306748285	-1.3128770308	0.0260467168
H	-1.2043891442	2.7496621973	2.5830960224

C	-2.3116569067	2.9617510988	0.6458608218
H	-2.1830744206	4.0424808651	0.7978418860
H	-3.3794379000	2.7231972286	0.7015095749
C	-1.7170103255	2.5374631864	-0.7052978469
H	-1.5502982889	3.3616367888	-1.4116009545
H	-2.2934226368	1.7424475957	-1.1990543865
C	0.5391816220	1.5707423184	-1.4031820710
H	-0.0138270044	0.8509772809	-2.0230310113
H	0.7196714902	2.4864120044	-1.9862152770
N	-0.4081634545	1.9492902579	-0.3357831100
C	1.8174081954	0.9729140509	-0.8901363724
C	2.0010525083	-0.4153060775	-0.8871156946
C	2.8476201635	1.7878855483	-0.3876033741
C	3.1754639033	-0.9913597408	-0.3937488244
H	1.2000294645	-1.0501341077	-1.2741278128
C	4.0181345013	1.2294811465	0.1106499488
H	2.7292515179	2.8747376789	-0.3860596598
C	4.1929102465	-0.1678940542	0.1138913918
H	3.2845752732	-2.0749745165	-0.4104743408
H	4.8220240411	1.8557103660	0.5018731670
O	5.3626375921	-0.6213109819	0.6158454020
C	5.6036687492	-2.0176702697	0.6476401696
H	6.5983159543	-2.1515778497	1.0908520756
H	4.8573620279	-2.5426798055	1.2676100764
H	5.5977582901	-2.4520749370	-0.3663439784

IN5b

0 1

C	0.2950610000	3.1066510000	-0.5666020000
H	-0.2065180000	2.6655810000	-1.4389720000
H	2.0152220000	0.0177680000	-1.5551880000
C	1.6577780000	3.7227080000	-0.8868830000
H	2.1498330000	3.1412410000	-1.6825370000
O	2.8415110000	-0.2766780000	-1.0905450000
O	2.4898520000	-3.1726340000	-0.6535600000
O	1.9334210000	-1.1436380000	1.5898280000
O	0.5640540000	-1.0888480000	-0.8482960000
Ru	2.0705880000	-1.6462200000	-0.0404200000
H	1.5657450000	4.7622670000	-1.2281930000
C	2.4253830000	3.5714320000	0.4311680000
H	2.0999860000	4.3344810000	1.1560970000
H	3.5137110000	3.6552890000	0.3101590000
C	2.0148860000	2.1770840000	0.8957500000
H	2.0485850000	2.0214610000	1.9806760000
H	2.6103740000	1.3995190000	0.3935560000
C	-0.1446210000	1.0531440000	0.7789500000
H	0.3548560000	0.3370380000	1.4414740000
N	0.6111430000	2.0474620000	0.4141740000

C	-1.5156440000	0.7700980000	0.4545820000
C	-1.9706110000	-0.5186520000	0.8185310000
C	-2.4345210000	1.6477790000	-0.1748690000
C	-3.2647360000	-0.9393910000	0.5505770000
H	-1.2751190000	-1.2031510000	1.3054610000
C	-3.7299820000	1.2416560000	-0.4348830000
H	-2.1521400000	2.6619840000	-0.4435130000
C	-4.1605740000	-0.0575990000	-0.0867560000
H	-3.5722580000	-1.9431820000	0.8384390000
H	-4.4450620000	1.9140460000	-0.9109210000
O	-5.4293860000	-0.3558980000	-0.3895150000
C	-5.9433000000	-1.6513420000	-0.1028790000
H	-6.9789910000	-1.6558090000	-0.4624790000
H	-5.3698430000	-2.4317360000	-0.6283950000
H	-5.9316680000	-1.8545650000	0.9794980000
H	-0.3809030000	3.8393410000	-0.0993000000

P1

0 1

C	-1.3969196912	0.4049751798	0.6534915913
H	-1.1016438207	0.6409964864	1.6903549203
H	0.2049121863	-0.8202311101	1.8233597971
C	-1.8958164556	-1.0566432109	0.4692639309
H	-1.1925581439	-1.5903613477	-0.1877096995
O	1.1141628176	-0.9362887165	1.5006037077
O	2.3831079611	1.3496680338	0.4287427171
O	1.5823448079	-1.0897883240	-1.2921209798
O	-0.3084602302	0.7145888737	-0.2264967839
Ru	1.4372233738	0.0515824224	-0.0664862097
H	-1.9281643525	-1.6029294024	1.4240681271
C	-2.5735063490	1.2917624925	0.2087515323
H	-3.2235000961	1.4680377017	1.0818450113
H	-2.2208925469	2.2676896762	-0.1543944888
C	-3.2901982909	-0.9535878069	-0.1891339241
H	-4.0744351773	-1.0116599050	0.5834427486
H	-3.4763484546	-1.7666013370	-0.9062544944
C	-3.3089405676	0.4405631470	-0.8303114161
H	-4.3233707723	0.8054758589	-1.0506241749
H	-2.7408851982	0.4336212880	-1.7757479126

P2

0 1

C	1.4418408290	0.7310953384	0.6315797866
H	1.2099778568	1.2381655592	1.5819220229
H	0.1455281171	-1.2305335396	1.2364345522
C	2.6887378000	1.2348999997	-0.0878072077

H	2.4816322406	2.1310672131	-0.6863401099
O	-0.8384339330	-1.2629094978	1.1298578020
O	-1.5275287382	-0.8013983201	-1.5671378956
O	-2.3087202969	1.1171247809	0.7171353986
O	0.3479658734	0.8886448236	-0.2556578349
Ru	-1.3338853443	0.0359672342	-0.1233858544
H	3.4573350610	1.4786998733	0.6620317450
C	3.0796571466	0.0029650259	-0.9144349642
H	4.1412003816	-0.0083125028	-1.1962374329
H	2.4777276849	-0.0394378623	-1.8338779172
C	2.7152316696	-1.1536571350	0.0203542102
H	3.5684413409	-1.4814065166	0.6345356343
H	2.2976708298	-2.0260058201	-0.5026420696
O	1.7111404811	-0.6229186540	0.9220081346

P3

0 1

C	1.2929142870	0.4071838498	0.9658707882
H	1.0755778028	0.3297237251	2.0431275128
H	-0.0823333893	-1.8127229202	0.0675900031
C	2.4327438810	1.3705998445	0.6264562113
H	2.0847086344	2.4101783453	0.7135252844
O	-1.0647923075	-1.7208414220	0.0302500493
O	-1.7564670557	0.3316193199	-1.7443130663
O	-2.4541401846	0.4008630529	1.2641716297
O	0.1553373567	0.8848678783	0.2849940702
Ru	-1.5162486007	0.0907720022	-0.0974070834
H	3.2519989377	1.2165429316	1.3463970642
C	2.8693461846	1.0130569134	-0.7973776114
H	3.7988061517	1.5276284608	-1.0830062729
H	2.0831798641	1.3243659293	-1.5010360446
C	3.0507562823	-0.5107153810	-0.8670936617
H	4.0750349023	-0.8184942540	-0.6147923831
H	2.7885389098	-0.9273119305	-1.8480012647
S	1.9468783435	-1.2381683453	0.4336737749

P4a

0 1

C	-1.3351987545	-0.2834670345	0.6806717843
H	-0.8496706103	-0.1268099879	1.6520519364
H	2.0176494565	1.0237921432	1.9299689499
C	-2.2835118167	-1.4786996757	0.6133700884
H	-1.7415876443	-2.4305898576	0.5299373330
O	1.1909708271	0.6690924390	1.5573150335
O	2.3043009820	-1.5817939240	0.1336359507
O	-0.2879287450	-0.5327801943	-0.3132243825

O	1.8259883168	1.1492697121	-1.1974727744
Ru	1.5222577874	-0.1153840025	-0.1288106629
H	-2.8823443410	-1.5002337071	1.5372360219
C	-3.1506875830	-1.1469966171	-0.6144433980
H	-4.1572140279	-1.5839126292	-0.5535655838
H	-2.6712192051	-1.5400989565	-1.5226753920
C	-3.1735585117	0.3969518490	-0.6576482066
H	-4.1582936260	0.8147644961	-0.3791062932
H	-2.9440726488	0.7730279690	-1.6738875747
C	-1.6092469104	2.1372439341	0.2118138481
H	-0.8690463054	2.2918577140	1.0098300218
H	-1.1060841331	2.3249711222	-0.7589382350
H	-2.4061092317	2.8912377807	0.3247810568
N	-2.1621412748	0.8082454272	0.3148054783

P4b

0 1

C	-0.7903049471	1.7243997865	0.2334829726
H	0.7925420965	0.7565369809	1.9750647123
O	1.3040851056	-0.0319388312	1.7270601085
O	3.0275742004	0.3816391038	-0.4914685095
O	0.6442587123	-1.5842052748	-0.5667953861
O	0.3018660217	1.2066234735	-0.5822703678
Ru	1.4828706201	-0.1878105291	-0.1441523592
H	-0.4498909571	1.9534538222	1.2574244168
H	-1.0109840183	2.6820197838	-0.2682141027
C	-2.6217521307	0.6201964147	-1.0030237693
C	-2.7312456013	-1.3220498933	0.3847747392
C	-3.5567357518	-0.5357885942	-0.6424089575
H	-1.9133203078	0.3116702916	-1.7934722127
H	-3.1582712789	1.5132170122	-1.3669021064
H	-1.9578532038	-1.9130566606	-0.1313930195
H	-3.3321766627	-2.0048051160	1.0029110102
H	-3.8479433939	-1.1317652706	-1.5194444155
H	-4.4770552238	-0.1483919203	-0.1746514725
N	-1.9270520359	0.9059867473	0.2583109109
C	-2.0578924241	-0.2153380859	1.2028524063
H	-1.0842545704	-0.5319205119	1.6055971524
H	-2.6866762490	0.0799412712	2.0642142493

P5a

0 1

C	-1.5797092712	0.8416708508	0.5200222107
H	-0.6498156000	0.6709700236	1.0719153599
H	0.8188176331	-1.9930469437	1.3785296706
C	-2.8290344091	0.9732987491	1.3867928091

H	-3.1816894762	-0.0024355650	1.7487781779
O	0.1963261684	-1.2980931644	1.1007537134
O	-2.0575695068	-2.9699987823	0.4641773221
O	-1.7735746878	-0.2823179774	-0.3963542077
O	0.0158700774	-2.1080941423	-1.6454437106
Ru	-0.9338167697	-1.9493438542	-0.2618786040
H	-2.5874442173	1.5991106657	2.2602613479
C	-3.8181408196	1.6799565346	0.4428308350
H	-4.5662338990	2.2795687525	0.9799799758
H	-4.3578372423	0.9311859859	-0.1550421084
C	-2.9196811983	2.5361731975	-0.4762392736
H	-2.9889465066	3.6161355578	-0.2520736609
H	-3.1991962551	2.4118445691	-1.5400213016
C	-0.5404247507	2.3008876172	-1.2129551612
H	-0.7678998544	1.7783081179	-2.1641404782
H	-0.5502511078	3.3818594762	-1.4386789877
N	-1.5628091419	2.0584421675	-0.2104355957
C	0.8248946239	1.8818035297	-0.7248925485
C	1.4987949708	0.8041956017	-1.3003357827
C	1.4133457713	2.5150390793	0.3845182323
C	2.7238719186	0.3492452435	-0.7980586871
H	1.0489976119	0.2774274712	-2.1441184148
C	2.6284581757	2.0787372824	0.8985318702
H	0.8958821102	3.3518030429	0.8610685978
C	3.2941948829	0.9847665961	0.3128053590
H	3.2083434788	-0.5047193866	-1.2700458759
H	3.0867750091	2.5646896402	1.7624646921
O	4.4675490726	0.6188771772	0.8880273311
C	5.1644212765	-0.4954263362	0.3641897479
H	6.0646115587	-0.6201702301	0.9795238402
H	4.5582192523	-1.4162366296	0.4202814556
H	5.4649181216	-0.3313659175	-0.6850921497

P5b

0 1

C	-0.3930268188	0.8900087092	-1.1190101955
H	0.4518634859	-0.5671546088	0.9788654301
O	-0.1666791556	-1.2354533384	1.3354934847
O	-0.8594437202	-3.1557654468	-0.6204705465
O	-2.9259950162	-1.2279721809	0.6819406726
O	-1.0530616274	-0.4136993883	-1.2513060850
Ru	-1.4459293475	-1.7205313835	0.0417103098
H	-0.3651656649	1.1767814818	-2.1866117004
C	-2.4169096439	2.2786738057	-1.1230717552
C	-2.8315101855	2.2026623269	1.2208855757
C	-3.1845584938	3.0176853611	-0.0287436104
H	-2.9998389947	1.4102992306	-1.4810396774
H	-2.1878297481	2.9109147757	-1.9971167898

H	-3.4113611469	1.2663387651	1.2325523546
H	-3.0187722811	2.7368780291	2.1637047284
H	-4.2641464833	3.0674021956	-0.2313575573
H	-2.8081894588	4.0494984061	0.0692459647
N	-1.1793802167	1.8517587057	-0.4532016314
C	-1.3480063433	1.8794692077	1.0128033140
H	-1.0620696684	0.9292614045	1.4837182768
H	-0.7119636237	2.6639188098	1.4607598338
C	1.0383503982	0.7725900600	-0.6310418442
C	1.6768977850	1.8605158926	-0.0082734000
C	1.8006652253	-0.3724051593	-0.9043799971
C	3.0134195284	1.7886449862	0.3633294192
H	1.1150792781	2.7767681525	0.1725819498
C	3.1447647870	-0.4612168974	-0.5309287122
H	1.3406735331	-1.2189041798	-1.4167913466
C	3.7616690073	0.6238384673	0.1117322933
H	3.5081480866	2.6307257715	0.8508761704
H	3.6974258533	-1.3733301226	-0.7517493465
O	5.0518383194	0.6423886924	0.5117413562
C	5.8629990110	-0.4957051251	0.2758003459
H	6.8570613801	-0.2569645431	0.6739609723
H	5.4696339610	-1.3865168573	0.7940872433
H	5.9480129992	-0.7151380062	-0.8019165000

R1

0 1

C	-0.3629795481	-1.2340437674	0.1401796861
C	-1.3058569073	-0.0066618853	0.0028131921
C	-0.3761087743	1.2290497395	-0.1440478476
C	1.0232548421	0.7295863233	0.2535607641
C	1.0320348203	-0.7178399373	-0.2525104009
H	-0.3422036280	-1.5764063147	1.1880864817
H	-0.6875805273	-2.0914268852	-0.4694107345
H	-1.9595731322	0.0849029212	0.8840479526
H	-1.9694904145	-0.1039404630	-0.8703309514
H	-0.3574427223	1.5623531879	-1.1949832082
H	-0.7102499783	2.0882693496	0.4577830832
H	1.8367069845	1.3463328219	-0.1603226633
H	1.1314585734	0.7365267563	1.3527890346
H	1.1441031626	-0.7229746938	-1.3513142568
H	1.8510732496	-1.3255241531	0.1637248686

R2

0 1

C	1.1641059149	-0.4602037158	-0.0005522056
O	-0.0029443415	-1.2437479572	-0.2580808713

C	-1.1589730074	-0.4052706575	-0.3109665731
C	-0.7227740745	0.9544525530	0.2379471575
C	0.7470192921	0.9989539190	-0.1938380506
H	1.5118548677	-0.6375591780	1.0358568000
H	1.9699919618	-0.7744281210	-0.6845249624
H	-1.5073239257	-0.3150083898	-1.3583570522
H	-1.9701321764	-0.8687202676	0.2749015339
H	-1.3248154551	1.7863656594	-0.1555045907
H	-0.7983007147	0.9674815750	1.3377263353
H	0.8245718713	1.2837210942	-1.2560428940
H	1.3594467875	1.6989604863	0.3928993733

R3

0 1

C	0.0468870683	1.3438838382	0.1371085708
C	0.0496369336	-1.3437229925	-0.1361061093
C	-1.2831365873	-0.7150590474	0.2758596896
C	-1.2842722655	0.7126242643	-0.2761022190
H	0.0150148119	1.6998733762	1.1780489533
H	0.3411409622	2.1836309211	-0.5080136886
H	0.0194171574	-1.6997911376	-1.1770693000
H	0.3449200765	-2.1828895279	0.5093011257
H	-2.1318999043	-1.3081153292	-0.1003837741
H	-1.3573287119	-0.6872393407	1.3758992075
H	-1.3573849383	0.6846623654	-1.3762106651
H	-2.1345431981	1.3040226627	0.0993469089
S	1.3160095954	0.0013099474	0.0010753003

R4

0 1

C	-0.0216161839	-1.1539964696	0.1604103191
C	-1.4956172502	-0.7771325973	-0.0632003234
C	-1.4956154622	0.7771266576	-0.0632223585
C	-0.0216135539	1.1539934649	0.1603774154
N	0.7133554702	-0.0000094776	-0.3402404062
H	0.2830618251	-2.0750851826	-0.3626786071
H	-1.8505916225	-1.1634666465	-1.0301547968
H	-2.1433582820	1.2017673489	0.7174744003
H	0.1736424186	1.3135931528	1.2475339365
H	0.2830665753	2.0750665633	-0.3627377769
H	-1.8505891308	1.1634341022	-1.0301877257
H	-2.1433612093	-1.2017496455	0.7175083470
H	0.1736394028	-1.3135656206	1.2475713824
C	2.1240079699	-0.0000066806	-0.0338313584
H	2.3284927634	0.0000087552	1.0629790125
H	2.6088131380	-0.8910705779	-0.4638715107

H	2.6088151313	0.8910438532	-0.4638969495

R5

0 1

C	-2.4481941966	1.2077852129	0.3123852035
C	-3.6110336750	1.3398638249	-0.6874054055
C	-3.7201185463	-0.0674223426	-1.3383662260
C	-2.6018212148	-0.8835063695	-0.6672136852
N	-2.4248030106	-0.2163910823	0.6149129143
H	-2.5847005910	1.8013614632	1.2314668899
H	-4.5438922655	1.5955184211	-0.1634583091
H	-3.5998733345	-0.0400372500	-2.4312306624
H	-1.6805523616	-0.8451185931	-1.2896870402
H	-2.8541601553	-1.9472162611	-0.5242123325
H	-4.7026918755	-0.5143635722	-1.1263023323
H	-3.4198653442	2.1336515716	-1.4242382693
H	-1.5016159805	1.5497513490	-0.1614661762
C	-1.3522212099	-0.6978513050	1.4770043971
H	-1.4289925935	-0.1588286542	2.4360638968
H	-1.5465560299	-1.7630885463	1.6867077045
C	0.0545525341	-0.5438150973	0.9270892371
C	0.7970628512	0.6219214392	1.1494631718
C	0.6348132467	-1.5427341836	0.1252231073
C	2.0703060798	0.8053696388	0.5974324762
H	0.3783010848	1.4127581543	1.7780967336
C	1.8992730456	-1.3812095973	-0.4333884963
H	0.0859945623	-2.4705837778	-0.0583090167
C	2.6288953789	-0.2013169983	-0.2045425773
H	2.6134369336	1.7276773062	0.8013792641
H	2.3496272104	-2.1603629477	-1.0519929149
O	3.8522990614	-0.1276724136	-0.7875418371
C	4.6342975917	1.0348838920	-0.5910708082
H	5.5684652932	0.8816216062	-1.1464331193
H	4.1284999183	1.9361218127	-0.9786597344
H	4.8710215927	1.1911323000	0.4756479468

RuO4

0 1

Ru	0	0.	0.	0.
O	0	0.9708090000	0.9708090000	0.9708090000
O	0	-0.9708090000	-0.9708090000	0.9708090000
O	0	0.9708090000	-0.9708090000	-0.9708090000
O	0	-0.9708090000	0.9708090000	-0.9708090000

TS1

0 1

C	1.8540451420	0.0515212257	1.0642158987
H	1.8741079935	0.0848461217	2.1664831688
H	0.5269761185	0.1559617674	1.1893001408
C	2.4371856101	1.2558879824	0.3410895374
H	1.6537149610	1.7922875897	-0.2182677263
O	-0.6634615708	0.3028163888	1.4894025448
O	-2.3094319232	-1.4979017988	0.0287582078
O	-2.2147205056	1.3558021339	-0.6830639251
O	0.0231848236	-0.2642465932	-0.8196844647
Ru	-1.4908683866	-0.0238604683	-0.0339818958
H	2.8664618939	1.9819727550	1.0493925232
C	2.4127219399	-1.2151063462	0.4509586145
H	3.2290438601	-1.5700390173	1.1099510143
H	1.6825288510	-2.0364885444	0.3930514996
C	3.4836391015	0.6551054393	-0.6270112138
H	4.4632330120	0.6108993976	-0.1219249611
H	3.6098149192	1.2583776193	-1.5381928200
C	2.9831463908	-0.7716914166	-0.9045538703
H	3.7760772882	-1.4406758398	-1.2714732544
H	2.1786464808	-0.7531623963	-1.6543400185

TS2

0 1

C	-1.9802765095	0.1842408097	1.1026546534
H	-1.9768261391	0.3924505681	2.1847072289
H	-0.6437338954	0.2176603174	1.1169738660
C	-2.4435011306	-1.1520517210	0.5814125323
H	-1.6701226267	-1.9282818988	0.6507049780
O	0.5374108474	0.1682168234	1.4792185418
O	2.2970951560	1.3979937034	-0.4013188006
O	2.0919123243	-1.5469034970	-0.1766138602
O	-0.0755791983	0.0202817445	-0.9311792111
Ru	1.4210965341	-0.0031404208	-0.0489355520
H	-3.2958674105	-1.4787096705	1.2033615845
C	-2.8719663482	-0.8255951208	-0.8629592523
H	-3.8329756618	-1.2936701392	-1.1141275682
H	-2.1151463904	-1.1739135421	-1.5748749746
C	-2.9623971590	0.7106529021	-0.8953444763
H	-3.9793681050	1.1016227374	-1.0392163665
H	-2.2900087949	1.1621666743	-1.6376961085
O	-2.5332924924	1.1786637301	0.4089847852

TS3

0 1

C	1.8317725867	0.0040782524	1.1702659687

H	1.9316042406	-0.1991214733	2.2459304791
H	0.5085497234	-0.1703133892	1.1823804499
C	2.1835634136	1.4015031040	0.6652483546
H	1.2677228729	1.9998025566	0.5614182608
O	-0.6766150438	-0.3971789964	1.3953290678
O	-2.2042494349	-1.5333292849	-0.7013076437
O	-2.7139672045	1.2035526169	0.2732744334
O	-0.3634183880	0.5070642579	-1.0309476744
Ru	-1.6570375913	-0.0785130192	-0.0396099103
H	2.8151928054	1.9093884423	1.4110222458
C	2.9247909302	1.2493836578	-0.6832109769
H	4.0105640879	1.3151679427	-0.5140995240
H	2.6485698169	2.0445923668	-1.3912891749
C	2.5890553360	-0.1230421570	-1.2696483781
H	3.3306473782	-0.5080710601	-1.9817998107
H	1.5826367250	-0.1360550572	-1.7158139714
S	2.5142597455	-1.2470677599	0.1741368046

TS4a

0 1

C	-2.1019617472	0.0263618716	1.1462261895
H	-2.2681781424	0.4851861560	2.1339149427
H	-0.8670092292	-0.1247819829	1.1518780032
C	-2.6480776634	-1.3644100784	0.8590329606
H	-2.0773884808	-2.1511012511	1.3704090158
O	0.4141863975	-0.6663062796	1.1508235668
O	2.9548144923	0.4923259143	0.7503180106
O	1.6858802749	-1.2102380186	-1.2733997050
O	0.7396083739	1.3337987275	-0.6499197085
Ru	1.5605738415	-0.0668776742	-0.0339266834
H	-3.6989463958	-1.4339166331	1.1880193470
C	-2.5278892221	-1.4259093058	-0.6743482953
H	-3.2326725858	-2.1276747867	-1.1395527861
H	-1.5076609830	-1.7329113886	-0.9515008693
C	-2.7636727439	0.0262523668	-1.1199218135
H	-3.8088902711	0.2145425808	-1.4249684665
H	-2.1142866036	0.3353686643	-1.9552581313
C	-2.0370173143	2.1754026125	-0.0371676385
H	-2.1854263597	2.7097823328	0.9126078445
H	-0.9367360798	2.1643854625	-0.2674297830
H	-2.5739728399	2.6894823336	-0.8457532737
N	-2.4631647180	0.8201313765	0.0721382734

TS4b

0 1

C	1.4895306531	2.0355275378	-0.0467659434

H	0.3489881440	1.6482050859	-0.3562207907
O	-0.7223068728	1.0435570793	-1.0224961635
O	-3.1317378406	0.3849126370	0.2560751785
O	-1.2318287511	-1.6986505465	-0.5879234824
O	-0.6338449773	0.0908440463	1.4407810653
Ru	-1.5421607282	-0.1337792433	-0.0188441256
H	1.6934723617	2.7042500706	-0.8962945977
H	1.3925721343	2.5653600366	0.9086421132
C	2.3876610568	0.1293184154	1.2148746153
C	2.5657690744	-1.2893745631	-0.7107185028
C	3.0077426367	-1.1946041789	0.7577286000
H	1.3424933970	0.0003451459	1.5781585111
H	2.9516234014	0.6468892391	2.0083750913
H	1.5188720209	-1.6286329382	-0.7713851792
H	3.1870577488	-1.9667241542	-1.3127929887
H	2.6656014732	-2.0419095684	1.3678350878
H	4.1066256462	-1.1501464633	0.8262477819
N	2.3175734864	0.9391510754	0.0110290517
C	2.6576696176	0.1652826668	-1.1834146631
H	1.9711247049	0.4139269240	-2.0052156517
H	3.6846296127	0.4151176958	-1.5089300072

TS5a

0 1

C	-0.3449335128	1.8691494209	0.9620502137
H	0.6287364085	1.6552071733	1.4283450866
H	-0.8398485387	0.7380048622	0.9914684687
C	-1.3207565975	2.7924544744	1.6742062590
H	-1.8000068523	2.3081204418	2.5358651809
O	-1.4130730498	-0.5029157402	1.2903639251
O	-1.4382713789	-2.9318590977	-0.1432932398
O	-3.8537019414	-1.3114460836	0.2070486855
O	-1.7173283515	-0.5129121957	-1.4079191698
Ru	-2.1778712987	-1.4154866773	0.0036476704
H	-0.7922246833	3.6896087974	2.0395648032
C	-2.3027992572	3.1366685048	0.5425697613
H	-2.8323454155	4.0867581106	0.6930904253
H	-3.0536987205	2.3361713157	0.4531515714
C	-1.4181321854	3.1466966718	-0.7138508239
H	-1.0598932855	4.1591052328	-0.9737740442
H	-1.9226403341	2.7340924524	-1.6026154209
C	0.4046498261	1.5574725364	-1.3691641085
H	-0.3105707842	0.7416140267	-1.6647268053
H	0.5530235630	2.2088868542	-2.2453435493
N	-0.2728184406	2.3105342591	-0.3515899366
C	1.6941763485	0.9360161682	-0.8972681959
C	1.8147618906	-0.4503090481	-0.7693757226
C	2.7925739771	1.7366719116	-0.5355916899

C	2.9903431965	-1.0420407275	-0.2929770314
H	0.9748232163	-1.0897748808	-1.0464024950
C	3.9658127127	1.1666342634	-0.0571276079
H	2.7198886156	2.8239587342	-0.6232853463
C	4.0749125443	-0.2315038068	0.0720107188
H	3.0436841140	-2.1267404276	-0.2082989952
H	4.8208777230	1.7833668454	0.2266493700
O	5.2522442272	-0.6979755875	0.5515395575
C	5.4182537110	-2.0940385568	0.7202909848
H	6.4289152199	-2.2422852964	1.1211781588
H	4.6830164250	-2.5063461265	1.4324635101
H	5.3274589087	-2.6325678047	-0.2385471685

TS5b

0 1

C	0.4307089275	0.8160181637	-0.9744375892
H	-0.2352216954	-0.0916874853	-0.5201564886
O	-0.8541812167	-1.0734126701	0.3617059835
O	-2.8869449830	-2.8008636922	-0.4631905145
O	-3.4153439761	-0.6319539142	1.4342506862
O	-2.7756613268	-0.1247912327	-1.2098814592
Ru	-2.5677499025	-1.2358076204	0.0995301332
H	0.2475227183	0.7376549871	-2.0577472591
C	-1.2804500269	2.5595904931	-1.1014209184
C	-1.5098398078	2.7711177630	1.2696575735
C	-1.8975710168	3.4726637540	-0.0397292769
H	-1.9623801916	1.7131756101	-1.3516109228
H	-1.0189699578	3.0683473100	-2.0434208557
H	-2.1748802217	1.9117566812	1.4526727614
H	-1.5486814345	3.4298510095	2.1484542580
H	-2.9831973381	3.5819524648	-0.1697036490
H	-1.4472494570	4.4775755309	-0.0853728142
N	-0.1013272837	1.9961444496	-0.4641897676
C	-0.0896381188	2.2735622354	0.9776078746
H	0.1921468395	1.3673498076	1.5333575019
H	0.6555180854	3.0537939096	1.2126014366
C	1.8355359849	0.4626515266	-0.5883350219
C	2.7835797304	1.4498343906	-0.2612768708
C	2.2438502348	-0.8787203678	-0.5766486691
C	4.0897855628	1.1050030241	0.0634045450
H	2.4940511588	2.5019069242	-0.2809957837
C	3.5527349626	-1.2403565314	-0.2549318532
H	1.5195716619	-1.6617500514	-0.8119470559
C	4.4885496271	-0.2446561783	0.0728073187
H	4.8302073941	1.8685946983	0.3094294888
H	3.8296355985	-2.2938243603	-0.2550767425
O	5.7763124360	-0.4871554285	0.4044153082
C	6.2409827193	-1.8255193273	0.4313285253

H	7.2981845430	-1.7823036895	0.7215217453
H	5.6878337418	-2.4310724790	1.1692083876
H	6.1570930283	-2.3029017049	-0.5597280155
